# The chlorite adduct of aquacobalamin: contrast with chlorite dismutase

**DOI:** 10.1007/s00775-025-02100-5

**Published:** 2025-02-09

**Authors:** Maria Lehene, Cezara Zagrean-Tuza, Stefania D. Iancu, Sergiu-Raul Cosma, Adrian M. V. Brânzanic, Radu Silaghi-Dumitrescu, Bianca Stoean

**Affiliations:** 1 Department of Chemistry, Babeș-Bolyai University, Str. Arany Janos Nr. 11, 400028 Cluj-Napoca, Romania; 2 Faculty of Physics, Babeș-Bolyai University, Str. Kogalniceanu 1, 400084 Cluj-Napoca, Romania; 3 “Raluca Ripan” Institute for Research in Chemistry, Babeș-Bolyai University, Fântânele 30, 400294 Cluj-Napoca, Romania

**Keywords:** Chlorite, Cobalamin, UV–Vis, NMR, DFT, Chlorite dismutase

## Abstract

In the reaction of aquacobalamin (aquaCbl) with chlorite, a stable species is detected and assigned as a Co(III)–chlorite complex, Co(III)–OClO^−^. Its UV–Vis spectrum is almost identical to that of aquaCbl, except for some minor differences at ~ 430 nm; cyanide can eliminate and prevent these changes. The ^1^H-NMR spectra reveal strong influences of chlorite on the B2 and B4 protons of the cobalt-bound dimethyl benzimidazole ligand. Together, the UV–Vis and NMR titrations suggest a Kd of 10 mM or higher for chlorite on Cbl. Resonance Raman spectra reveal minor changes in the spectrum of aquaCbl to chlorite—as well as a disappearance of the free chlorite signals, consistent with Cbl–chlorite complex formation. Corroboration for these interpretations is also offered from mass spectrometry and DFT calculations. This Co(III)–OClO^−^ complex would be a stable analogue of the first reaction intermediate in the catalytic cycle of chlorite dismutase, or in the reaction of chlorite with a number of other heme proteins. The differences in reactivity between Co(III) cobalamin and Fe(III) heme towards chlorite are analyzed and rationalized, leading to a reconciliation of experimental and computational data for the latter.

**Supplementary Information:**

The online version contains supplementary material available at 10.1007/s00775-025-02100-5.

## Introduction

Cobalamins (Cbl) are one of the most complex vitamins regarding their structure. They actively participate in protein metabolism. The Co ion is ligated equatorially to the corrin ring and axial to a 5,6-dimethylbenzimidazole (DMBZ) connected to the corrin macrocycle. The sixth position is arranged to coordinate a variety of ligands. The redox chemistry of cobalt so far known, shows that it can adopt three oxidation stated: 3 + , 2 + , 1 +  [1–3].

Chlorite (ClO_2_^−^) is the strongest oxidizing agent of the chlorine oxyanions [4]. The oxyanions have applications in many fields of industry [5]. Chlorite reacts with several heme-containing enzymes [6]. For example, chlorite dismutase is a key enzyme in perchlorate and chlorate respiration, found in bacteria and archaea [7, 8]. It can reduce chlorite into the environmentally innocuous chloride anion while producing molecular oxygen [9]. Chlorite can be utilized in producing oxidized enzymes intermediates from peroxidases. Among the heme peroxidases that can use chlorite, cytochrome *c* peroxidase, chloroperoxidase and horseradish peroxidase are also included [10, 11]. The formation of methemoglobin is induced by chlorite and it might also act as a hydroxylating agent in cytochrome P450 [12]. In addition, other possible effects of chlorite are modulation and stimulation of immune responses, activation of macrophage functions and the stimulation of killer cell cytotoxicity [13–15]. Chlorite is also an active component in wound healing drugs such as WF10, where it converts oxyhemoglobin into methemoglobin [16].

The reactivity of cobalamin and related co-corrinoid complexes towards oxidizing agents has generally been described to involve corrin degradation, but—unlike with Fe biological centers such as heme-porphyrin complexes—no formation of Co complexes with the oxidizing agent has been reported, and no formation of high-valent species. However, more recently we have shown that hydrogen peroxide does form a stable and reversible complex, assigned as Co(III)–hydroperoxo [17]. The reaction of cobalamin with chlorine oxyanions has been described in the case of chlorite and hypochlorite and leads to destruction of cobalamin [15, 18]. In case of chlorite, it does not react with aquaCbl, while Cbl(II) reacts with chlorite and oxidizes the Co(II)-center [15]. Hypochlorite is known for corrin degradation of cyanocobalamin [18, 19], but more recently it has been shown that both aqua- and cyanocobalamin react with hypochlorite and form intermediates with a new Co(III)–OCl bond. [20]

Reported here is a spectroscopic, kinetic and computational analysis of the reaction of aquacobalamin with chlorite, showing that a stable Co(III) complex can be detected. A comparison with the Fe(III)–chlorite complexes proposed to serve as intermediate in the catalytic cycle of chlorite dismutase is provided, and the catalytic cycle of the latter is rationalized in order to remove conflicts between experiment and theory.

## Materials and methods

Hydroxocobalamin hydrochloride (HOCbl, ≥ 98%), cyanocobalamin (NCCbl, ≥ 98%), sodium cyanide (≥ 95%) and sodium chlorite (p.a., 80%; further purification as described in reference [15] did not improve this percentage) were obtained from Sigma-Aldrich (Munich, Germany) and used as received. The buffer solution used was a universal pH buffer, Britton–Robinson, it consists of a mixture of 0.04 M boric acid, 0.04 M phosphoric acid and 0.04 M acetic acid, that has been titrated to pH 7 with 0.2 M sodium hydroxide.

UV–Vis spectra were performed on a Cary 50 UV–Vis spectrophotometer (Varian, Inc., Foster City, CA, USA).

Raman spectra were measured on a Renishaw inVia Raman spectrometer coupled with a Leica microscope at 22 °C. 10 μl of each sample was dropped on a microscope slide covered with aluminum foil. The 532 nm laser line with a power of 100 mW was focused on the sample using a 5X objective. Each spectrum is represented as an average of 4 accumulations and 4 s.

NMR spectra were recorded at 20 °C unless otherwise stated, after diluting the sample (at concentrations indicated in text and Figure legends) with D_2_O, on a 500 MHz Bruker instrument. The solvent used was Britton–Robinson universal buffer at pH 7 prepared in D_2_O. A water-suppression pulse sequence was used for these measurements.

High-resolution mass spectra (HRMS) were recorded on an LTQ ORBITRAP XL mass spectrometer (ThermoScientific) using positive electrospray ionization. The instrument was externally calibrated. The samples were prepared at room temperature (22 °C) and then inserted into the instrument immediately. The following conditions were used: source voltage, 3.2 kV; sheath and auxiliary gas flow, 8 and 5 arbitrary units, respectively; vaporizer temperature 50 °C, capillary temperature 275 °C, analyzer temperature 26 °C; capillary voltage, 28 V; tube lens voltage, + 110 V. The number of microscans was set to three.

For DFT calculations, the Gaussian09 software package [21] was employed following the methodology previously described for Cbl complexes [17]. The Cbl models were truncated, with the lateral substituents on the corrin as well as the methyl groups on the benzimidazole replaced by hydrogen. Gas-phase geometries and frequency analyses were computed with the aid of the B3PW91 [22, 23] functional at the def2-SV(P) [24] double-zeta basis set level. Long-range interactions were accounted by the use of Grimme’s D3 dispersion correction [24]. Population analyses, NMR [25] and TD-DFT derived [25] UV–Vis spectra were computed in the C-PCM solvent continuum adapted for aqueous environment [26]. In terms of methodology choice for DFT calculations, the methodology employed here was selected for its ability to best mimic trends in UV–Vis spectra as described in our previous study on hydroperoxocobalamin, thus also allowing consistency between the two sets of data [27]. DFT-derived spectral data were obtained using Chemcraft [28]; for the Raman simulations, 298 K and an excitation wavelength of 22,000 cm^−1^ were assumed.

## Results and discussion

**Fig. 1:**
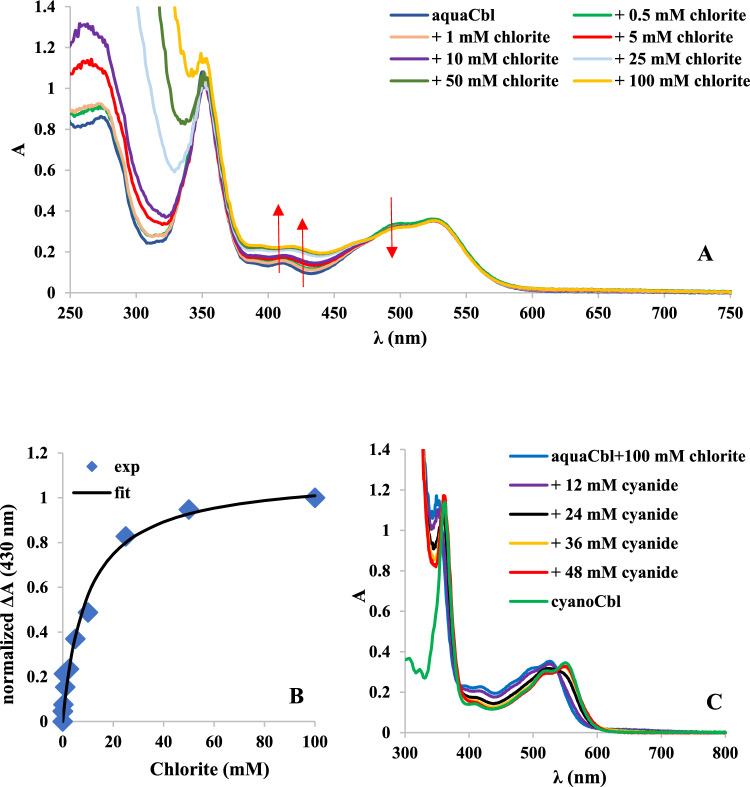
**A** UV–Vis spectra of the reaction between 50 μM aquaCbl and 0.5 mM–100 mM chlorite at pH 7, room temperature. **B** titration curve for aquacobalamin reacting with chlorite at pH 7, monitored at 430 nm and fitted to a simple hyperbolic equation (normalized ΔA = [chlorite]/(Kd + [chlorite]). **C** UV–Vis spectra of aquaCbl reacting with 100 mM chlorite, compared to aquaCbl reacting with 12 mM-48 mM cyanide and cyanoCbl (see also Figures S7 and S8)

Figure 1 shows that chlorite up to 100 mM has no clear effect on the UV–Vis spectrum of aquacobalamin above 450 nm. In the 250–350 nm, an increase in absorbance is seen due to chlorite, but no change in the 350-nm band of Cbl. The slight changes in the 400–450 nm region may partially be due to chlorite as well; however, the 415-nm maximum of aquacobalamin appears slightly redshifted and less defined at higher chlorite concentrations. Such small changes in the 400–450 nm region were previously shown to reflect a change in coordination of Co(III) Cbl when hydrogen peroxide replaces the axial water ligand [17]. Fig. 1 also shows that these changes, monitored at 430 nm, show a saturation behavior with an apparent K_d_ of 9.6 mM, which may be taken as evidence for formation of a Co(III)–chlorite complex. As also shown in Fig. 1, addition of cyanide to the proposed Co(III)–chlorite cobalamin complex leads to formation of cyanocobalamin; these spectra may be interpreted as evidence that cyanide displaces chlorite from Co(III)—and that chlorite has not induced any detectable degradation of Cbl at these concentrations.

**Fig. 2:**
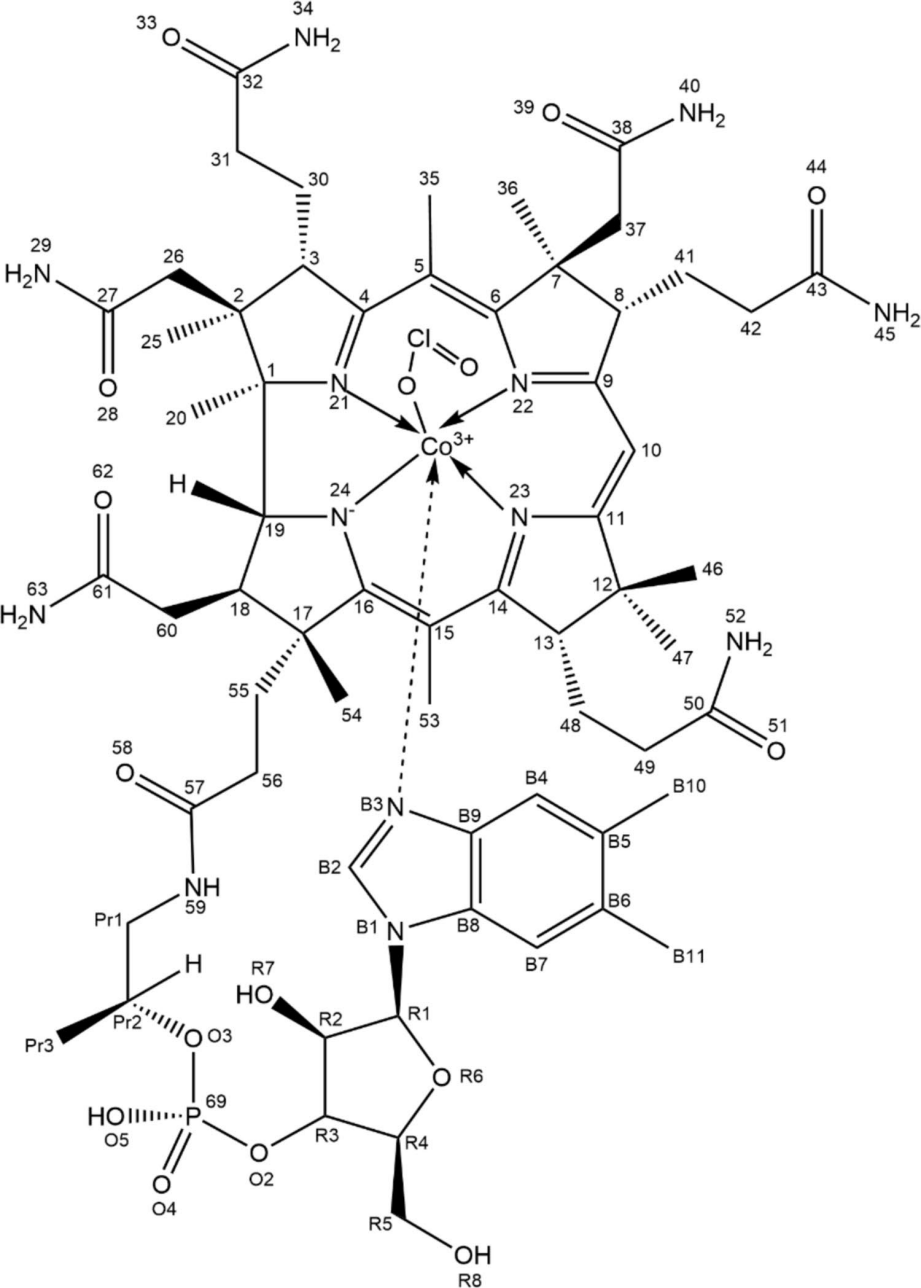

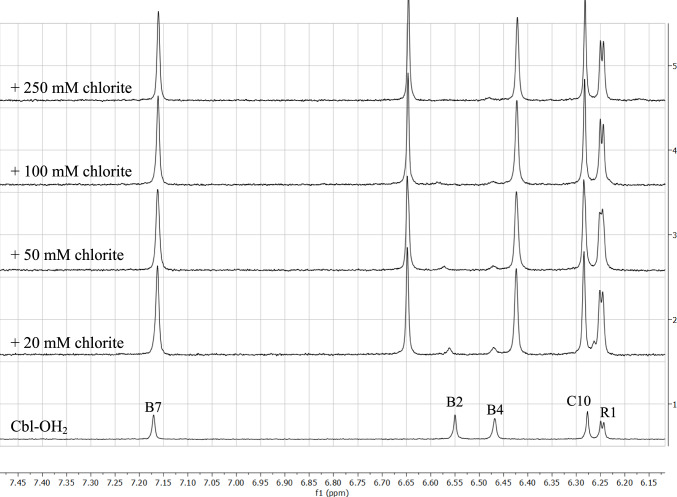
Upper panel: proposed structure of the Cbl–chlorite complex. Lower panel: ^1^H-NMR spectra of 1 mM aquaCbl with chlorite at pH 7. See also Figure S9, and Tables S1 and S2 for more detailed information

Clearer evidence for chlorite complexation to Cbl is provided by the ^1^H-NMR spectra in Fig. 2. The changes are most visible at the B2 and B4 protons (6.55 and 6.47 ppm, respectively, protons which, as also seen in Fig. 2, are the closest to the Co within the Co-bound benzimidazole ligand), with shifts of ~ 0.1 ppm each. Overall, the changes are much smaller than those seen for the peroxide complex of Cbl [17, 27]. Aquacobalamin signals disappear completely at 50 mM chlorite—in good agreement with the UV–Vis titration shown in Fig. 1. No additional signals are seen up to 250 mM chlorite, suggesting that only one chlorite complex/isomer is formed/detectable and that the corrin has remained essentially intact.

**Fig. 3:**
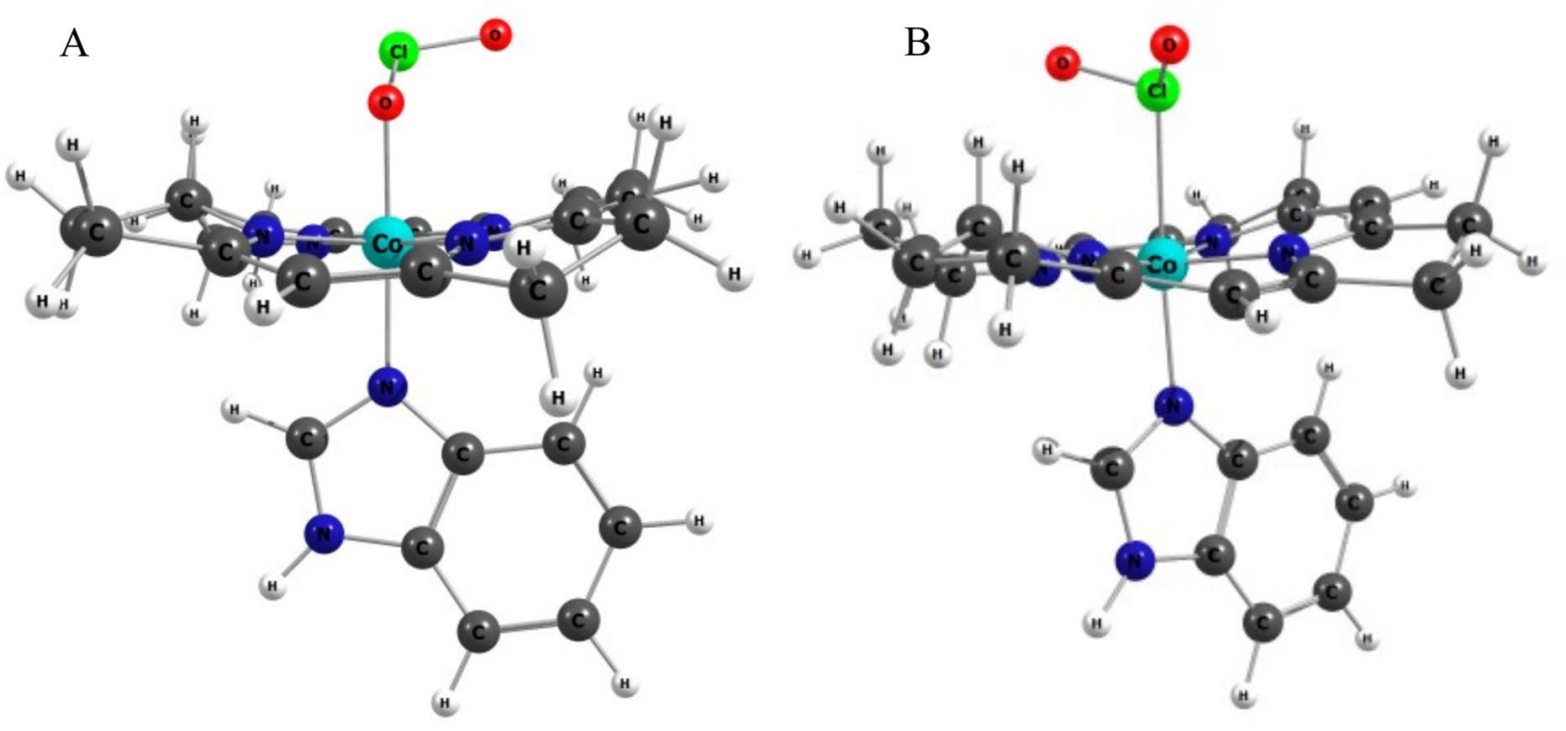
DFT-optimized geometries for Cbl–chlorite isomers, bound via O (A) and Cl (B), respectively

According to density functional theory calculations (DFT) shown in Fig. 3 and Table 1 (and Figures S1 and S2), the two possible linkage isomers of the Co(III)–chlorite complex in cobalamin are both reasonable local energy minima, with geometrical parameters similar to those seen in other complexes of transition metals with chlorite. Binding via oxygen is favored by ~ 9 kcal/mol. The UV–Vis maxima predicted from TD-DFT calculations with the best available methodology (detailed in [17]) are still not able to mimic the experimental spectrum, but, as discussed before, do allow a qualitative interpretation. According to Fig. 1, the chlorite adduct has a spectrum almost indistinguishable from that of aquacobalamin. For the latter, the TD-DFT calculations predict the two major maxima at 310 and 434 nm; the O–chlorite isomer in Table 1 is a much better match to these values (with its maxima predicted at 310 and 426 nm cf. Table 1) than the Cl–chlorite isomer (301 and 416 nm cf. Table 1). Hence, the DFT calculations, also shown in Table 2, corroborate the experimental data and support the hypothesis of a Co(III)–O–Cl–O^−^ complex. Further calculations on models of Cbl–chlorite with increasingly elongated CoO–ClO bonds reveal that elongation by up to 1 Å would entail an energy cost of ~ 40 kcal/mol, which is only 20% smaller than what is seen for another Co(III) complex with an oxidizing agent, hydrogen peroxide [27].

**Table 1:** DFT-derived data (key bond lengths in Å, and relative energies in kcal/mol, where applicable) of Cbl–chlorite, bond via O and via Cl truncated models and in parenthesis for the big models. TD-DFT-derived wavelengths (nm) and oscillator strengths (OS) for Cbl–chlorite models, the two most intense bands above 300 nm are reported; see Figures S1 and S2 for orbitals involved in these transitions

Isomer	dE (kcal/mol)	Co–L (Å)	O–Cl (Å)	Cl–O (Å)	Co–DMBZ (Å)	UV–Vis maxima (nm/OS)
Co–O–Cl–O	0.0	1.92 (1.95)	1.70 (1.73)	1.58 (1.58)	2.01 (2.09)	310/0.1180 426/0.0995
Co–ClO_2_	8.6 (17.2)	2.38 (2.38)	1.56 (1.55)	1.56 (1.56)	2.00 (1.98)	301/0.1680 416/0.0967

**Table 2:** ^1^H-NMR peak assignments (in ppm) for Cbl–chlorite measured (exp.) and computed (DFT)

Assignment	(OH_2_)			(OH^−^)			(OClO^−^)		(ClO_2_^−^)
	Ref. [29]	exp	DFT	Ref. [29]	exp	DFT	exp	DFT	DFT
B7	7.16	7.17	7.47	7.17	7.16	7.49	7.16	7.46	7.45
B2	6.51	6.55	6.54	6.74	6.73	7.16	6.64	7.02	6.67
B4	6.44	6.47	7.05	6.50	6.49	7.30	6.42	7.21	7.03
C10	6.26	6.28	6.71	6.07	6.07	6.35	6.28	6.40	6.47
R1	6.22	6.25	6.32	6.25	6.25	6.37	6.25	6.34	6.31

**Fig. 4:**
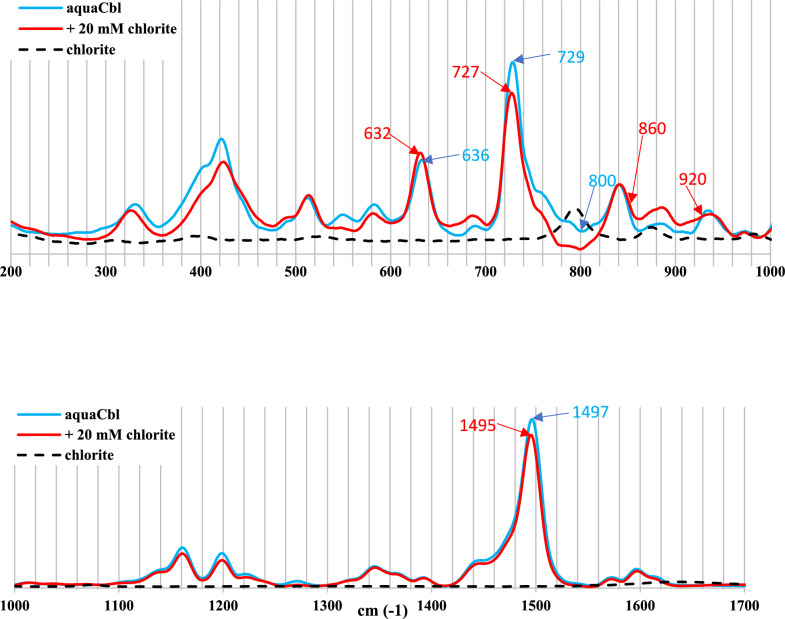
Resonance Raman spectra of 1 mM aquaCbl in the presence or absence of 20 mM chlorite at pH 7, room temperature. Shown for reference is also the control spectrum of 20 mM chlorite alone, without cobalamin

**Fig. 5:**
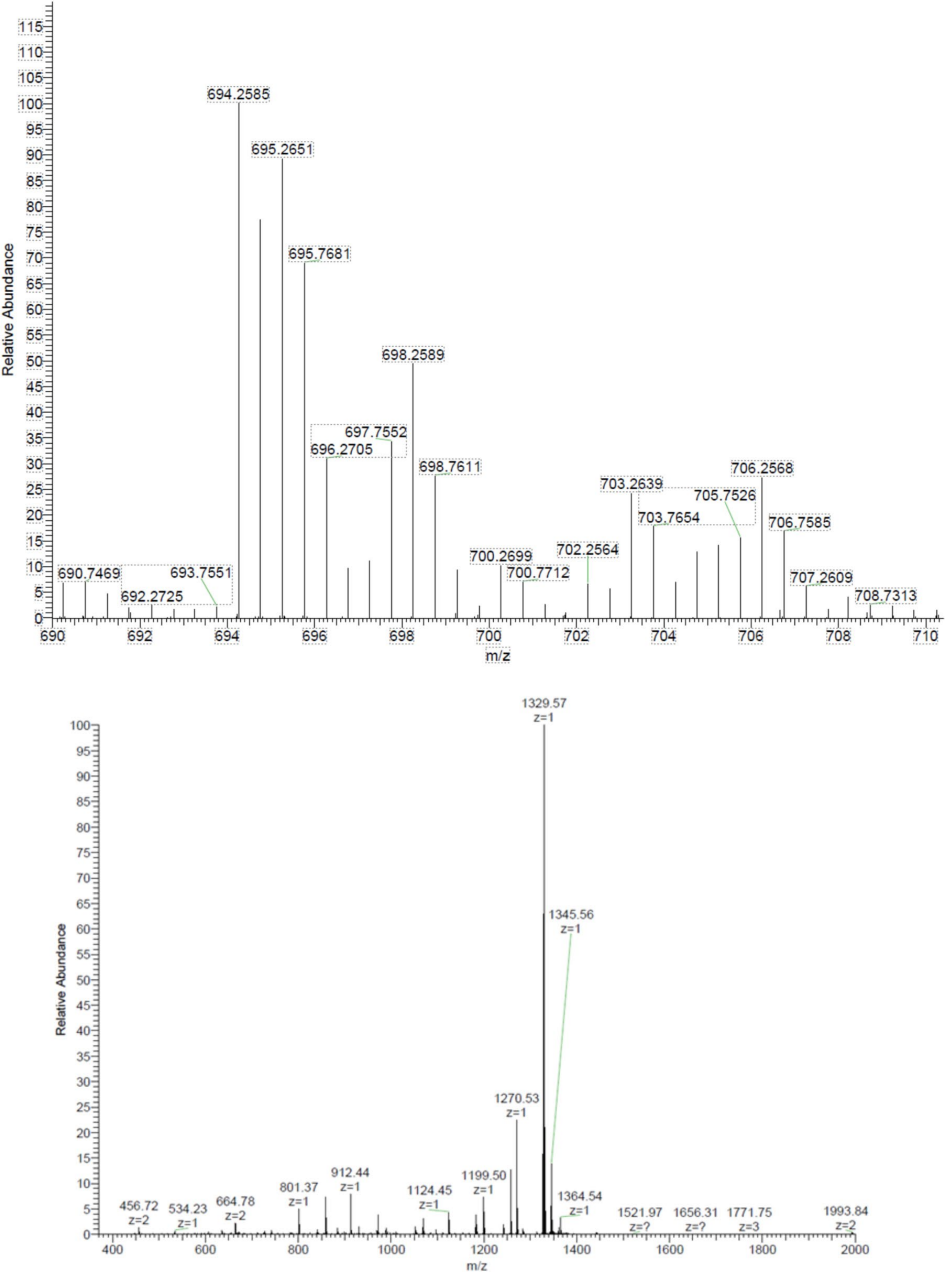
Upper panel: ESI( +)-HRMS (m/z) calculated for C_62_H_88_CoN_13_O_16_PCl 1397.52 (z = 2 697.76), found: 697.76. Conditions: 1 mM H_2_OCbl^+^, 100 mM chlorite, 50 mM phosphate pH 7. Lower panel: spectrum of starting material (hydroxocobalamin). Conditions: 1 mM H_2_OCbl^+^, 50 mM phosphate pH 7

The resonance Raman spectra of aquacobalamin are dominated by the corrin chromophore whose structure is largely unaffected by axial ligation (see also Figures S3-S5) and hence, as also seen previously with other ligands, show only very small changes upon reaction with chlorite (cf. Figure 4) [17, 27]. Nevertheless, the changes seen at 1495 cm^−1^ (carbon–carbon stretching inside the corrin ring) and 727 cm^−1^ are consistent with binding of a new ligand (presumably chlorite) with Cbl. In line with this interpretation, the ~ 800 cm^−1^ band of free chlorite disappears upon mixing with cobalamin; instead, the cobalamin–chlorite mixture displays an increase in intensity at 860–920 cm^−1^. The high-resolution mass spectrum of the cobalamin-chlorite adduct reveals the expected molecular peak at 2 × 697.76, as shown in Fig. 5—although its abundance is distinctly smaller than previously reported for other Cbl complexes under similar conditions; these spectra, as discussed before, are otherwise complicated by the inherently harsh conditions compared to those in the UV–Vis or NMR experiments [17, 27].

The complex of aquaCbl with chlorite C_62_H_88_CoN_13_O_16_PCl appears in the HRMS (ESI +) spectrum at m/z = 697.2523 with a relative abundance of approximatively 12% and the corresponding singly protonated form with the same 2 + charge appears at 697.7552.

The presence of the chlorine atom from the structure of the chlorite ligand is demonstrated by the specific isotopic pattern: for every present peak identified in the spectrum, there are two signals which have a two mass units difference, with an intensity report of approximatively 3:1 (for example: m/z = 694.2585 and m/z = 696.2705; m/z = 698.2589 and m/z = 700.2699).

The signals appearing at lower m/z values compared to the 2 + molecular ion peak are obtained through deprotonation. Thus, the signal from [M-2H^+^] is seen at m/z = 696.2705, the signal from [M-4H^+^] is seen at m/z = 695.2651 and the signal from [M-6H^+^] is seen at m/z = 694.2585.

Beside the molecular peak with + 2 charge from m/z = 697.2523, additional peaks appear at higher values with 0.5 mass unit difference, assigning the presence of the doubly charged species, which are attributed for the protonation of the groups that have non-participant electrons as: terminal amides or primary alcohols. For example, the signal from [M + H^+^] is seen at m/z = 697.7552, the signal from [M + 2H^+^] is seen at 698.2589 and the signal from [M + 3H^+^] is seen at 698.7611. All these retain the isotopic pattern specific to the chlorine atom.

**Fig. 6:**
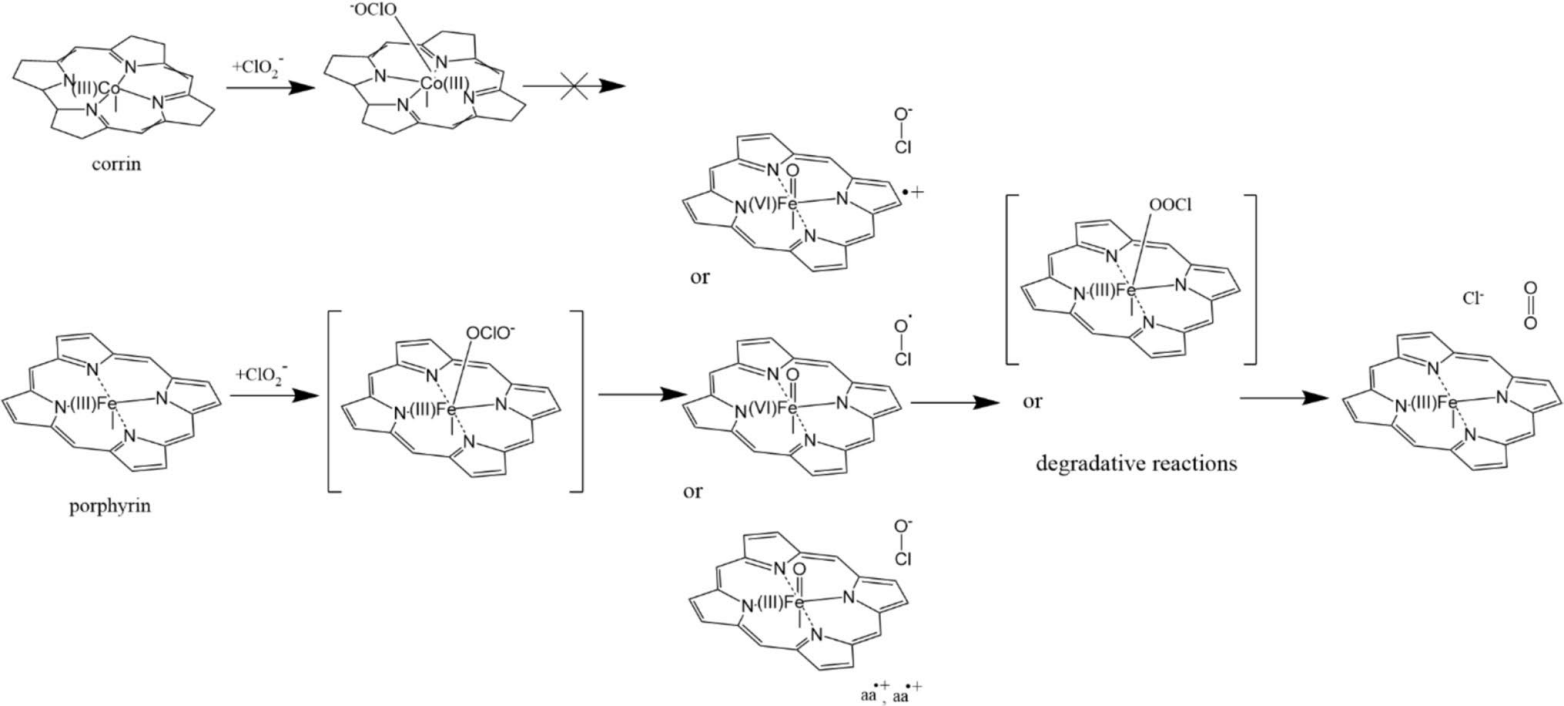
Chlorite reactivity in cobalamin vs. hemoproteins (e.g., chlorite dismutase, myoglobin)

Figure 6 summarizes the contrast between cobalamin and another biologically important metallamacrocycle complex, heme, in terms of reactivity towards chlorite. The above-reported data support a stable Co(III) chlorite adduct in cobalamin. Hemoproteins such as chlorite dismutase (Cld) or myoglobin (Mb) are proposed to also bind chlorite at their metallacycle active site; however, such a heme(protein)-chlorite complex has not been observed experimentally. Instead, UV–Vis and electron paramagnetic resonance (EPR) data have shown evidence for O–Cl bond cleavage, leading to one of three proposed structures: Compound I (ferryl + porphyrin cation radical) + hypochlorite, Compound II (ferryl) + hypochloryl radical, or Fe(III) + two neighboring aminoacid radicals. In chlorite dismutase, the OCl units rebounds on the iron-coordinated oxygen atom to form an (as yet putative) peroxo intermediate, after which the terminal O–Cl bond is broken, yielding molecular oxygen and chloride [7, 9, 30–36]. In myoglobin, protein degradation ensues from the ferryl stage [37]. DFT calculations have identified the S = 1/2 Fe(III)–O–Cl–O^−^ and Fe(III)–O–O–Cl^−^ species as energy minima on the potential energy surface, with barriers of at most ~ 10 kcal/mol for subsequent O–Cl bond cleavage—which is consistent with the fact that even in experiments with deadtimes as low as 0.1 ms these species were not reported to be observed [33, 34, 36]. As shown in Table 1, these results obtained using the B3LYP functional can be mirrored by another functional, TPSS. However, in the latter case geometry optimization leads to O–Cl bond cleavage even upon geometry optimization, further reinforcing the idea that these two intermediates would not be observable. The initial product of O–Cl cleavage has been suggested by computational studies (including the data shown here in Supporting Information, Figure S6, Tables S3-S6) to be a ferryl heme (Compound II) with a hypochloryl radical [9, 31, 33, 34, 36, 38–40]. Instead, experimental measurements have identified three types of reaction intermediates in mixtures of hemoproteins with chlorite, all resulting from O–Cl bond cleavage within Fe(III)–O–Cl–O: (1) at sub-millisecond timescales in Cld, a ferric heme and an unusual doublet free radical, assigned as the lowermost intermediate in Fig. 6 (a ferric-hydroxo heme alongside two closely spaced aminoacid radicals), (2) at low-millisecond time scales in Cld, a Compound I species (presumably alongside a hypochlorite anion), and (3) at millisecond to second time scale in Cld as well as in Mb, a ferryl species (Compound II) [34, 36]. The ferric EPR signals seen at sub-milliseconds are either high-spin or with large tetragonality, both of which are not consistent with a ferric-hydroxo heme. In fact, the low-spin heme shows g-values nearing 3.0, consistent with a hemichrome (with nitrogen ligation at the sixth coordination position) rather than with ligation with a softer oxygen ligand such as chlorite or hydroxide). Ferryl heme, also expected as possible intermediate at this stage, would show no EPR signals, while the free radical signal seen in these sub-millisecond EPR spectra may in light of the above discussions be assigned as the hypochloryl radical—though its complex shape and the scarcity of EPR data on related radicals may warrant further investigation. If so, then the Compound I and Compound II species observed in stopped-flow UV–Vis and freeze-quench EPR measurements at millisecond timescales in Cld as well as (for Compound II) in Mb [36, 37] are likely not part of the Cld catalytic cycle, but rather inevitable byproducts due to the extremely high concentrations of chlorite in those experiments (up to 1 M).

It has previously been reported that Cbl(II) reacts with chlorite and oxidizes Cbl(II) to aquaCbl(III) as a major product and corrin-modified species as minor products, while a reaction of Cbl(III) with chlorite could not be observed using UV–Vis spectroscopy [15]. These latter observations are confirmed by the present study, where the effects of chlorite on the UV–Vis spectrum of cobalamin are minor and only observable at relatively high chlorite concentrations. In fact, the formation of an adduct between Cbl(III) and chlorite is only confirmed with the use of NMR spectra of the present study.

Chlorite forms a stable Co(III) complex with cobalamin as evidenced by UV–Vis, NMR, MS, resonance Raman and DFT. The stability of this complex is in contrast to what is seen in ferric hemes, where (for chlorite dismutase, at least) O–Cl bond cleavage appears to take less than 100 μs. As in the case of O–O bond cleavage in Fe/Co-peroxide complexes, the increased ease of O–Cl bond cleavage in Fe(III) compared to Co(III) relates to the difference in M(III)/M(IV) redox potentials (i.e., Co(IV) much less than stable/likely Fe(IV)).

## Supplementary Information

Below is the link to the electronic supplementary material.

Supplementary file1 (DOCX 15852 KB)

## Data Availability

Data is provided within the manuscript and supplementary information files.

## References

[1] Dereven’kov IA, Makarov SV, Shpagilev NI, Salnikov DS, Koifman OI (2017) Biometals 30:57–764 10.1007/s10534-017-0044-8 28836023

[2] Dassanayake RS, Farhath MM, Shelley JT, Basu S, Brasch NE (2016) J Inorg Biochem 163:81–87 27567143 10.1016/j.jinorgbio.2016.07.009

[3] Derevenkov IA, Salnikov DS, Silaghi-Dumitrescu R, Makarov SV, Koiffman OI (2016) Coord Chem Rev 309:68–83

[4] Hicks SD, Kim D, Xiong S, Medvedev GA, Caruthers J, Hong S, Nam W, Abu-Omar MM (2014) J Am Chem Soc 136:3680–3686 24498903 10.1021/ja5001642

[5] Liebensteiner MG, Oosterkamp MJ, Stams AJM (2016) Ann N Y Acad Sci 1365:59–72 26104311 10.1111/nyas.12806

[6] Zámocký M, Hofbauer S, Schaffner I, Gasselhuber B, Nicolussi A, Soudi M, Pirker KF, Furtmüller PG, Obinger C (2015) Arch Biochem Biophys 574:108–119 25575902 10.1016/j.abb.2014.12.025 PMC4420034

[7] de Geus DC, Thomassen EAJ, Hagedoorn PL, Pannu NS, van Duijn E, Abrahams JP (2009) J Mol Biol 387:192–206 19361444 10.1016/j.jmb.2009.01.036

[8] T. P. Barnum and J. D. Coates, ISME J. 2022 171, 2022, **17**, 70–83. 10.1038/s41396-022-01317-5 PMC9751292 36202926

[9] Hofbauer S, Schaffner I, Furtmüller PG, Obinger C (2014) Biotechnol J 9:461–473 24519858 10.1002/biot.201300210 PMC4162996

[10] Wilson I, Brewher KR, Chea CK, Kelly HC (1983) heme models of peroxidase enzymes: deuteroferriheme-catalyzed chlorination of monochlorodimedone by sodium chlorite. J Inorg Biochem 19:345 6655473 10.1016/0162-0134(83)80008-7

[11] Jakopitsch C, Pirker KF, Flemmig J, Hofbauer S, Schlorke D, Furtmüller PG, Arnhold J, Obinger C (2014) J Inorg Biochem 135:10–19 24632343 10.1016/j.jinorgbio.2014.02.010 PMC4003552

[12] Hrycay EG, Gustafsson JÅ, Ingelman-Sundberg M, Ernster L (1975) FEBS Lett 56:161–165 169152 10.1016/0014-5793(75)80132-3

[13] Payne H, Adamson A, Bahl A, Borwell J, Dodds D, Heath C, Huddart R, McMenemin R, Patel P, Peters JL, Thompson A (2013) BJU Int 112:885–897 24000900 10.1111/bju.12291 PMC4155867

[14] Giese T, McGrath MS, Stumm S, Schempp H, Elstner E, Meuer SC (2004) Cell Immunol 229:149–158 15474529 10.1016/j.cellimm.2004.08.001

[15] Dereven’kov IA, Shpagilev NI, Valkai L, Salnikov DS, Horváth AK, Makarov SV (2017) J Biol Inorg Chem 22:453–459 27864634 10.1007/s00775-016-1417-0

[16] Pichert A, Arnhold J (2015) Arch Biochem Biophys 585:82–89 26391926 10.1016/j.abb.2015.09.009

[17] Lehene M, Plesa D, Ionescu-Zinca S, Iancu SD, Leopold N, Makarov SV, Brânzanic AMV, Silaghi-Dumitrescu R (2021) Inorg Chem 60:12681–12684 34382784 10.1021/acs.inorgchem.1c01483

[18] Abu-Soud HM, Maitra D, Byun J, Souza CEA, Banerjee J, Saed GM, Diamond MP, Andreana PR, Pennathur S (2012) Free Radic Biol Med 52:616–625 22138102 10.1016/j.freeradbiomed.2011.10.496 PMC3786219

[19] Dereven’kov IA, Osokin VS, Hannibal L, Makarov SV, Khodov IA, Koifman OI (2021) J Biol Inorg Chem 26:427–434 33914169 10.1007/s00775-021-01869-5

[20] Lehene M, Brânzanic AMV, Silaghi-Dumitrescu R (2023) J Biol Inorg Chem 28:583–589 37493822 10.1007/s00775-023-02015-z

[21] Frisch MJ, Trucks GW, Schlegel HB, Scuseria GE, Robb MA, Cheeseman JR, Scalmani G, Barone V, Petersson GA, Nakatsuji H, Li X, Caricato M, Marenich A, Bloino J, Janesko BG, Gomperts R, Mennucci B, Hratchian HP, J. V. Ortiz, A. F. Izmaylov, J. L. Sonnenberg, D. Williams-Young, F. Ding, F. Lipparini, F. Egidi, J. Goings, B. Peng, A. Petrone, T. Henderson, D. Ranasinghe, V. G. Zakrzewski, J. Gao, N. Rega, G. Zheng, W. Liang, M. Hada, M. Ehara, K. Toyota, R. Fukuda, J. Hasegawa, M. Ishida, T. Nakajima, Y. Honda, O. Kitao, H. Nakai, T. Vreven, K. Throssell, J. A. MontgomeryJr., J. E. Peralta, F. Ogliaro, M. Bearpark, J. J. Heyd, E. Brothers, K. N. Kudin, V. N. Staroverov, T. Keith, R. Kobayashi, J. Normand, K. Raghavachari, A. Rendell, J. C. Burant, S. S. Iyengar, J. Tomasi, M. Cossi, J. M. Millam, M. Klene, C. Adamo, R. Cammi, J. W. Ochterski, R. L. Martin, K. Morokuma, O. Farkas, J. B. Foresman and D. J. Fox, Gaussian 09, Revis. E.01, Gaussian, Inc., Wallingford CT, 2016.

[22] Becke AD (1993) J Chem Phys 98:5648–5652

[23] Lee C, Yang W, Parr RG (1988) Phys Rev B 37:785–789 10.1103/physrevb.37.785 9944570

[24] Schäfer A, Horn H, Ahlrichs R (1992) J Chem Phys 97:2571–2577

[25] Ditchfield R (1974) Mol Phys 27:789–807

[26] Barone V, Cossi M (1998) J Phys Chem A 102:1995–2001

[27] Lehene M, Zagrean-Tuza C, Hadade ND, Aghion A, Septelean R, Iancu S, Branzanic AM, Silaghi-Dumitrescu R (2023) New J Chem 47:18178–18185

[28] Chemcraft - graphical software for visualization of quantum chemistry computations. Version 1.8, build 682. https://www.chemcraftprog.com

[29] Calafat AM, Marzilli LG (1993) J Am Chem Soc 115:9182–9190

[30] Shahangian S, Hager LP (1982) J Biol Chem 256:11529–11533 7118894

[31] Keith JM, Abu-Omar MM, Hall MB (2011) Inorg Chem 50:7928–7930 21806042 10.1021/ic2009732

[32] Kostan J, Sjöblom B, Maixner F, Mlynek G, Furtmüller PG, Obinger C, Wagner M, Daims H, Djinović-Carugo K (2010) J Struct Biol 172:331–342 20600954 10.1016/j.jsb.2010.06.014

[33] Lee AQ, Streit BR, Zdilla MJ, Abu-Omar MM, DuBois JL (2008) Proc Natl Acad Sci 105:15654–15659 18840691 10.1073/pnas.0804279105 PMC2572943

[34] Mayfield JA, Blanc B, Rodgers KR, Lukat-Rodgers GS, DuBois JL (2013) Biochemistry 52:6982–6994 24001266 10.1021/bi4005599 PMC4390081

[35] De Schutter A, Correia HD, Freire DM, Rivas MG, Rizzi A, Santos-Silva T, González PJ, Van Doorslaer S (2015) J Phys Chem B 119:13859–13869 26287794 10.1021/acs.jpcb.5b04141

[36] Püschmann J, Mahor D, de Geus DC, Strampraad MJF, Srour B, Hagen WR, Todorovic S, Hagedoorn P-L (2021) ACS Catal 11:14533–14544 34888122 10.1021/acscatal.1c03432 PMC8650003

[37] Bischin C, Mot A, Stefancu A, Leopold N, Hathazi D, Damian G, Silaghi-Dumitrescu R (2017) J Inorg Biochem 172:122–128 28458145 10.1016/j.jinorgbio.2017.04.017

[38] S. Sun, S.-L. Chen and Z.-S. Li, Int. Photonics Optoelectron. Meet. 2013, ASa3A.25.

[39] Sun S, Li Z-S, Chen S-L (2014) Dalt Trans 43:973–981 10.1039/c3dt52171k 24162174

[40] Su J-X, Chen S-L (2017) J Catal 348:40–46

