# Supplementary material for: The chlorite adduct of aquacobalamin: contrast with chlorite dismutase
Source: J Biol Inorg Chem. 2025 Feb 9;30(1):25–34. doi: 10.1007/s00775-025-02100-5 (PMC11913970; doi:10.1007/s00775-025-02100-5)
Supplement: Supplementary file 1 — Supplementary file1 (DOCX 15852 KB) [file 775_2025_2100_MOESM1_ESM.docx]

**The chlorite adduct of aquacobalamin: contrast with chlorite dismutase**

Maria Lehene,^1^ Cezara Zagrean-Tuza,^1^ Stefania D. Iancu,^2^ Sergiu-Raul Cosma,^1^ Adrian M.V. Brânzanic,^3,4^ Radu Silaghi-Dumitrescu^1*^, Bianca Stoean (Vasile)^1^

*^1^Department of Chemistry, Babeș-Bolyai University, Str. Arany Janos Nr. 11, RO-400028 Cluj-Napoca, Romania.*

*^2^Faculty of Physics, Babeș-Bolyai University, Str. Kogalniceanu 1, RO-400084 Cluj-Napoca, Romania.*

*^3^Intedisciplinary Research Institute on Bio-Nano Sciences, Babeș-Bolyai University, Cluj-Napoca, Romania.*

*^4^Raluca Ripan Institute for Research in Chemistry, Babeș-Bolyai University.*

**Correspondence to: radu.silaghi@ubbcluj.ro*


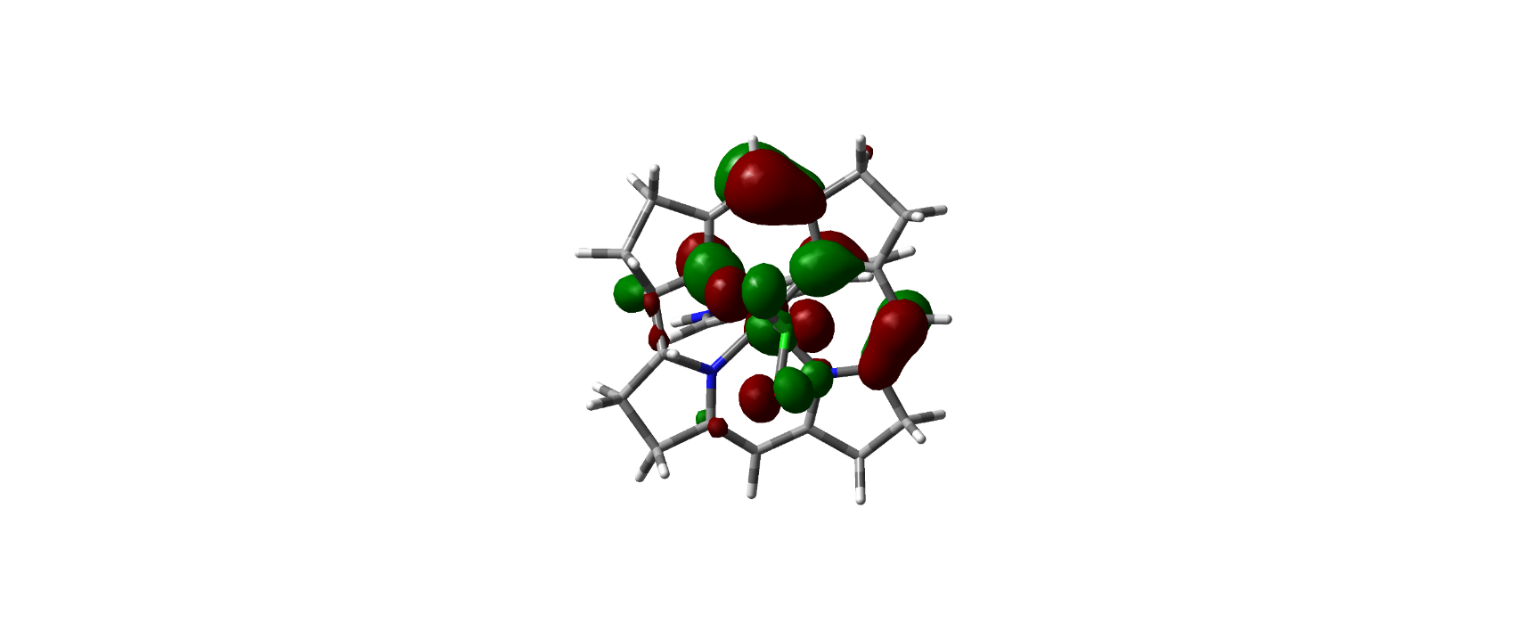

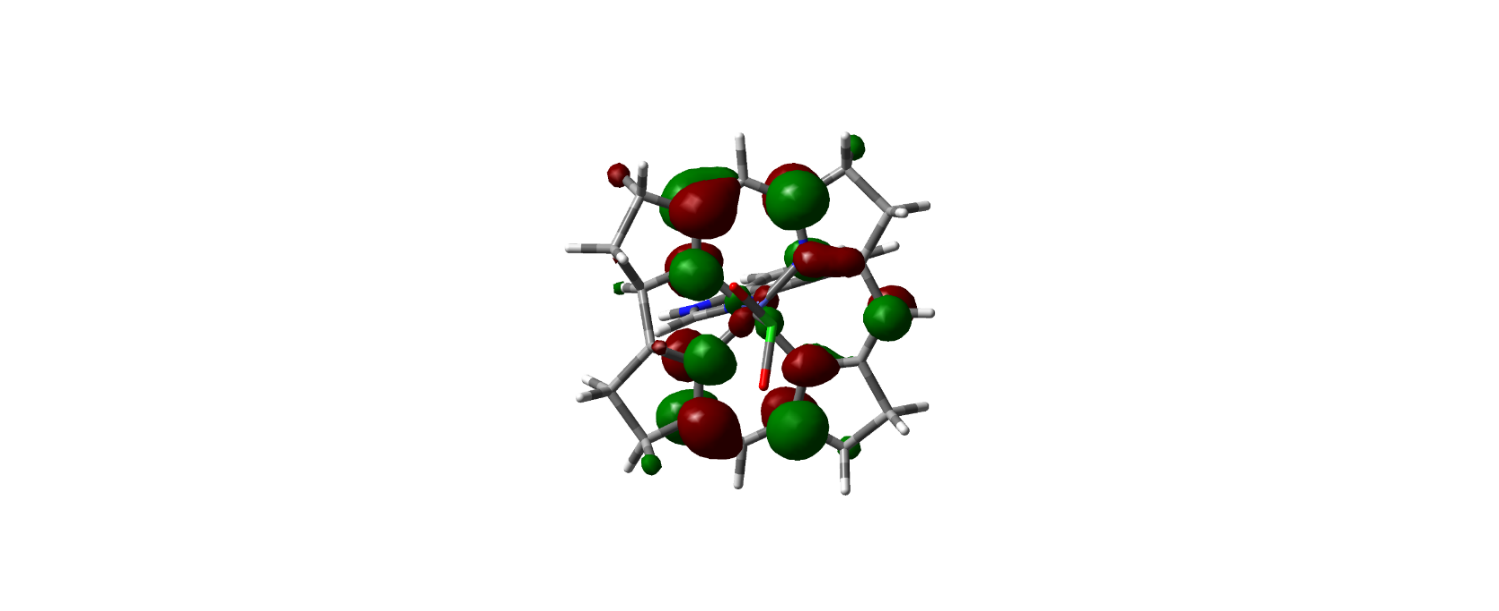


301 nm

HOMO-1 LUMO+2


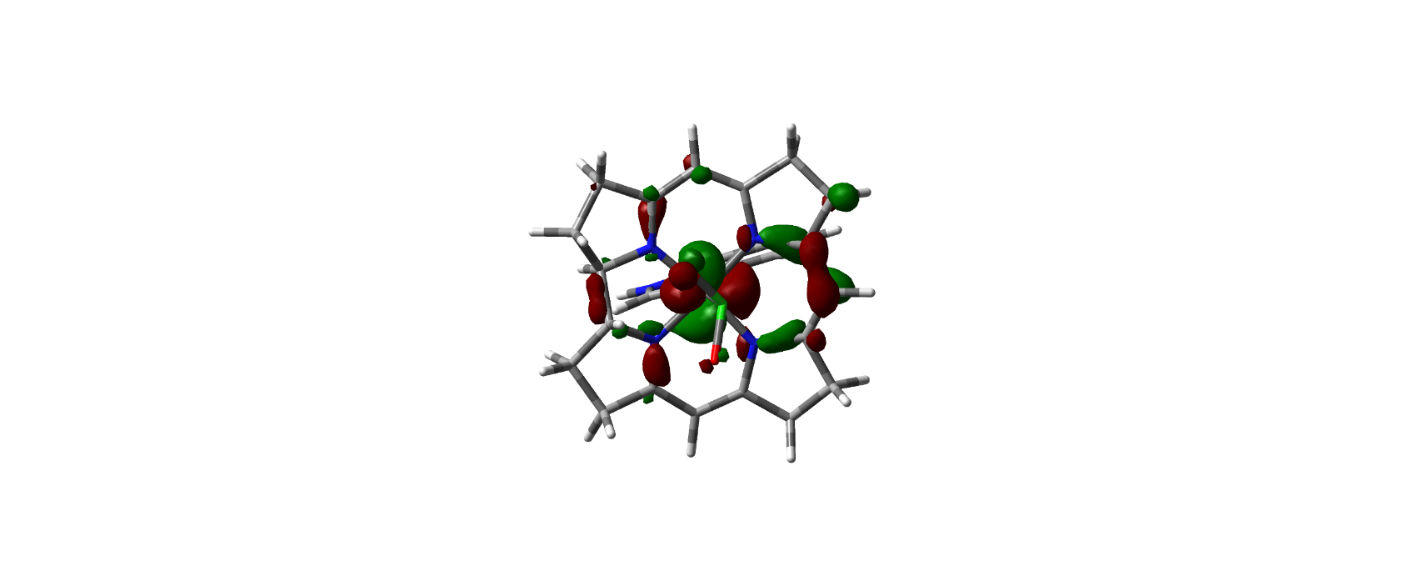

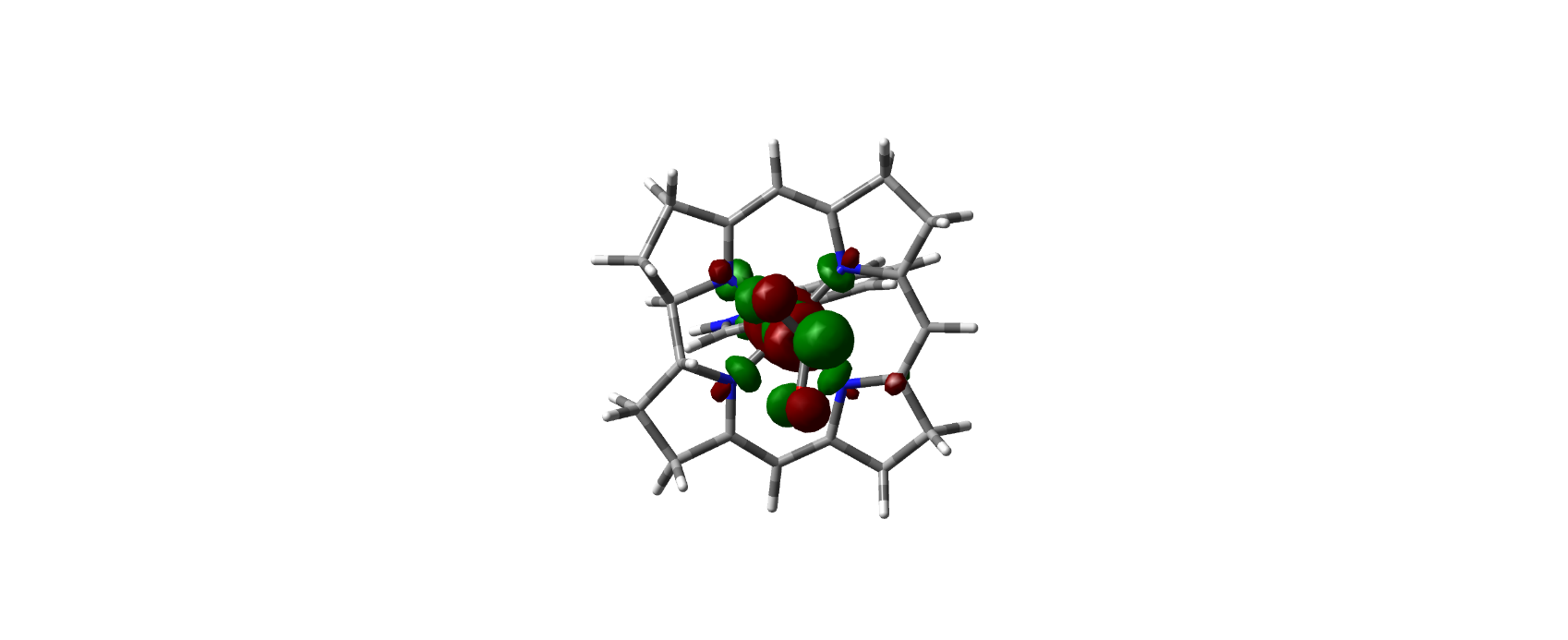


416 nm

HOMO-10 LUMO


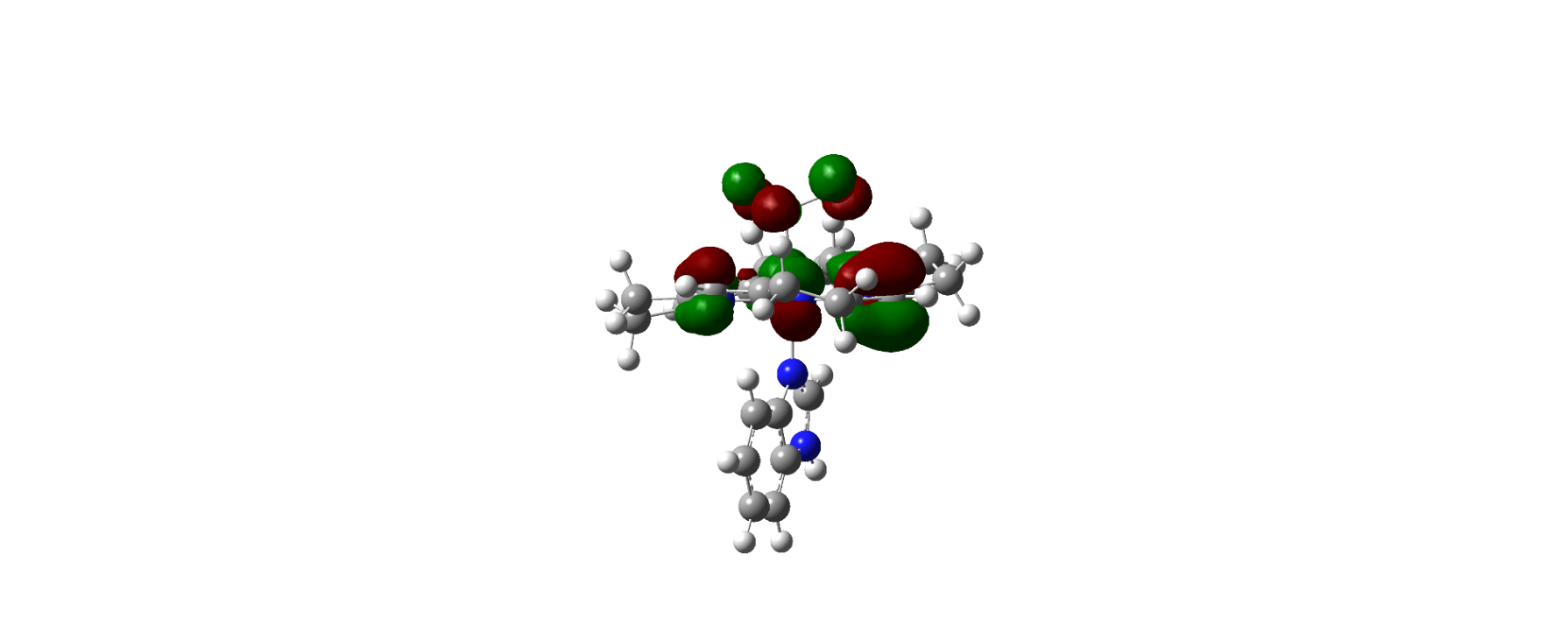

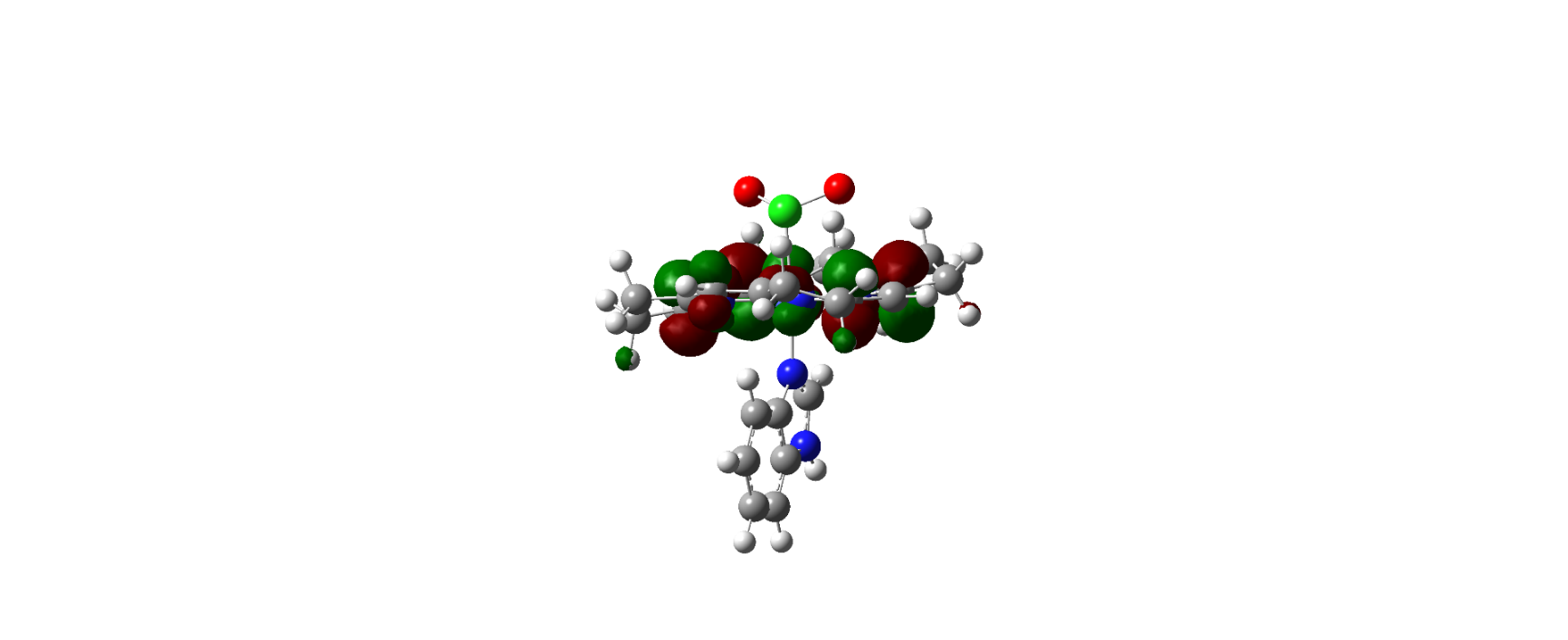


301 nm

HOMO-1 LUMO+2


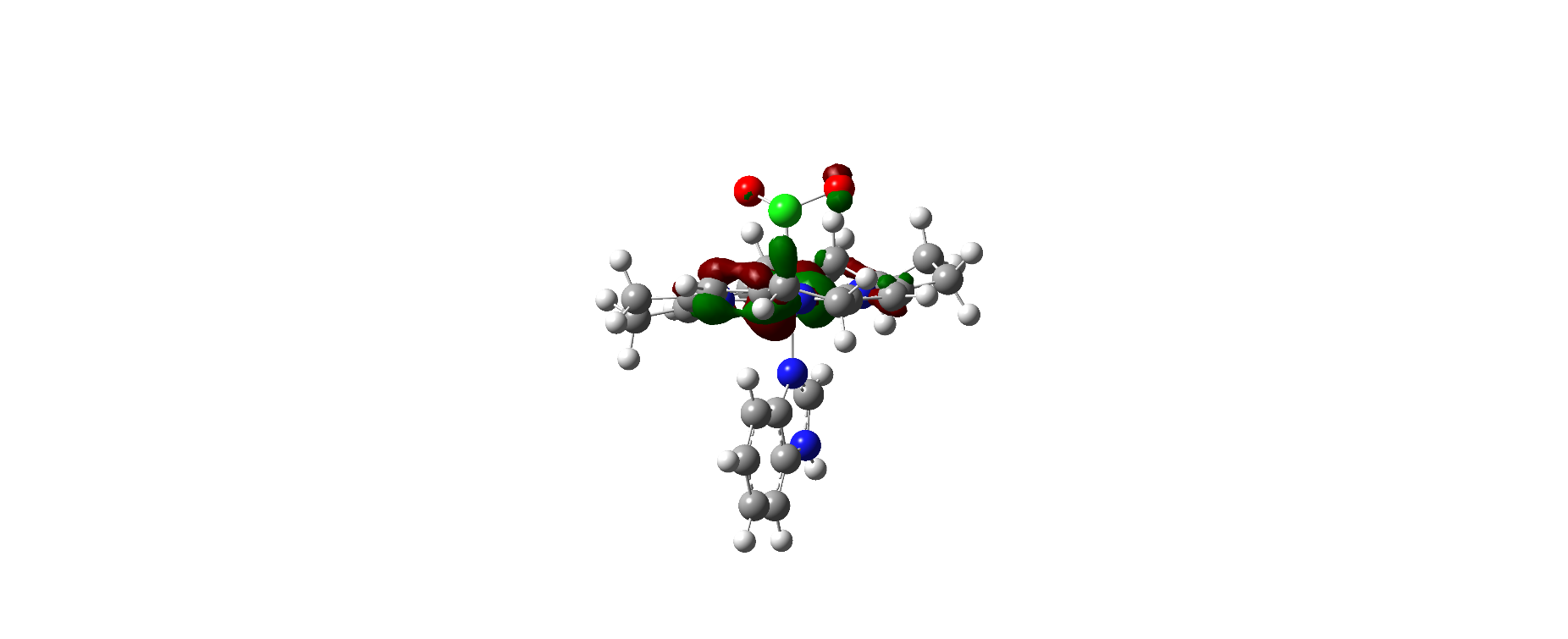

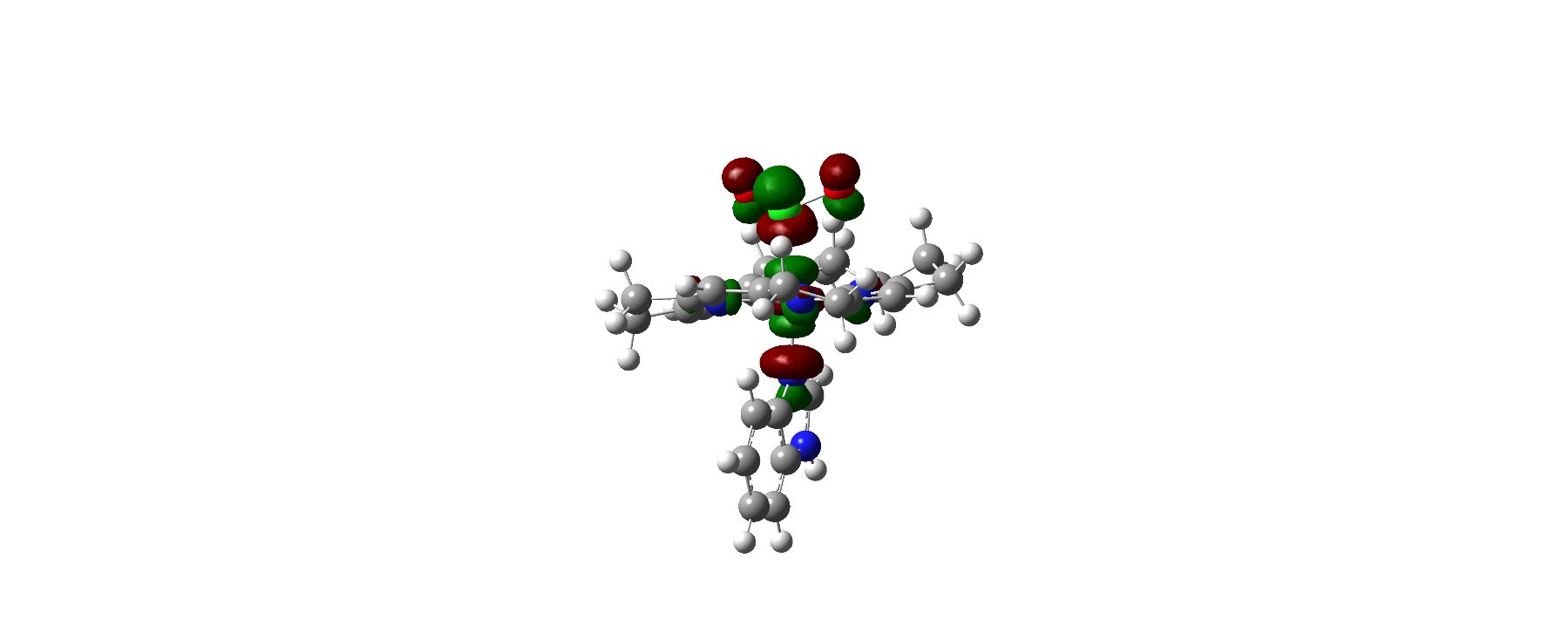


416 nm

HOMO-10 LUMO

**Figure S1.** The main orbitals responsible for the main contributors to the two main bands in the TD-DFT spectra in Co-Cl-(O)_2_ cf. B3PW91 calculations.


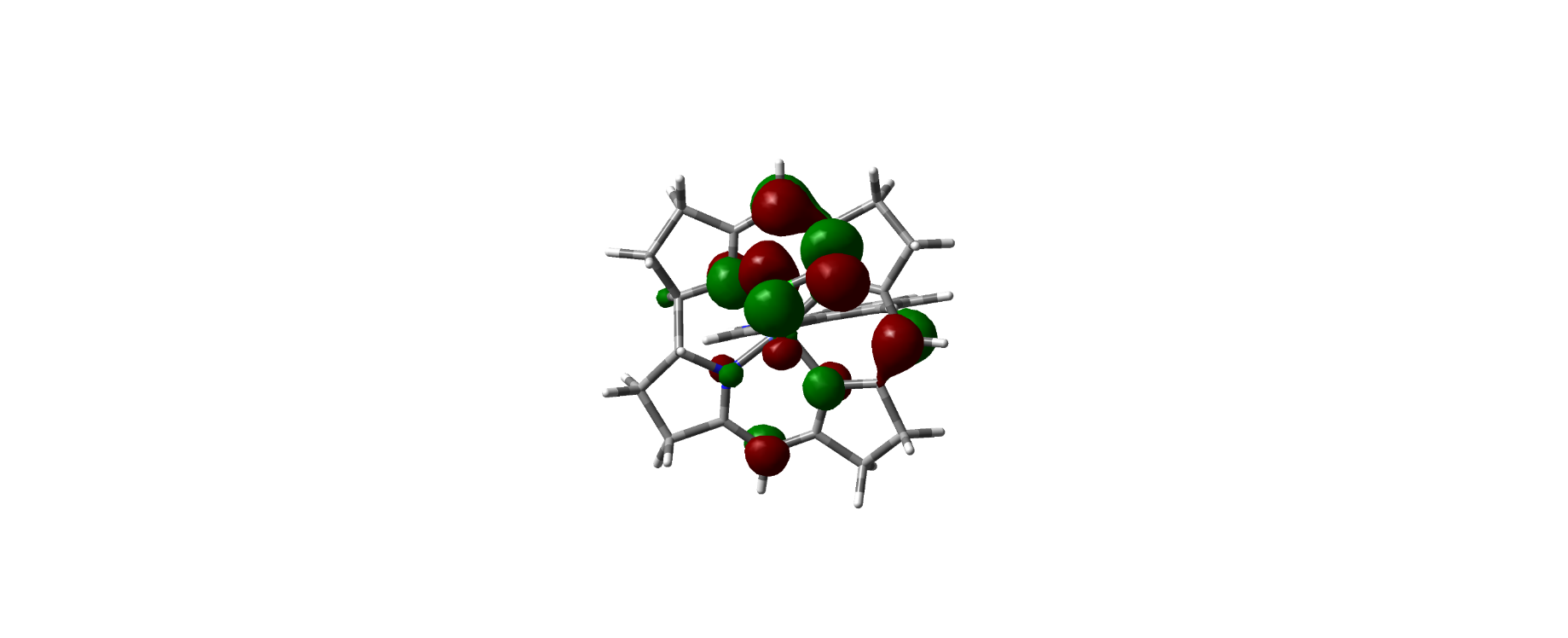

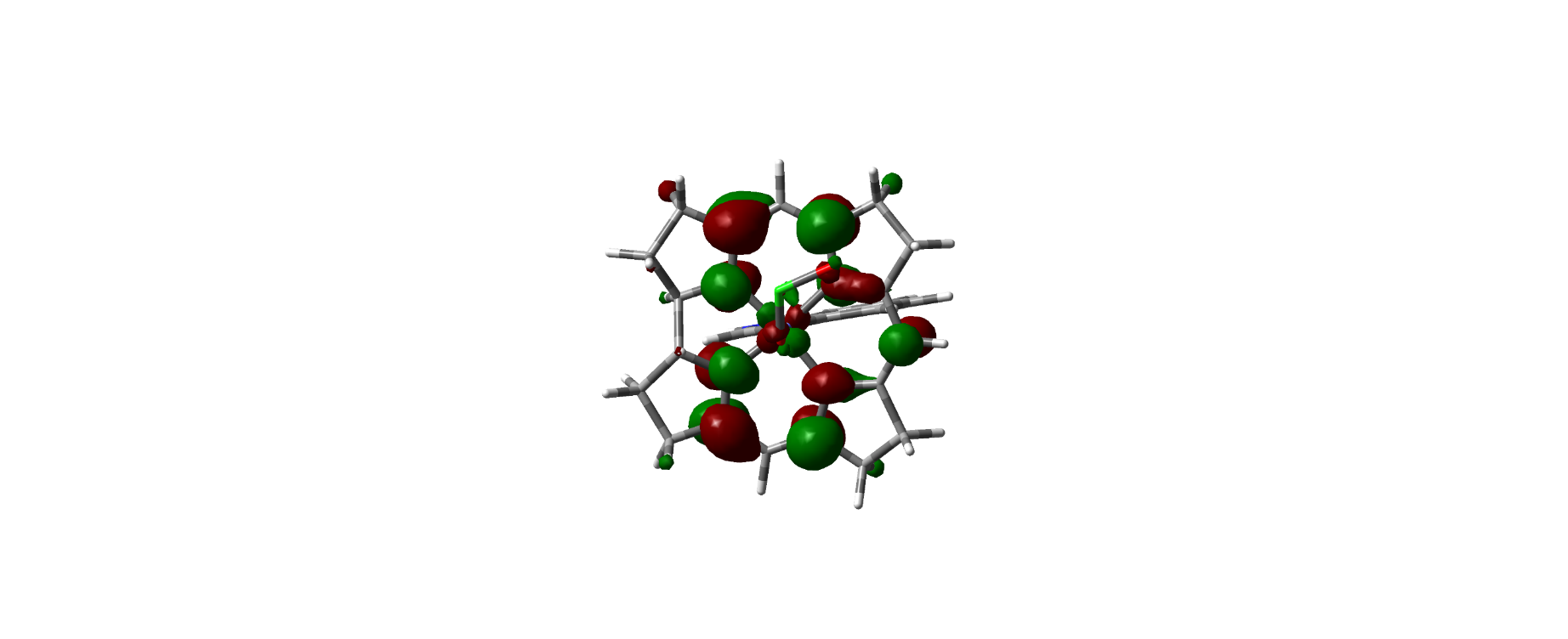


310 nm

HOMO-1 LUMO+2


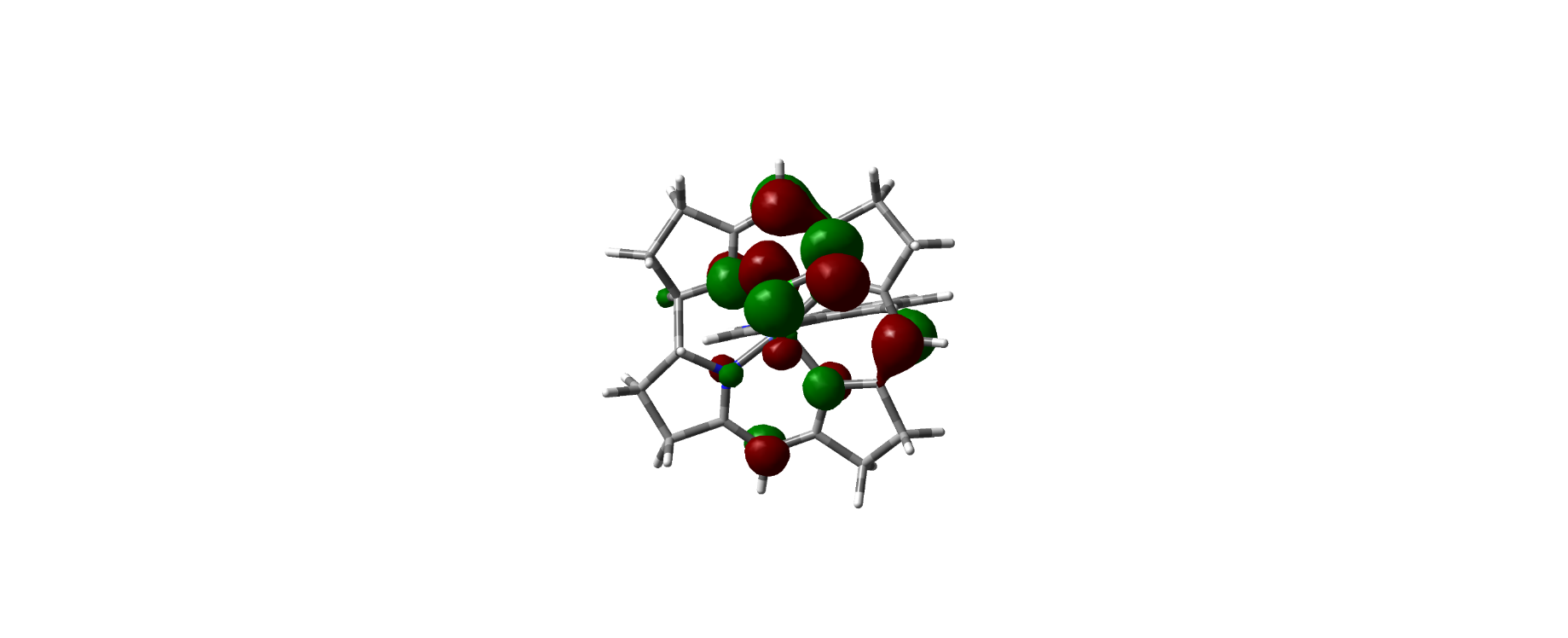

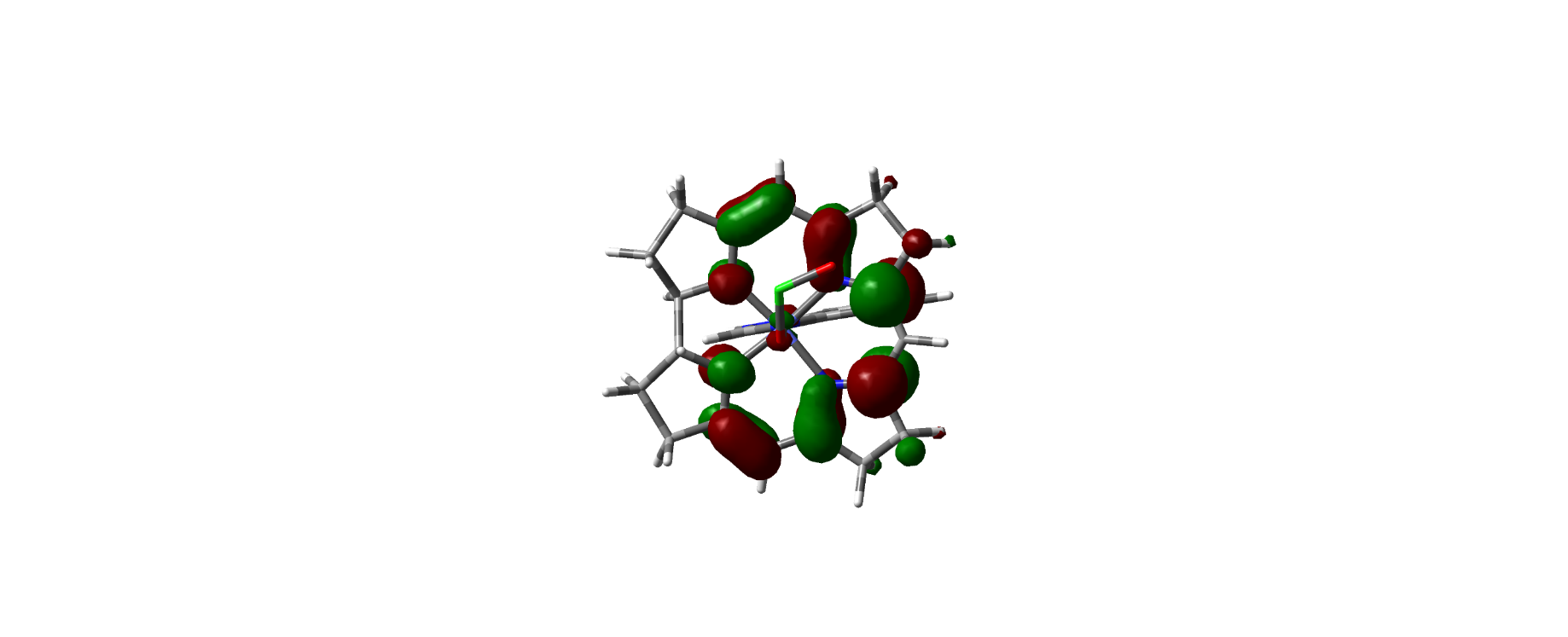


426 nm

HOMO-1 LUMO


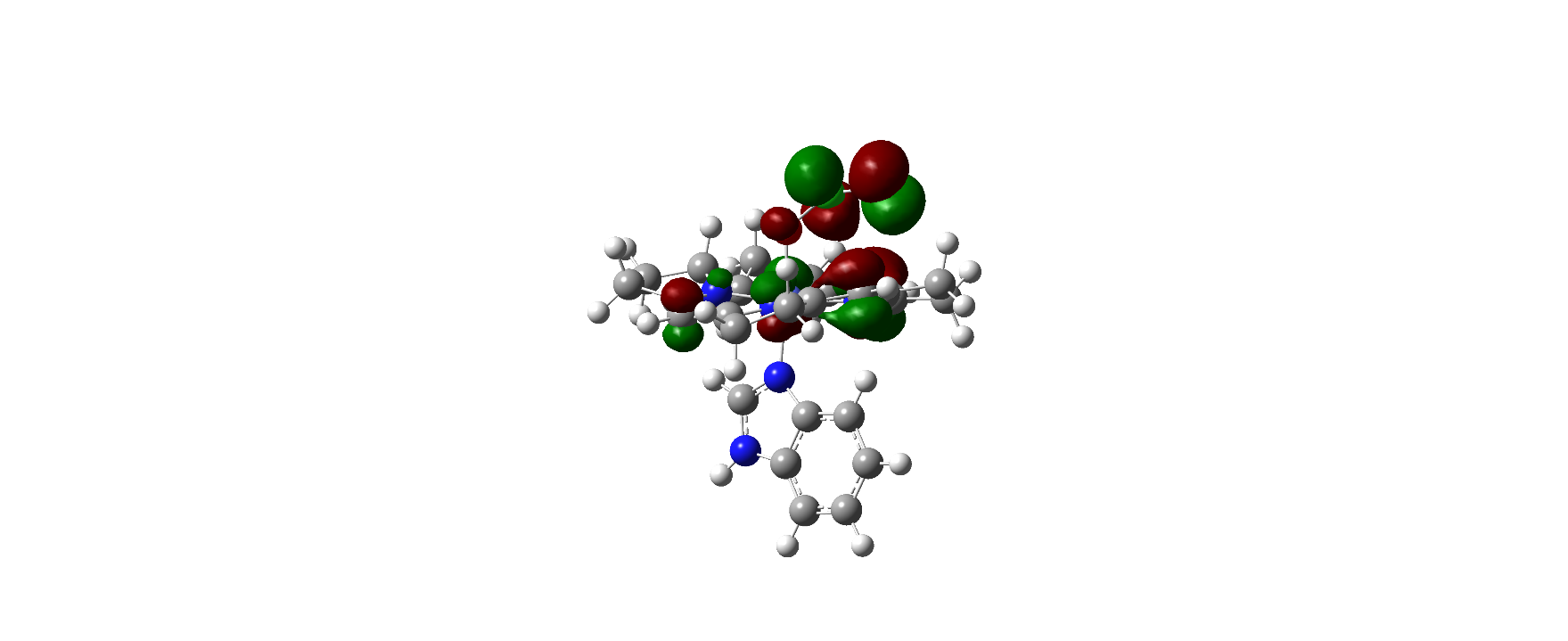

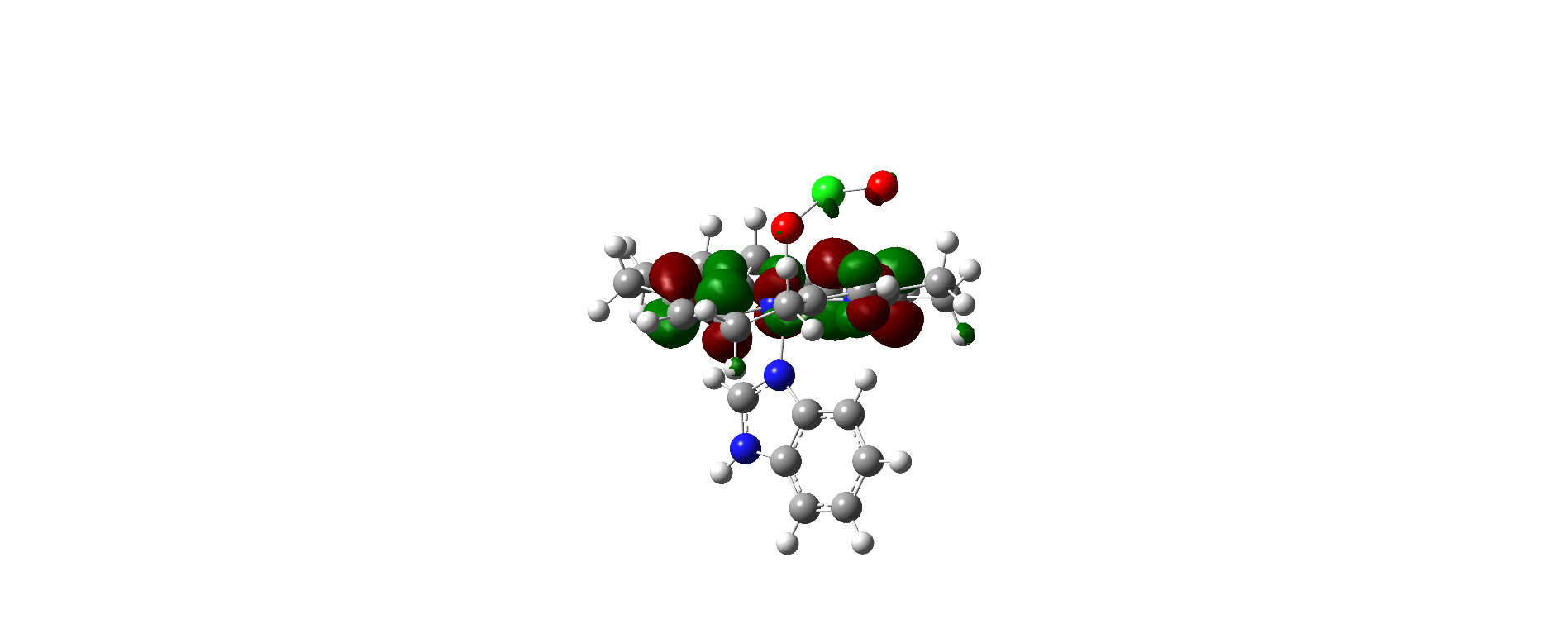


310 nm

HOMO-1 LUMO+2


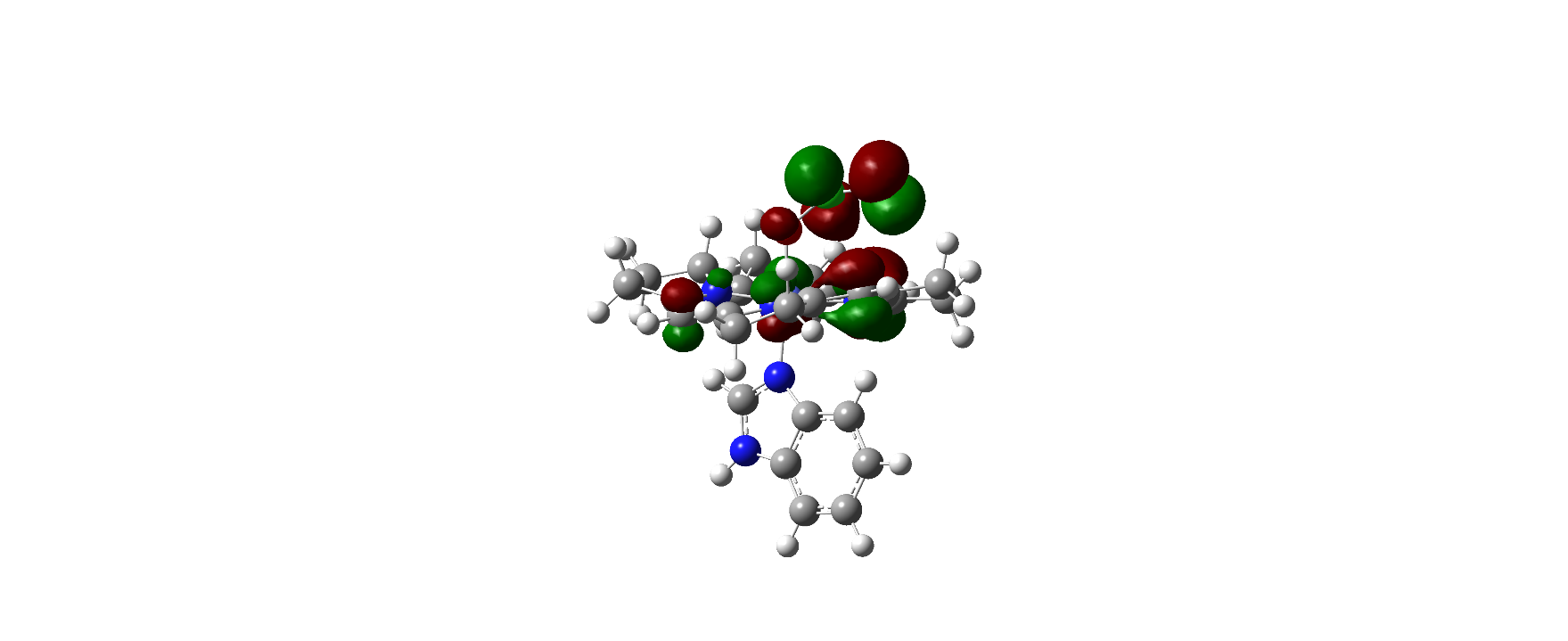

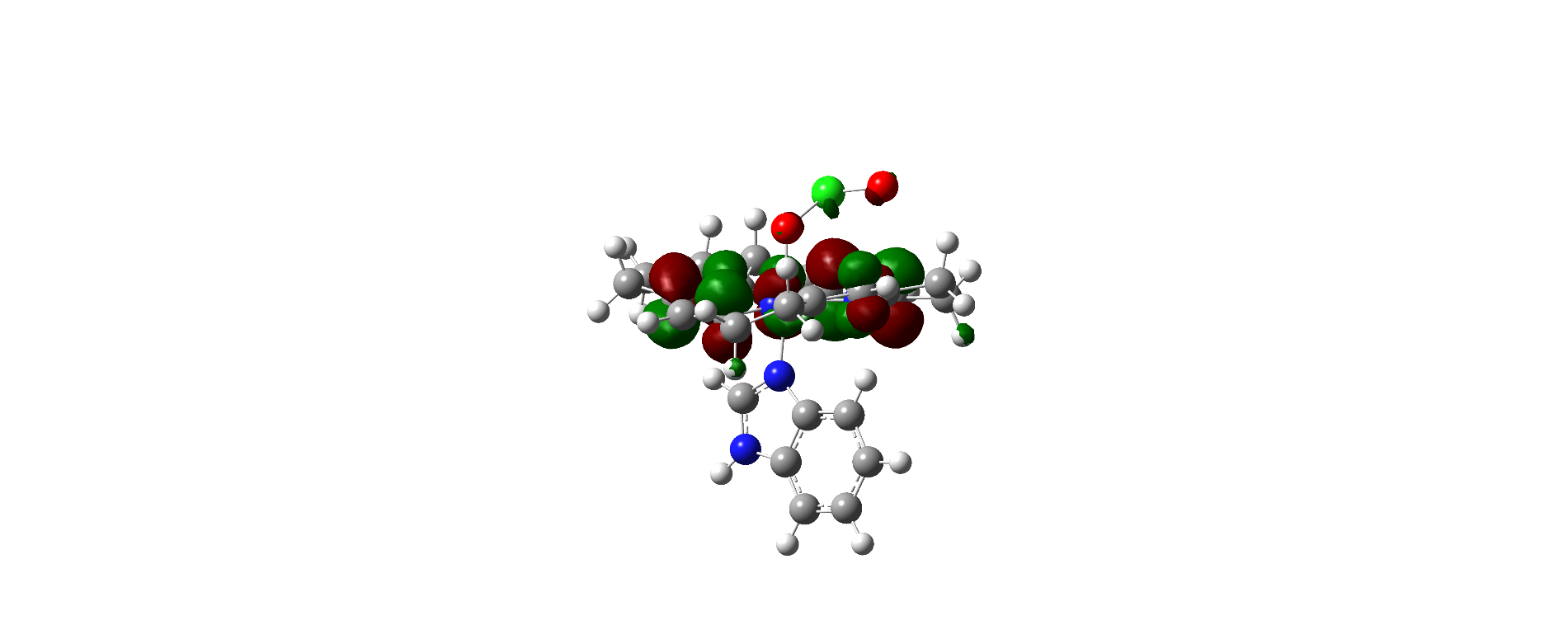


426 nm

HOMO-1 LUMO

**Figure S2.** The main orbitals responsible for the main contributors to the two main bands in the TD-DFT spectra in Co-O-Cl-O cf. B3PW91 calculations.

**Figure S3.** Details of resonance Raman spectra of Cbl in the presence or absence of chlorite. Conditions: 1 mM aqua-Cbl(III), 20 mM chlorite, 50 mM phosphate pH 7, 22°C. Shown for reference is also the control spectrum of 20 mM chlorite alone, without cobalamin.

**Figure S4.** Resonance Raman spectra of 1 mM aqua-Cbl(III) in presence or absence of 20 mM, 50 mM and 100 mM chlorite at pH 7, 22°C. Shown for reference is also the control spectrum of 20 mM chlorite alone, without cobalamin.

**Figure S5.** Raman spectra computed with DFT/B3LYP for aquaCbl complexed with water and chlorite.


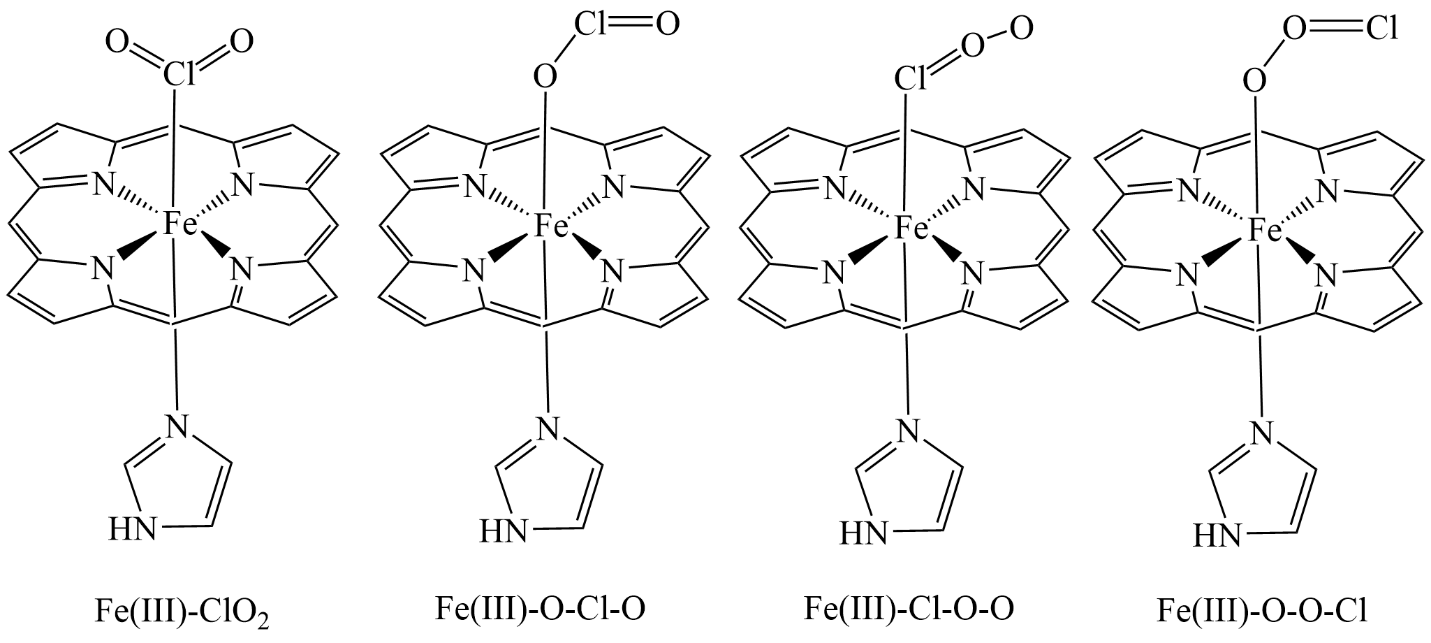


**Figure S6**. Models for chlorite adducts of chlorite dismutase.

**Figure S7.** Left: UV-vis spectra of the reaction between 100 μM aquaCbl and 0.5 mM-100 mM chlorite at pH 7, room temperature. Right: titration curve for aquacobalamin reacting with chlorite at pH 7, monitored at 430 nm and fitted to a simple hyperbolic equation (normalized ΔA = [chlorite]/(Kd + [chlorite]).

**Figure S8.** UV-vis spectra of aquaCbl reacting with 1 mM and 2 mM cyanide, compared to cyanoCbl.

**Table S1.** Detailed information for NMR peaks shown in Figure 2 for Cbl-OH_2_.

| **Proton (Cbl-OH_2_)** | **Integral** | **δ (ppm)** | **Multiplicity** |
| --- | --- | --- | --- |
| **R1** | 1.01 | 6.24 | d |
| **C10** | 1.03 | 6.28 | s |
| **B4** | 1.04 | 6.47 | s |
| **B2** | 1.06 | 6.55 | s |
| **B7** | 1.00 | 7.17 | s |
|  |  |  |  |

**Table S2.** Detailed information for NMR peaks shown in Figure 2 for Cbl-OH_2_ + 100 mM chlorite.

| **Proton (Cbl-OH_2_ + 100 mM chlorite)** | **Integral** | **δ (ppm)** | **Multiplicity** |
| --- | --- | --- | --- |
| **R1** | 1.02 | 6.24 | d |
| **C10** | 0.99 | 6.28 | s |
| **B4** | 1.05 | 6.42 | s |
| **B2** | 1.01 | 6.65 | s |
| **B7** | 1.00 | 7.16 | s |


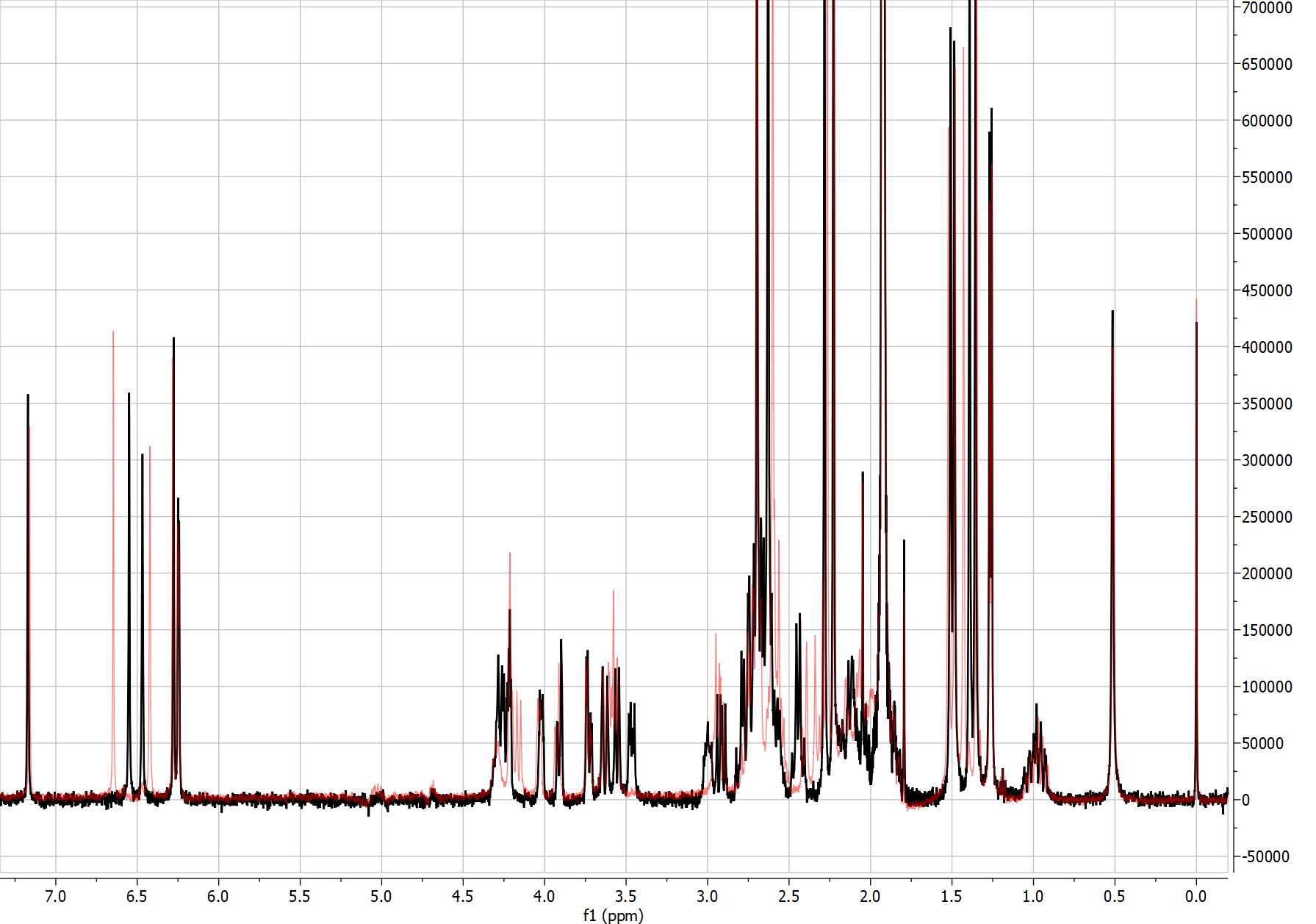


**Figure S9.** Complete ^1^H-NMR spectra of 1 mM aquaCbl (black) and aquaCbl + 100 mM chlorite (red) at pH 7.

Table S3. Vacuum. Relative energies and relative distances performed using the TPSS functional with a def2-SV(P) basis set.

| **Structure** | **S** | **Δ** | **d(Fe-L1)** | **d(L1-L2)** | **d(L2-L3)** |
| --- | --- | --- | --- | --- | --- |
|  |  | **kcal/mol** | **A** | **A** | **A** |
| Fe(III)-ClO_2_ | 1/2 | 68.8 | 2.33 | 1.55 | 1.55 |
| Fe(III)-ClO_2_ | 3/2 | 62.0 | 2.01 | 1.68 | 1.57 |
| Fe(III)-ClO_2_ | 5/2 | 87.2 | 3.02 | 1.56 | 1.56 |
| Fe(III)-O-Cl-O | 1/2 | 33.4 | 1.64 | 2.59 | 1.63 |
| Fe(III)-O-Cl-O | 3/2 | 62.0 | 2.01 | 1.68 | 1.57 |
| Fe(III)-O-Cl-O | 5/2 | 71.2 | 1.96 | 1.68 | 1.57 |
| Fe(III)-Cl-O-O | 1/2 | 0.00 | 2.22 | 3.24 | 1.22 |
| Fe(III)-Cl-O-O | 3/2 | 1.3 | 2.21 | 3.39 | 1.22 |
| Fe(III)-Cl-O-O | 5/2 | 26.7 | 2.25 | 3.44 | 1.22 |
| Fe(III)-O-O-Cl | 1/2 | 19.0 | 1.86 | 1.30 | 2.03 |
| Fe(III)-O-O-Cl | 3/2 | 36.2 | 2.15 | 1.26 | 2.20 |
| Fe(III)-O-O-Cl | 5/2 | 47.9 | 2.11 | 1.27 | 2.08 |

*For the Fe(III)-OClO_Cl isomer, Fe-L1 distance between Fe-Cl, L1-L2 distance between Cl-O(1) and L2-L3 distance between Cl-O(2). *For the Fe(III)-OClO_O isomer, Fe-L1 distance between Fe-O(1), L1-L2 distance between O(1)-Cl, L2-L3 distance between Cl-O(2). *For the Fe(III)-OOCl_Cl isomer, Fe-L1 distance between Fe-Cl, L1-L2 distance between Cl-O(1), L2-L3 distance between O(1)-O(2). *For the Fe(III)-OOCl_O isomer, Fe-L1 distance between Fe-O(1), L1-L2 distance between O(1)-O(2), L2-L3 distance between O(2)-Cl.

**Table S4.** Solvent. Relative energies and relative distances are performed using the TPSS functional with a def2-SV(P) basis set.

| **Structure** | **S** | **Δ** | **d(Fe-L1)*** | **d(L1-L2)*** | **d(L2-L3)*** |
| --- | --- | --- | --- | --- | --- |
|  |  | **kcal/mol** | **A** | **A** | **A** |
| Fe(III)-ClO_2_ | 1/2 | 67.1 | 2.38 | 1.57 | 1.57 |
| Fe(III)-ClO_2_ | 3/2 | 62.6 | 2.05 | 1.69 | 1.58 |
| Fe(III)-ClO_2_ | 5/2 | 87.6 | 2.92 | 1.57 | 1.57 |
| Fe(III)-O-Cl-O | 1/2 | 33.7 | 1.64 | 2.51 | 1.64 |
| Fe(III)-O-Cl-O | 3/2 | 62.6 | 2.05 | 1.69 | 1.58 |
| Fe(III)-O-Cl-O | 5/2 | 73.1 | 2.00 | 1.69 | 1.58 |
| Fe(III)-Cl-O-O | 1/2 | 0.00 | 2.27 | 3.38 | 1.22 |
| Fe(III)-Cl-O-O | 3/2 | 1.7 | 2.26 | 3.49 | 1.22 |
| Fe(III)-Cl-O-O | 5/2 | 11.2 | 2.37 | 3.41 | 1.22 |
| Fe(III)-O-O-Cl | 1/2 | 18.9 | 1.89 | 1.29 | 2.15 |
| Fe(III)-O-O-Cl | 3/2 | 22.7 | 1.96 | 1.26 | 2.64 |
| Fe(III)-O-O-Cl | 5/2 | 46.3 | 2.19 | 1.26 | 2.38 |

*For the Fe(III)-OClO_Cl isomer, Fe-L1 distance between Fe-Cl, L1-L2 distance between Cl-O(1) and L2-L3 distance between Cl-O(2). *For the Fe(III)-OClO_O isomer, Fe-L1 distance between Fe-O(1), L1-L2 distance between O(1)-Cl, L2-L3 distance between Cl-O(2). *For the Fe(III)-OOCl_Cl isomer, Fe-L1 distance between Fe-Cl, L1-L2 distance between Cl-O(1), L2-L3 distance between O(1)-O(2). *For the Fe(III)-OOCl_O isomer, Fe-L1 distance between Fe-O(1), L1-L2 distance between O(1)-O(2), L2-L3 distance between O(2)-Cl.

**Table S5.** Partial charge derived from the Mulliken population of the optimized TPSS structures with a def2-SV(P) basis set in vacuum and solvent.

| **Isomer** | **S** | **Partial charges** | | | | | |
| --- | --- | --- | --- | --- | --- | --- | --- |
|  |  | **Fe** | **O(1)** | **O(2)** | **Cl** | **∑ charges O_2_** | **∑ charges ClO_2_** |
| Fe(III)-ClO_2_ | 1/2 | 0.51 | -0.43 | -0.44 | 0.66 | -0.87  *-1.01* | -0.21 |
| *Fe(III)-ClO_2_* | *1/2* | *0.51* | *-0.50* | *-0.50* | *0.66* |  | *-0.35* |
| Fe(III)-ClO_2_ | 3/2 | 0.71 | -0.47 | -0.47 | 0.55 | -0.94 | -0.38 |
| *Fe(III)-ClO_2_* | *3/2* | *0.69* | *-0.51* | *-0.52* | *0.55* | *-1.03* | *-0.48* |
| Fe(III)-ClO_2_ | 5/2 | 0.78 | -0.41 | -0.41 | 0.66 | -0.83 | -0.17 |
| *Fe(III)-ClO_2_* | *5/2* | *0.79* | *-0.47* | *-0.47* | *0.66* | *-0.95* | *-0.29* |
| Fe(III)-O-Cl-O | 1/2 | 0.55 | -0.31 | -0.33 | 0.23 | -0.64 | -0.41 |
| *Fe(III)-O-Cl-O* | *1/2* | *0.55* | *-0.31* | *-0.34* | *0.23* | *-0.65* | *-0.42* |
| Fe(III)-O-Cl-O | 3/2 | 0.71 | -0.47 | -0.47 | 0.55 | -0.94 | -0.38 |
| *Fe(III)-O-Cl-O* | *3/2* | *0.69* | *-0.51* | *-0.52* | *0.55* | *-1.03* | *-0.48* |
| Fe(III)-O-Cl-O | 5/2 | 0.83 | -0.47 | -0.45 | 0.57 | -0.92 | -0.35 |
| *Fe(III)-O-Cl-O* | *5/2* | *0.81* | *-0.50* | *-0.50* | *0.58* | *-1.00* | *-0.42* |
| Fe(III)-Cl-O-O | 1/2 | 0.47 | 0.01 | 0.00 | -0.29 | 0.01 | -0.28 |
| *Fe(III)-Cl-O-O* | *1/2* | *0.48* | *0.00* | *0.00* | *-0.38* | *0.00* | *-0.38* |
| Fe(III)-Cl-O-O | 3/2 | 0.46 | 0.00 | 0.01 | -0.27 | 0.01 | -0.27 |
| *Fe(III)-Cl-O-O* | *3/2* | *0.46* | *0.00* | *0.00* | *-0.37* | *0.00* | *-0.37* |
| Fe(III)-Cl-O-O | 5/2 | 0.75 | 0.00 | 0.00 | -0.31 | 0.00 | -0.31 |
| *Fe(III)-Cl-O-O* | *5/2* | *0.75* | *0.00* | *0.00* | *-0.40* | *-0.01* | *-0.40* |
| Fe(III)-O-O-Cl | 1/2 | 0.54 | -0.16 | -0.10 | -0.08 | -0.25 | -0.33 |
| *Fe(III)-O-O-Cl* | *1/2* | *0.53* | *-0.19* | *-0.12* | *-0.12* | *-0.30* | *-0.42* |
| Fe(III)-O-O-Cl | 3/2 | 0.70 | -0.18 | -0.08 | -0.17 | -0.26 | -0.44 |
| *Fe(III)-O-O-Cl* | *3/2* | *0.64* | *-0.11* | *0.01* | *-0.64* | *-0.10* | *-0.74* |
| Fe(III)-O-O-Cl | 5/2 | 0.83 | -0.20 | -0.09 | -0.09 | -0.29 | -0.38 |
| *Fe(III)-O-O-Cl* | *5/2* | *0.82* | *-0.24* | *-0.11* | *-0.13* | *-0.35* | *-0.48* |

*in italics: calculations performed in solvent

**Table S6.** Partial charge derived from the Mulliken population of the optimized TPSS structures with a def2-SV(P) basis set in vacuum and solvent.

| **Isomer** | **S** | **Partial spin densities** | | | | | |
| --- | --- | --- | --- | --- | --- | --- | --- |
|  |  | **Fe** | **O(1)** | **O(2)** | **Cl** | **∑O_2_** | **∑ClO_2_** |
| Fe(III)-ClO_2_ | 1/2 | 1.51 | -0.16 | -0.16 | -0.13 | -0.33 | -0.46 |
| *Fe(III)-ClO_2_* | *1/2* | *1.34* | *-0.10* | *-0.10* | *-0.09* | *-0.20* | *-0.29* |
| Fe(III)-ClO_2_ | 3/2 | 2.66 | 0.22 | 0.09 | 0.06 | 0.31 | 0.37 |
| *Fe(III)-ClO_2_* | *3/2* | *2.72* | *0.18* | *0.06* | *0.04* | *0.24* | *0.28* |
| Fe(III)-ClO_2_ | 5/2 | 3.95 | 0.27 | 0.27 | 0.21 | 0.54 | 0.75 |
| *Fe(III)-ClO_2_* | *5/2* | *4.00* | *0.22* | *0.22* | *0.18* | *0.44* | *0.62* |
| Fe(III)-O-Cl-O | 1/2 | 1.17 | 0.78 | -0.62 | -0.22 | 0.16 | 0.06 |
| *Fe(III)-O-Cl-O* | *1/2* | *1.18* | *0.77* | *-0.64* | *-0.22* | *0.13* | *0.09* |
| Fe(III)-O-Cl-O | 3/2 | 2.66 | 0.22 | 0.09 | 0.06 | 0.31 | 0.37 |
| *Fe(III)-O-Cl-O* | *3/2* | *2.72* | *0.18* | *0.06* | *0.04* | *0.24* | *0.28* |
| Fe(III)-O-Cl-O | 5/2 | 4.16 | 0.23 | 0.10 | 0.06 | 0.33 | 0.40 |
| *Fe(III)-O-Cl-O* | *5/2* | *4.18* | *0.20* | *0.07* | *0.05* | *0.27* | *0.32* |
| Fe(III)-Cl-O-O | 1/2 | -1.01 | 1.00 | 0.99 | -0.06 | 1.98 | 1.92 |
| *Fe(III)-Cl-O-O* | *1/2* | *-1.04* | *0.99* | *0.99* | *-0.03* | *1.98* | *1.95* |
| Fe(III)-Cl-O-O | 3/2 | 0.97 | 0.99 | 1.00 | 0.09 | 1.99 | 2.08 |
| *Fe(III)-Cl-O-O* | *3/2* | *1.00* | *0.99* | *0.99* | *0.05* | *1.98* | *2.04* |
| Fe(III)-Cl-O-O | 5/2 | 4.18 | 0.00 | 0.00 | 0.34 | 0.00 | 0.34 |
| *Fe(III)-Cl-O-O* | *5/2* | *4.20* | *0.00* | *0.00* | *0.27* | *0.00* | *0.27* |
| Fe(III)-O-O-Cl | 1/2 | 1.02 | -0.01 | -0.03 | 0.07 | -0.05 | 0.03 |
| *Fe(III)-O-O-Cl* | *1/2* | *1.02* | *-0.02* | *-0.03* | *0.07* | *-0.05* | *0.02* |
| Fe(III)-O-O-Cl | 3/2 | 2.64 | 0.24 | 0.09 | 0.02 | 0.33 | 0.35 |
| *Fe(III)-O-O-Cl* | *3/2* | *1.16* | *0.61* | *0.91* | *0.31* | *1.52* | *1.83* |
| Fe(III)-O-O-Cl | 5/2 | 4.16 | 0.25 | 0.09 | 0.03 | 0.35 | 0.38 |
| *Fe(III)-O-O-Cl* | *5/2* | *4.19* | *0.20* | *0.06* | *0.02* | *0.26* | *0.28* |

*in italics: calculations in solvent; *∑O_2_ = sum of spin density on O_2_; _*_∑ ClO_2_ =sum of spin density on ClO_2_

**A**

**B**

**Figure S7**. **A:** Difference UV-vis spectra of the reaction between 50 μM aquaCbl and 0.5 mM-100 mM chlorite at pH 7, room temperature. **B:** UV-vis spectra of 100 mM chlorite at pH 7, room temperature.

DFT-optimized coordinates of the chlorite dismutase models:

Fe(III)-ClO_2__sm2

Standard orientation:

---------------------------------------------------------------------

Center Atomic Atomic Coordinates (Angstrom)

Number Number Type X Y Z

---------------------------------------------------------------------

1 6 0 3.420363 -0.036251 -0.399859

2 6 0 0.012662 3.345778 0.316104

3 6 0 -3.432511 -0.007281 -0.349336

4 6 0 -0.019305 -3.457367 -0.111981

5 6 0 2.782379 1.189683 -0.216980

6 6 0 3.470336 2.457148 -0.118592

7 6 0 2.513704 3.401728 0.140164

8 6 0 1.243875 2.709249 0.170652

9 6 0 -1.225423 2.718743 0.191272

10 6 0 -2.490084 3.421779 0.181347

11 6 0 -3.457839 2.485381 -0.063433

12 6 0 -2.781623 1.212444 -0.173028

13 6 0 -2.795087 -1.244749 -0.339279

14 6 0 -3.481025 -2.514303 -0.396078

15 6 0 -2.522901 -3.485672 -0.270124

16 6 0 -1.251221 -2.806966 -0.164648

17 6 0 1.217654 -2.817981 -0.183138

18 6 0 2.481690 -3.507357 -0.306140

19 6 0 3.446623 -2.544096 -0.445620

20 6 0 2.772526 -1.268709 -0.380380

21 7 0 1.423093 1.356497 -0.035916

22 7 0 -1.418820 1.367936 -0.013027

23 7 0 -1.429172 -1.435583 -0.201016

24 7 0 1.406879 -1.447934 -0.222999

25 26 0 -0.005495 -0.043778 -0.074873

26 1 0 4.505326 -0.032132 -0.536400

27 1 0 0.018047 4.428930 0.468682

28 1 0 -4.519294 0.006202 -0.469209

29 1 0 -0.024066 -4.550988 -0.089403

30 1 0 4.547541 2.587975 -0.234840

31 1 0 2.635066 4.478117 0.274401

32 1 0 -2.600617 4.498779 0.319664

33 1 0 -4.535750 2.624483 -0.161513

34 1 0 -4.560027 -2.630341 -0.511687

35 1 0 -2.647494 -4.570106 -0.269210

36 1 0 2.596984 -4.592855 -0.307676

37 1 0 4.522740 -2.669735 -0.577533

38 7 0 0.741528 -0.256997 3.958846

39 6 0 1.099431 -0.161436 2.651679

40 7 0 0.006962 -0.117965 1.895091

41 6 0 -1.087557 -0.185671 2.734570

42 6 0 -0.643114 -0.273926 4.034709

43 1 0 2.124143 -0.124941 2.283688

44 1 0 -2.101539 -0.165073 2.338884

45 1 0 -1.171372 -0.344710 4.983540

46 1 0 1.385929 -0.306453 4.744837

47 17 0 -0.022532 0.128947 -2.397030

48 8 0 -1.350822 0.769871 -2.878464

49 8 0 1.300281 0.763414 -2.901305

---------------------------------------------------------------------

Fe(III)-ClO_2__sm2_solv

Standard orientation:

---------------------------------------------------------------------

Center Atomic Atomic Coordinates (Angstrom)

Number Number Type X Y Z

---------------------------------------------------------------------

1 6 0 3.433270 -0.001404 -0.364026

2 6 0 -0.010925 3.375771 0.227230

3 6 0 -3.440374 -0.021302 -0.335326

4 6 0 0.007538 -3.438263 -0.066197

5 6 0 2.781049 1.220984 -0.217702

6 6 0 3.458640 2.494133 -0.135294

7 6 0 2.490794 3.440128 0.080897

8 6 0 1.224521 2.741833 0.105836

9 6 0 -1.243550 2.734663 0.114302

10 6 0 -2.513851 3.425820 0.098017

11 6 0 -3.477968 2.474115 -0.110011

12 6 0 -2.793910 1.204797 -0.195719

13 6 0 -2.790574 -1.252590 -0.309189

14 6 0 -3.465263 -2.528569 -0.335667

15 6 0 -2.495281 -3.489278 -0.206219

16 6 0 -1.229189 -2.796993 -0.126394

17 6 0 1.240020 -2.790239 -0.138825

18 6 0 2.509248 -3.475076 -0.231330

19 6 0 3.472443 -2.508697 -0.369018

20 6 0 2.790712 -1.236594 -0.335236

21 7 0 1.414422 1.384811 -0.071206

22 7 0 -1.427153 1.376610 -0.060221

23 7 0 -1.421498 -1.428009 -0.185551

24 7 0 1.423809 -1.419969 -0.198930

25 26 0 -0.003392 -0.020050 -0.057582

26 1 0 4.521402 0.007521 -0.468412

27 1 0 -0.013608 4.460815 0.362583

28 1 0 -4.529352 -0.018695 -0.430916

29 1 0 0.010714 -4.530790 -0.022296

30 1 0 4.538089 2.627186 -0.224332

31 1 0 2.603907 4.519145 0.199939

32 1 0 -2.632202 4.504289 0.216899

33 1 0 -4.558777 2.601013 -0.191135

34 1 0 -4.544931 -2.655767 -0.430365

35 1 0 -2.608734 -4.574488 -0.179843

36 1 0 2.629266 -4.559626 -0.206941

37 1 0 4.551909 -2.629565 -0.473860

38 7 0 0.712676 -0.185949 3.992971

39 6 0 1.084598 -0.122943 2.694685

40 7 0 -0.003103 -0.083769 1.922393

41 6 0 -1.107047 -0.122576 2.755487

42 6 0 -0.669898 -0.186973 4.059535

43 1 0 2.115873 -0.107240 2.345888

44 1 0 -2.120132 -0.101446 2.357873

45 1 0 -1.202561 -0.232257 5.007241

46 1 0 1.351287 -0.226398 4.787339

47 17 0 -0.011215 -0.046374 -2.442225

48 8 0 -1.337077 0.611629 -2.956626

49 8 0 1.304902 0.622798 -2.967116

---------------------------------------------------------------------

Fe(III)-ClO_2__sm4

Standard orientation:

---------------------------------------------------------------------

Center Atomic Atomic Coordinates (Angstrom)

Number Number Type X Y Z

---------------------------------------------------------------------

1 6 0 2.827627 -1.411897 0.807506

2 6 0 1.308759 3.144946 0.046050

3 6 0 -3.105282 1.475605 -1.140245

4 6 0 -1.557946 -3.088853 -0.449455

5 6 0 2.791131 -0.021862 0.703630

6 6 0 3.923736 0.839790 0.949839

7 6 0 3.497900 2.124283 0.736566

8 6 0 2.106651 2.043408 0.357127

9 6 0 -0.030978 3.079038 -0.337612

10 6 0 -0.847331 4.235075 -0.635677

11 6 0 -2.092271 3.765156 -0.962017

12 6 0 -2.032303 2.323149 -0.867562

13 6 0 -3.062160 0.081073 -1.070938

14 6 0 -4.177860 -0.787280 -1.362712

15 6 0 -3.737305 -2.074270 -1.175074

16 6 0 -2.355863 -1.986214 -0.764618

17 6 0 -0.228915 -3.025826 -0.030884

18 6 0 0.582485 -4.182114 0.287070

19 6 0 1.818494 -3.709971 0.642339

20 6 0 1.756852 -2.266782 0.540095

21 7 0 1.683607 0.729913 0.360765

22 7 0 -0.768437 1.921148 -0.482765

23 7 0 -1.959436 -0.664066 -0.706202

24 7 0 0.501441 -1.869524 0.137691

25 26 0 -0.110388 0.030513 -0.258218

26 1 0 3.777956 -1.869000 1.096905

27 1 0 1.771175 4.134713 0.097372

28 1 0 -4.050229 1.937391 -1.440565

29 1 0 -2.012009 -4.079881 -0.540232

30 1 0 4.918593 0.488970 1.228842

31 1 0 4.068829 3.051811 0.805584

32 1 0 -0.496656 5.267928 -0.597078

33 1 0 -2.980655 4.330742 -1.248948

34 1 0 -5.166188 -0.445067 -1.675080

35 1 0 -4.289472 -3.007515 -1.300017

36 1 0 0.236730 -5.216200 0.235631

37 1 0 2.703572 -4.273697 0.942734

38 7 0 -0.763063 0.497293 4.175115

39 6 0 -0.069590 0.569011 3.001712

40 7 0 -0.804195 0.104610 2.003015

41 6 0 -2.012297 -0.281061 2.542088

42 6 0 -2.008832 -0.043954 3.901782

43 1 0 0.945622 0.956016 2.912063

44 1 0 -2.798766 -0.700472 1.915075

45 1 0 -2.753869 -0.204177 4.679630

46 1 0 -0.424588 0.790807 5.089133

47 17 0 2.042796 -0.701113 -2.571423

48 8 0 0.484575 -0.233025 -2.158349

49 8 0 3.227284 0.248639 -2.154224

---------------------------------------------------------------------

Fe(III)-ClO_2__sm4_solv

Standard orientation:

---------------------------------------------------------------------

Center Atomic Atomic Coordinates (Angstrom)

Number Number Type X Y Z

---------------------------------------------------------------------

1 6 0 2.842731 -1.392045 0.848876

2 6 0 1.303983 3.153125 0.022160

3 6 0 -3.115838 1.454541 -1.116810

4 6 0 -1.522013 -3.101776 -0.454097

5 6 0 2.796106 -0.003151 0.730852

6 6 0 3.912093 0.871108 1.006830

7 6 0 3.481828 2.151447 0.768056

8 6 0 2.103287 2.056010 0.348086

9 6 0 -0.036032 3.079673 -0.361677

10 6 0 -0.861272 4.228675 -0.658265

11 6 0 -2.109657 3.750547 -0.966618

12 6 0 -2.042604 2.310107 -0.864579

13 6 0 -3.058923 0.060648 -1.045310

14 6 0 -4.170351 -0.817409 -1.328413

15 6 0 -3.715864 -2.101223 -1.152951

16 6 0 -2.330309 -2.003606 -0.756405

17 6 0 -0.192214 -3.029226 -0.034870

18 6 0 0.623581 -4.178583 0.292265

19 6 0 1.851550 -3.696631 0.668818

20 6 0 1.781922 -2.255363 0.565145

21 7 0 1.694245 0.736112 0.340837

22 7 0 -0.769904 1.915711 -0.494905

23 7 0 -1.943676 -0.676870 -0.696783

24 7 0 0.529554 -1.866666 0.140512

25 26 0 -0.104430 0.028844 -0.236719

26 1 0 3.785507 -1.839650 1.174683

27 1 0 1.759086 4.145510 0.081102

28 1 0 -4.068755 1.908369 -1.402137

29 1 0 -1.969000 -4.095461 -0.545791

30 1 0 4.895750 0.532635 1.336670

31 1 0 4.037157 3.086244 0.861401

32 1 0 -0.516218 5.263488 -0.625978

33 1 0 -3.005072 4.310048 -1.242722

34 1 0 -5.165551 -0.481314 -1.624440

35 1 0 -4.260864 -3.039068 -1.272963

36 1 0 0.285011 -5.214686 0.237831

37 1 0 2.734954 -4.253131 0.986482

38 7 0 -0.769717 0.511075 4.171972

39 6 0 -0.067315 0.594200 3.010190

40 7 0 -0.788892 0.121318 1.999043

41 6 0 -1.998465 -0.280268 2.530478

42 6 0 -2.004122 -0.043264 3.890825

43 1 0 0.943286 0.995310 2.939273

44 1 0 -2.778645 -0.709161 1.902028

45 1 0 -2.751221 -0.212766 4.664357

46 1 0 -0.441513 0.808036 5.090664

47 17 0 2.000801 -0.756739 -2.543985

48 8 0 0.440008 -0.218080 -2.197828

49 8 0 3.150461 0.328110 -2.431952

---------------------------------------------------------------------

Fe(III)-ClO_2__sm6

Standard orientation:

---------------------------------------------------------------------

Center Atomic Atomic Coordinates (Angstrom)

Number Number Type X Y Z

---------------------------------------------------------------------

1 6 0 2.244408 -2.430952 0.350785

2 6 0 2.244761 2.430637 0.350796

3 6 0 -2.509164 2.437097 -0.803988

4 6 0 -2.509516 -2.436727 -0.804006

5 6 0 2.681758 -1.105383 0.499885

6 6 0 4.054653 -0.686274 0.724157

7 6 0 4.054754 0.685693 0.724159

8 6 0 2.681920 1.105004 0.499888

9 6 0 0.949904 2.867111 0.023798

10 6 0 0.557223 4.250282 -0.124784

11 6 0 -0.779861 4.255433 -0.448304

12 6 0 -1.205137 2.873963 -0.500281

13 6 0 -2.952630 1.110321 -0.883997

14 6 0 -4.287974 0.686458 -1.264417

15 6 0 -4.288074 -0.685827 -1.264423

16 6 0 -2.952790 -1.109887 -0.884007

17 6 0 -1.205552 -2.873783 -0.500299

18 6 0 -0.780476 -4.255314 -0.448320

19 6 0 0.556609 -4.250356 -0.124798

20 6 0 0.949489 -2.867241 0.023784

21 7 0 1.869059 -0.000130 0.404685

22 7 0 -0.137609 2.050486 -0.214911

23 7 0 -2.166142 0.000159 -0.639964

24 7 0 -0.137905 -2.050460 -0.214927

25 26 0 -0.216744 0.000015 0.063815

26 1 0 3.003064 -3.211618 0.464977

27 1 0 3.003529 3.211194 0.464988

28 1 0 -3.246735 3.216326 -1.021042

29 1 0 -3.247199 -3.215850 -1.021065

30 1 0 4.906133 -1.361143 0.830049

31 1 0 4.906332 1.360437 0.830053

32 1 0 1.224083 5.105882 -0.002388

33 1 0 -1.422933 5.116043 -0.642654

34 1 0 -5.109776 1.360341 -1.514768

35 1 0 -5.109973 -1.359589 -1.514781

36 1 0 -1.423671 -5.115831 -0.642670

37 1 0 1.223345 -5.106052 -0.002399

38 7 0 -0.329700 0.000102 4.334498

39 6 0 0.296131 0.000114 3.127444

40 7 0 -0.604589 0.000018 2.151751

41 6 0 -1.850880 -0.000059 2.746082

42 6 0 -1.698743 -0.000010 4.115377

43 1 0 1.376462 0.000190 2.984287

44 1 0 -2.757488 -0.000146 2.141967

45 1 0 -2.416550 -0.000043 4.933744

46 1 0 0.131437 0.000165 5.242161

47 17 0 1.296261 -0.000074 -2.549475

48 8 0 2.100268 1.333129 -2.587707

49 8 0 2.100003 -1.333435 -2.587756

---------------------------------------------------------------------

Fe(III)-ClO_2__sm6_solv

Standard orientation:

---------------------------------------------------------------------

Center Atomic Atomic Coordinates (Angstrom)

Number Number Type X Y Z

---------------------------------------------------------------------

1 6 0 2.364535 -2.369903 0.223654

2 6 0 2.222700 2.496407 0.186146

3 6 0 -2.595855 2.356161 -0.647303

4 6 0 -2.454484 -2.520833 -0.606361

5 6 0 2.773676 -1.027632 0.301726

6 6 0 4.144462 -0.568007 0.443119

7 6 0 4.104154 0.805188 0.432752

8 6 0 2.708898 1.181566 0.285003

9 6 0 0.895137 2.891283 -0.044052

10 6 0 0.450127 4.263086 -0.158529

11 6 0 -0.904423 4.227280 -0.398255

12 6 0 -1.289124 2.832330 -0.428469

13 6 0 -2.998414 1.014725 -0.692288

14 6 0 -4.341931 0.548988 -0.978113

15 6 0 -4.302164 -0.823712 -0.966255

16 6 0 -2.934094 -1.205857 -0.673089

17 6 0 -1.122414 -2.916970 -0.381251

18 6 0 -0.657504 -4.286690 -0.331414

19 6 0 0.696988 -4.240570 -0.093305

20 6 0 1.062041 -2.843827 0.000680

21 7 0 1.927110 0.052513 0.231160

22 7 0 -0.181282 2.040496 -0.214982

23 7 0 -2.162635 -0.070694 -0.494197

24 7 0 -0.062065 -2.059096 -0.180810

25 26 0 -0.182448 -0.008650 0.104476

26 1 0 3.147626 -3.128255 0.321313

27 1 0 2.960841 3.299936 0.272385

28 1 0 -3.371345 3.109772 -0.814887

29 1 0 -3.184999 -3.320676 -0.761204

30 1 0 5.017980 -1.217868 0.523121

31 1 0 4.937925 1.506413 0.502417

32 1 0 1.096449 5.138301 -0.069036

33 1 0 -1.586887 5.066826 -0.543596

34 1 0 -5.198027 1.197740 -1.173058

35 1 0 -5.119094 -1.524256 -1.149634

36 1 0 -1.290204 -5.166364 -0.464034

37 1 0 1.393026 -5.075536 0.007620

38 7 0 0.053227 0.048650 4.356337

39 6 0 0.596876 0.031171 3.116334

40 7 0 -0.370848 0.015065 2.198281

41 6 0 -1.576070 0.022931 2.880008

42 6 0 -1.324722 0.044183 4.234537

43 1 0 1.666616 0.030596 2.911929

44 1 0 -2.526098 0.013208 2.346670

45 1 0 -1.981701 0.056319 5.102135

46 1 0 0.576114 0.062925 5.232194

47 17 0 0.844082 -0.000111 -2.631375

48 8 0 1.584522 1.360740 -2.879218

49 8 0 1.705617 -1.295567 -2.836972

---------------------------------------------------------------------

Fe(III)-O-Cl-O_sm2

Standard orientation:

---------------------------------------------------------------------

Center Atomic Atomic Coordinates (Angstrom)

Number Number Type X Y Z

---------------------------------------------------------------------

1 6 0 1.860025 2.708747 0.217632

2 6 0 -2.816901 2.108130 -0.953917

3 6 0 -2.181176 -2.709999 -0.957427

4 6 0 2.242988 -2.081772 0.950047

5 6 0 0.541727 2.964609 -0.162570

6 6 0 -0.005022 4.285954 -0.387795

7 6 0 -1.334616 4.117318 -0.670959

8 6 0 -1.588963 2.692131 -0.641940

9 6 0 -3.046749 0.736240 -1.063471

10 6 0 -4.293483 0.154294 -1.514601

11 6 0 -4.101774 -1.201124 -1.558308

12 6 0 -2.745708 -1.442837 -1.112613

13 6 0 -0.912741 -2.956846 -0.431154

14 6 0 -0.372118 -4.278322 -0.189407

15 6 0 0.858601 -4.100243 0.383588

16 6 0 1.072708 -2.670944 0.472785

17 6 0 2.513998 -0.714948 0.930323

18 6 0 3.798901 -0.141701 1.258081

19 6 0 3.708738 1.202151 1.003845

20 6 0 2.362361 1.449179 0.543526

21 7 0 -0.438084 2.009523 -0.316240

22 7 0 -2.122341 -0.252418 -0.811973

23 7 0 -0.020582 -1.993082 -0.015492

24 7 0 1.645001 0.269370 0.507738

25 26 0 -0.189588 0.003312 -0.262601

26 1 0 2.543061 3.559710 0.289475

27 1 0 -3.648348 2.778627 -1.190034

28 1 0 -2.788935 -3.573912 -1.241188

29 1 0 3.037090 -2.746656 1.300825

30 1 0 0.568083 5.212871 -0.326891

31 1 0 -2.085105 4.876175 -0.899694

32 1 0 -5.185786 0.726158 -1.775590

33 1 0 -4.804910 -1.981016 -1.856114

34 1 0 -0.885319 -5.211227 -0.429374

35 1 0 1.576002 -4.855419 0.709422

36 1 0 4.663932 -0.713906 1.594955

37 1 0 4.482268 1.965585 1.096004

38 7 0 -1.185851 0.723385 3.810926

39 6 0 -0.532219 0.959533 2.641128

40 7 0 -0.912062 0.078868 1.724374

41 6 0 -1.839046 -0.753337 2.317193

42 6 0 -2.024230 -0.365046 3.626398

43 1 0 0.195417 1.756358 2.489406

44 1 0 -2.302400 -1.566605 1.760661

45 1 0 -2.657237 -0.751729 4.422958

46 1 0 -1.074243 1.256159 4.670694

47 8 0 0.358094 -0.059262 -1.802294

48 8 0 4.417321 -0.191502 -1.662179

49 17 0 2.902236 -0.260452 -2.268807

---------------------------------------------------------------------

Fe(III)-O-Cl-O_sm2_solv

Standard orientation:

---------------------------------------------------------------------

Center Atomic Atomic Coordinates (Angstrom)

Number Number Type X Y Z

---------------------------------------------------------------------

1 6 0 1.867563 2.716835 0.220214

2 6 0 -2.814706 2.105902 -0.937512

3 6 0 -2.162486 -2.714217 -0.962115

4 6 0 2.244541 -2.074576 0.990477

5 6 0 0.548735 2.968975 -0.160622

6 6 0 -0.005240 4.289752 -0.372718

7 6 0 -1.336419 4.117869 -0.649245

8 6 0 -1.585596 2.691247 -0.630771

9 6 0 -3.038581 0.732962 -1.054162

10 6 0 -4.285022 0.147445 -1.501919

11 6 0 -4.087415 -1.207720 -1.555060

12 6 0 -2.728523 -1.447287 -1.116488

13 6 0 -0.896935 -2.957306 -0.425916

14 6 0 -0.362798 -4.277166 -0.161604

15 6 0 0.860337 -4.096047 0.428979

16 6 0 1.079075 -2.666490 0.503278

17 6 0 2.517450 -0.707639 0.953413

18 6 0 3.793224 -0.128061 1.303695

19 6 0 3.705738 1.214906 1.036913

20 6 0 2.368857 1.457100 0.548466

21 7 0 -0.427947 2.010193 -0.320478

22 7 0 -2.107475 -0.254245 -0.814996

23 7 0 -0.004966 -1.990778 -0.011661

24 7 0 1.656404 0.273086 0.502855

25 26 0 -0.175600 0.005609 -0.260884

26 1 0 2.544662 3.570589 0.308100

27 1 0 -3.651333 2.774338 -1.158851

28 1 0 -2.770193 -3.579209 -1.241134

29 1 0 3.027201 -2.735666 1.372316

30 1 0 0.563824 5.218167 -0.300445

31 1 0 -2.093437 4.874761 -0.861689

32 1 0 -5.181623 0.717702 -1.750922

33 1 0 -4.789288 -1.989855 -1.849440

34 1 0 -0.878582 -5.210556 -0.393515

35 1 0 1.568294 -4.848860 0.779828

36 1 0 4.646003 -0.694331 1.680926

37 1 0 4.468642 1.985513 1.157262

38 7 0 -1.190338 0.719222 3.790533

39 6 0 -0.529348 0.963678 2.632668

40 7 0 -0.896760 0.081042 1.705379

41 6 0 -1.825849 -0.758559 2.291214

42 6 0 -2.020183 -0.370266 3.599573

43 1 0 0.193083 1.767274 2.498240

44 1 0 -2.284602 -1.574527 1.735225

45 1 0 -2.656430 -0.759228 4.392116

46 1 0 -1.087694 1.251469 4.654140

47 8 0 0.367133 -0.059088 -1.808968

48 8 0 4.385249 -0.225121 -1.825037

49 17 0 2.816887 -0.265729 -2.294888

---------------------------------------------------------------------

Fe(III)-O-Cl-O_sm4

Standard orientation:

---------------------------------------------------------------------

Center Atomic Atomic Coordinates (Angstrom)

Number Number Type X Y Z

---------------------------------------------------------------------

1 6 0 2.827542 -1.408865 0.814565

2 6 0 1.307797 3.145025 0.037338

3 6 0 -3.105551 1.470292 -1.143960

4 6 0 -1.555179 -3.091779 -0.444356

5 6 0 2.790550 -0.019165 0.706556

6 6 0 3.922671 0.843621 0.950997

7 6 0 3.496758 2.127262 0.732820

8 6 0 2.105882 2.044736 0.352352

9 6 0 -0.031888 3.077448 -0.346263

10 6 0 -0.848873 4.232256 -0.647331

11 6 0 -2.093730 3.760837 -0.971856

12 6 0 -2.033026 2.319126 -0.873493

13 6 0 -3.061504 0.075932 -1.071862

14 6 0 -4.176477 -0.793734 -1.362513

15 6 0 -3.734966 -2.080092 -1.172839

16 6 0 -2.353719 -1.990316 -0.762059

17 6 0 -0.226681 -3.026876 -0.024361

18 6 0 0.585150 -4.181861 0.297218

19 6 0 1.820227 -3.707996 0.653424

20 6 0 1.757646 -2.265082 0.547936

21 7 0 1.683179 0.731128 0.360054

22 7 0 -0.768844 1.918807 -0.487974

23 7 0 -1.958322 -0.667773 -0.705648

24 7 0 0.502529 -1.869653 0.142832

25 26 0 -0.109949 0.029049 -0.258282

26 1 0 3.777733 -1.864666 1.106464

27 1 0 1.769865 4.135105 0.085688

28 1 0 -4.050818 1.930823 -1.445202

29 1 0 -2.008363 -4.083335 -0.533757

30 1 0 4.917328 0.494015 1.232227

31 1 0 4.067393 3.055163 0.799203

32 1 0 -0.498678 5.265381 -0.611750

33 1 0 -2.982481 4.325206 -1.260045

34 1 0 -5.164990 -0.452771 -1.675661

35 1 0 -4.286370 -3.013959 -1.296492

36 1 0 0.240274 -5.216323 0.247456

37 1 0 2.705277 -4.270392 0.956379

38 7 0 -0.771568 0.522606 4.170627

39 6 0 -0.077321 0.591039 2.997485

40 7 0 -0.805633 0.109845 2.002136

41 6 0 -2.010315 -0.283708 2.543171

42 6 0 -2.011169 -0.034307 3.900669

43 1 0 0.933816 0.988072 2.905535

44 1 0 -2.791529 -0.716930 1.918976

45 1 0 -2.755493 -0.195410 4.679024

46 1 0 -0.437470 0.828027 5.082354

47 8 0 0.486438 -0.239075 -2.157373

48 8 0 3.229046 0.243203 -2.152164

49 17 0 2.045116 -0.707870 -2.567928

---------------------------------------------------------------------

Fe(III)-O-Cl-O_sm4_solv

Standard orientation:

---------------------------------------------------------------------

Center Atomic Atomic Coordinates (Angstrom)

Number Number Type X Y Z

---------------------------------------------------------------------

1 6 0 2.849056 -1.378690 0.854918

2 6 0 1.290624 3.157339 0.014981

3 6 0 -3.120154 1.436089 -1.124969

4 6 0 -1.507902 -3.111071 -0.444287

5 6 0 2.796379 0.009646 0.733004

6 6 0 3.908214 0.889632 1.007526

7 6 0 3.472280 2.167410 0.765323

8 6 0 2.094505 2.064672 0.344564

9 6 0 -0.048553 3.076908 -0.370389

10 6 0 -0.878455 4.221433 -0.671285

11 6 0 -2.124241 3.736922 -0.980178

12 6 0 -2.050960 2.297078 -0.874023

13 6 0 -3.057415 0.042706 -1.048625

14 6 0 -4.164897 -0.841021 -1.329561

15 6 0 -3.705292 -2.122262 -1.148709

16 6 0 -2.320551 -2.017395 -0.751183

17 6 0 -0.178665 -3.031473 -0.024585

18 6 0 0.641892 -4.176315 0.306427

19 6 0 1.867739 -3.687985 0.681666

20 6 0 1.792029 -2.247363 0.573455

21 7 0 1.691446 0.742915 0.340326

22 7 0 -0.777055 1.909359 -0.501489

23 7 0 -1.939475 -0.688869 -0.696193

24 7 0 0.538110 -1.865290 0.147330

25 26 0 -0.103647 0.026164 -0.236712

26 1 0 3.793638 -1.821269 1.182368

27 1 0 1.741217 4.151900 0.071859

28 1 0 -4.074658 1.884886 -1.412931

29 1 0 -1.950630 -4.106951 -0.532785

30 1 0 4.893169 0.556449 1.338892

31 1 0 4.023296 3.104948 0.856740

32 1 0 -0.538051 5.257853 -0.641316

33 1 0 -3.021733 4.291681 -1.259091

34 1 0 -5.161185 -0.510186 -1.627843

35 1 0 -4.246284 -3.062808 -1.265688

36 1 0 0.307728 -5.214009 0.255191

37 1 0 2.753427 -4.239735 1.001258

38 7 0 -0.779621 0.528651 4.168129

39 6 0 -0.072408 0.601674 3.008564

40 7 0 -0.792033 0.125524 1.997604

41 6 0 -2.005299 -0.267722 2.526852

42 6 0 -2.015212 -0.022268 3.885666

43 1 0 0.940017 0.998365 2.939016

44 1 0 -2.784892 -0.697099 1.898071

45 1 0 -2.766024 -0.183766 4.657319

46 1 0 -0.453736 0.829918 5.086210

47 8 0 0.443210 -0.224639 -2.196601

48 8 0 3.150950 0.334988 -2.430429

49 17 0 2.007080 -0.756289 -2.539847

---------------------------------------------------------------------

Fe(III)-O-Cl-O_sm6

Standard orientation:

---------------------------------------------------------------------

Center Atomic Atomic Coordinates (Angstrom)

Number Number Type X Y Z

---------------------------------------------------------------------

1 6 0 -0.881484 -3.344733 -0.511480

2 6 0 3.085605 -0.808492 0.778156

3 6 0 0.605265 3.357830 0.190899

4 6 0 -3.375483 0.824893 -1.075599

5 6 0 0.431626 -3.043575 -0.105551

6 6 0 1.490299 -4.009106 0.134340

7 6 0 2.608590 -3.295327 0.495610

8 6 0 2.235915 -1.891179 0.480326

9 6 0 2.766599 0.559615 0.743506

10 6 0 3.689503 1.642851 1.025389

11 6 0 2.993172 2.813794 0.862158

12 6 0 1.642575 2.450850 0.476030

13 6 0 -0.705744 3.053393 -0.215754

14 6 0 -1.754521 4.019667 -0.484629

15 6 0 -2.876011 3.307500 -0.837211

16 6 0 -2.518251 1.901706 -0.790143

17 6 0 -3.058259 -0.546670 -1.040497

18 6 0 -3.972226 -1.631695 -1.340849

19 6 0 -3.267953 -2.802629 -1.186026

20 6 0 -1.922030 -2.437504 -0.787902

21 7 0 0.913625 -1.777927 0.127107

22 7 0 1.535026 1.082802 0.426802

23 7 0 -1.200438 1.782946 -0.409497

24 7 0 -1.829217 -1.066933 -0.712526

25 26 0 -0.111261 0.024085 -0.272891

26 1 0 -1.119590 -4.405961 -0.637977

27 1 0 4.118613 -1.058437 1.039917

28 1 0 0.849472 4.420522 0.286916

29 1 0 -4.400928 1.080263 -1.361341

30 1 0 1.389186 -5.091105 0.027074

31 1 0 3.600934 -3.678598 0.741788

32 1 0 4.738735 1.515173 1.297743

33 1 0 3.358688 3.836246 0.974321

34 1 0 -1.640592 5.103134 -0.413464

35 1 0 -3.859302 3.694464 -1.112035

36 1 0 -5.016458 -1.508636 -1.635033

37 1 0 -3.624267 -3.825116 -1.326622

38 7 0 -0.993029 -0.806283 3.992565

39 6 0 -0.421747 -1.054082 2.780289

40 7 0 -0.767394 -0.116198 1.909616

41 6 0 -1.588579 0.767787 2.578698

42 6 0 -1.742959 0.353586 3.884929

43 1 0 0.225543 -1.904355 2.568669

44 1 0 -2.007947 1.636330 2.073157

45 1 0 -2.300556 0.764137 4.725136

46 1 0 -0.884834 -1.375467 4.829365

47 8 0 0.526738 -0.104020 -2.123346

48 8 0 3.061579 1.055105 -2.166524

49 17 0 2.139185 -0.132777 -2.607652

---------------------------------------------------------------------

Fe(III)-O-Cl-O_sm6_solv

Standard orientation:

---------------------------------------------------------------------

Center Atomic Atomic Coordinates (Angstrom)

Number Number Type X Y Z

---------------------------------------------------------------------

1 6 0 -0.671030 -3.385008 -0.473977

2 6 0 3.141135 -0.601144 0.787515

3 6 0 0.420244 3.407933 0.128647

4 6 0 -3.419480 0.619864 -1.062553

5 6 0 0.623135 -2.998333 -0.076243

6 6 0 1.734646 -3.897075 0.181942

7 6 0 2.807486 -3.114545 0.541356

8 6 0 2.355022 -1.735166 0.503078

9 6 0 2.744686 0.746125 0.723206

10 6 0 3.592048 1.884970 1.021561

11 6 0 2.828540 3.013049 0.836472

12 6 0 1.511422 2.567807 0.422393

13 6 0 -0.871711 3.017213 -0.266914

14 6 0 -1.980489 3.912576 -0.534475

15 6 0 -3.061651 3.128140 -0.863957

16 6 0 -2.620479 1.747853 -0.803763

17 6 0 -3.022318 -0.730881 -1.012817

18 6 0 -3.870510 -1.874026 -1.293310

19 6 0 -3.093042 -2.998335 -1.134545

20 6 0 -1.768041 -2.547288 -0.752531

21 7 0 1.031298 -1.702566 0.137125

22 7 0 1.491119 1.193885 0.369575

23 7 0 -1.289290 1.713547 -0.441907

24 7 0 -1.759691 -1.171080 -0.691536

25 26 0 -0.112665 0.023457 -0.238379

26 1 0 -0.844371 -4.460251 -0.582311

27 1 0 4.179744 -0.788836 1.076700

28 1 0 0.594904 4.484038 0.223785

29 1 0 -4.461527 0.812759 -1.335494

30 1 0 1.693947 -4.984526 0.094562

31 1 0 3.817295 -3.435435 0.804055

32 1 0 4.636724 1.821820 1.332108

33 1 0 3.124661 4.056050 0.963928

34 1 0 -1.932718 5.001469 -0.474068

35 1 0 -4.071294 3.450036 -1.126091

36 1 0 -4.923453 -1.817855 -1.576116

37 1 0 -3.386410 -4.042517 -1.259055

38 7 0 -0.942505 -0.805682 4.010663

39 6 0 -0.360479 -1.047119 2.807940

40 7 0 -0.733412 -0.125420 1.923241

41 6 0 -1.585958 0.738365 2.585574

42 6 0 -1.727423 0.325764 3.894394

43 1 0 0.312461 -1.880599 2.613721

44 1 0 -2.035174 1.590117 2.077187

45 1 0 -2.298898 0.722100 4.731763

46 1 0 -0.818663 -1.365627 4.853869

47 8 0 0.460633 -0.120907 -2.152591

48 8 0 2.901678 1.171628 -2.422860

49 17 0 2.095672 -0.175389 -2.571833

---------------------------------------------------------------------

Fe(III)-Cl-O-O_sm2

Standard orientation:

---------------------------------------------------------------------

Center Atomic Atomic Coordinates (Angstrom)

Number Number Type X Y Z

---------------------------------------------------------------------

1 6 0 2.635853 -2.265677 -0.913664

2 6 0 2.354848 2.579157 -0.684061

3 6 0 -2.399445 2.293159 0.271878

4 6 0 -1.940182 -2.523539 0.699129

5 6 0 2.963781 -0.911117 -0.972003

6 6 0 4.269390 -0.400410 -1.329598

7 6 0 4.197218 0.963457 -1.225669

8 6 0 2.842940 1.279860 -0.823554

9 6 0 1.027574 2.916799 -0.419792

10 6 0 0.525448 4.274988 -0.411533

11 6 0 -0.822522 4.195465 -0.184698

12 6 0 -1.133546 2.790302 -0.034672

13 6 0 -2.680247 0.946543 0.500137

14 6 0 -3.970854 0.439688 0.913769

15 6 0 -3.826957 -0.912479 1.071952

16 6 0 -2.455321 -1.227915 0.733975

17 6 0 -0.657829 -2.868482 0.273602

18 6 0 -0.192566 -4.231938 0.131209

19 6 0 1.084490 -4.163982 -0.358929

20 6 0 1.399598 -2.758476 -0.495948

21 7 0 2.109436 0.125151 -0.658443

22 7 0 0.005455 2.025738 -0.177322

23 7 0 -1.766014 -0.081124 0.403385

24 7 0 0.328661 -1.982733 -0.102985

25 26 0 0.157291 0.021203 -0.174907

26 1 0 3.405132 -2.989901 -1.196400

27 1 0 3.057672 3.400300 -0.853098

28 1 0 -3.220910 3.008880 0.365576

29 1 0 -2.614239 -3.339305 0.976256

30 1 0 5.119677 -1.019984 -1.620257

31 1 0 4.974928 1.704589 -1.419308

32 1 0 1.138435 5.162861 -0.577234

33 1 0 -1.553497 5.003057 -0.116659

34 1 0 -4.861345 1.054341 1.054967

35 1 0 -4.576830 -1.649011 1.366046

36 1 0 -0.787612 -5.116760 0.364851

37 1 0 1.765621 -4.979611 -0.608204

38 7 0 1.741272 -0.302774 3.644927

39 6 0 1.659854 -0.599986 2.322382

40 7 0 0.679792 0.104718 1.766907

41 6 0 0.110360 0.879963 2.757189

42 6 0 0.764915 0.636684 3.943641

43 1 0 2.302105 -1.308712 1.800726

44 1 0 -0.721034 1.548270 2.539999

45 1 0 0.630322 1.034510 4.947638

46 1 0 2.407811 -0.704821 4.300400

47 17 0 -0.400388 -0.061448 -2.319056

48 8 0 -3.497362 -1.023731 -2.234067

49 8 0 -4.000164 0.082408 -2.275618

---------------------------------------------------------------------

Fe(III)-Cl-O-O_sm2_solv

Standard orientation:

---------------------------------------------------------------------

Center Atomic Atomic Coordinates (Angstrom)

Number Number Type X Y Z

---------------------------------------------------------------------

1 6 0 2.619788 -2.298165 -0.909437

2 6 0 2.391499 2.553600 -0.687514

3 6 0 -2.369992 2.322001 0.271342

4 6 0 -1.957535 -2.501279 0.719127

5 6 0 2.960703 -0.946680 -0.972171

6 6 0 4.275331 -0.451042 -1.318009

7 6 0 4.218421 0.914459 -1.215123

8 6 0 2.863844 1.247614 -0.826004

9 6 0 1.065997 2.905045 -0.427343

10 6 0 0.580537 4.269463 -0.408724

11 6 0 -0.769304 4.205336 -0.181132

12 6 0 -1.098658 2.803340 -0.040971

13 6 0 -2.664023 0.977787 0.501252

14 6 0 -3.953812 0.487561 0.937306

15 6 0 -3.822898 -0.866141 1.100691

16 6 0 -2.460127 -1.199429 0.743380

17 6 0 -0.679668 -2.860161 0.289456

18 6 0 -0.222367 -4.228710 0.167200

19 6 0 1.054072 -4.176068 -0.328726

20 6 0 1.377886 -2.774953 -0.488660

21 7 0 2.113037 0.101005 -0.671630

22 7 0 0.030966 2.023939 -0.195635

23 7 0 -1.762950 -0.062229 0.391674

24 7 0 0.309861 -1.985354 -0.109740

25 26 0 0.165671 0.019067 -0.171804

26 1 0 3.386038 -3.031795 -1.174066

27 1 0 3.106125 3.366533 -0.844462

28 1 0 -3.179308 3.048687 0.381947

29 1 0 -2.633432 -3.307415 1.018006

30 1 0 5.122210 -1.082086 -1.593024

31 1 0 5.007914 1.646513 -1.394651

32 1 0 1.206243 5.150500 -0.562485

33 1 0 -1.489393 5.021406 -0.100325

34 1 0 -4.832658 1.114834 1.094871

35 1 0 -4.574188 -1.592486 1.415629

36 1 0 -0.819764 -5.105350 0.424198

37 1 0 1.733049 -4.998513 -0.560275

38 7 0 1.735545 -0.295104 3.626223

39 6 0 1.658888 -0.605264 2.312620

40 7 0 0.681629 0.103708 1.743677

41 6 0 0.115128 0.893528 2.728658

42 6 0 0.768815 0.652034 3.916017

43 1 0 2.302141 -1.324665 1.809092

44 1 0 -0.710493 1.568459 2.511957

45 1 0 0.638000 1.057865 4.917034

46 1 0 2.398999 -0.696880 4.288316

47 17 0 -0.400297 -0.060355 -2.372267

48 8 0 -3.636105 -1.033831 -2.215603

49 8 0 -4.068348 0.102540 -2.237861

---------------------------------------------------------------------

Fe(III)-Cl-O-O_sm4

Standard orientation:

---------------------------------------------------------------------

Center Atomic Atomic Coordinates (Angstrom)

Number Number Type X Y Z

---------------------------------------------------------------------

1 6 0 2.327080 -2.541936 -0.890475

2 6 0 2.661146 2.305155 -0.674645

3 6 0 -2.103824 2.569288 0.193430

4 6 0 -2.231181 -2.260247 0.745544

5 6 0 2.828553 -1.239181 -0.922733

6 6 0 4.193982 -0.900757 -1.249940

7 6 0 4.297857 0.463347 -1.147394

8 6 0 2.990022 0.952855 -0.778981

9 6 0 1.374403 2.795160 -0.463512

10 6 0 1.025747 4.202781 -0.494244

11 6 0 -0.325241 4.274972 -0.298160

12 6 0 -0.791678 2.912490 -0.125038

13 6 0 -2.538053 1.272349 0.472550

14 6 0 -3.874657 0.937035 0.904695

15 6 0 -3.895584 -0.419138 1.106889

16 6 0 -2.579015 -0.908949 0.773018

17 6 0 -1.011881 -2.756620 0.291387

18 6 0 -0.711756 -4.167432 0.143423

19 6 0 0.557043 -4.246799 -0.360796

20 6 0 1.033950 -2.884154 -0.497782

21 7 0 2.111096 -0.098714 -0.625481

22 7 0 0.256477 2.023664 -0.232543

23 7 0 -1.756650 0.137137 0.408732

24 7 0 0.063257 -1.988744 -0.101941

25 26 0 0.155382 0.017800 -0.182710

26 1 0 3.006678 -3.352907 -1.167743

27 1 0 3.461747 3.033164 -0.834673

28 1 0 -2.839496 3.375511 0.263083

29 1 0 -2.995976 -2.983902 1.041773

30 1 0 4.963020 -1.624743 -1.525480

31 1 0 5.168876 1.096373 -1.326288

32 1 0 1.737162 5.013836 -0.660032

33 1 0 -0.965028 5.158487 -0.260282

34 1 0 -4.684759 1.658044 1.026710

35 1 0 -4.728800 -1.049050 1.423117

36 1 0 -1.402839 -4.976672 0.386302

37 1 0 1.137499 -5.135390 -0.615739

38 7 0 1.606859 -0.502985 3.663965

39 6 0 1.458247 -0.836571 2.354730

40 7 0 0.669822 0.050330 1.756290

41 6 0 0.294807 0.981592 2.704309

42 6 0 0.872748 0.647243 3.908725

43 1 0 1.922629 -1.696007 1.872944

44 1 0 -0.359561 1.813023 2.448657

45 1 0 0.829591 1.110588 4.892494

46 1 0 2.164001 -1.015771 4.343580

47 17 0 -0.393747 -0.022312 -2.322031

48 8 0 -3.781207 0.302798 -2.278313

49 8 0 -3.660456 -0.898772 -2.138762

---------------------------------------------------------------------

Fe(III)-Cl-O-O_sm4_solv

Standard orientation:

---------------------------------------------------------------------

Center Atomic Atomic Coordinates (Angstrom)

Number Number Type X Y Z

---------------------------------------------------------------------

1 6 0 2.372341 -2.489667 -0.926468

2 6 0 2.629842 2.362412 -0.643259

3 6 0 -2.143286 2.533091 0.230042

4 6 0 -2.185032 -2.308716 0.734698

5 6 0 2.849785 -1.176896 -0.946785

6 6 0 4.212258 -0.813629 -1.262268

7 6 0 4.296211 0.551016 -1.139449

8 6 0 2.978386 1.016562 -0.773909

9 6 0 1.334595 2.826133 -0.420023

10 6 0 0.963844 4.228467 -0.418355

11 6 0 -0.389369 4.275186 -0.222174

12 6 0 -0.835502 2.902067 -0.081314

13 6 0 -2.554361 1.223829 0.486996

14 6 0 -3.877860 0.862231 0.938008

15 6 0 -3.874368 -0.496907 1.126081

16 6 0 -2.555848 -0.963197 0.767466

17 6 0 -0.958497 -2.779511 0.269830

18 6 0 -0.626444 -4.183196 0.120257

19 6 0 0.641132 -4.233304 -0.393251

20 6 0 1.086648 -2.860050 -0.533227

21 7 0 2.111824 -0.049625 -0.645339

22 7 0 0.226488 2.030953 -0.209969

23 7 0 -1.755478 0.100365 0.394054

24 7 0 0.094949 -1.986755 -0.135642

25 26 0 0.158024 0.020459 -0.178760

26 1 0 3.070342 -3.285892 -1.199233

27 1 0 3.419823 3.105793 -0.781893

28 1 0 -2.888928 3.326982 0.326159

29 1 0 -2.930251 -3.046702 1.044170

30 1 0 4.994549 -1.524060 -1.535383

31 1 0 5.160357 1.198896 -1.296486

32 1 0 1.664377 5.053562 -0.559032

33 1 0 -1.042529 5.147305 -0.158468

34 1 0 -4.694650 1.570372 1.087397

35 1 0 -4.690079 -1.142796 1.455211

36 1 0 -1.295313 -5.007417 0.374267

37 1 0 1.243479 -5.107766 -0.645763

38 7 0 1.613152 -0.554704 3.639938

39 6 0 1.470479 -0.880969 2.333852

40 7 0 0.675203 0.008812 1.737414

41 6 0 0.295749 0.933190 2.694788

42 6 0 0.876399 0.587003 3.894955

43 1 0 1.945955 -1.734869 1.855161

44 1 0 -0.362921 1.765069 2.453360

45 1 0 0.831894 1.039267 4.883543

46 1 0 2.174010 -1.070676 4.317311

47 17 0 -0.385095 0.016519 -2.376084

48 8 0 -3.858993 0.312799 -2.257673

49 8 0 -3.761970 -0.892050 -2.124986

---------------------------------------------------------------------

Fe(III)-Cl-O-O_sm6

Standard orientation:

---------------------------------------------------------------------

Center Atomic Atomic Coordinates (Angstrom)

Number Number Type X Y Z

---------------------------------------------------------------------

1 6 0 2.524279 -2.442446 -0.774943

2 6 0 2.524313 2.442406 -0.774973

3 6 0 -2.186675 2.442377 0.506159

4 6 0 -2.186762 -2.442368 0.505992

5 6 0 2.956125 -1.110149 -0.902728

6 6 0 4.295229 -0.687460 -1.268612

7 6 0 4.295236 0.687387 -1.268635

8 6 0 2.956138 1.110102 -0.902760

9 6 0 1.237406 2.888316 -0.425345

10 6 0 0.835691 4.278307 -0.296137

11 6 0 -0.489232 4.278438 0.063447

12 6 0 -0.901377 2.888657 0.154765

13 6 0 -2.622026 1.109618 0.615366

14 6 0 -3.952595 0.686938 1.010781

15 6 0 -3.952625 -0.686900 1.010720

16 6 0 -2.622072 -1.109602 0.615273

17 6 0 -0.901461 -2.888663 0.154627

18 6 0 -0.489336 -4.278448 0.063289

19 6 0 0.835607 -4.278329 -0.296219

20 6 0 1.237352 -2.888341 -0.425370

21 7 0 2.175012 -0.000016 -0.682682

22 7 0 0.165366 2.077505 -0.145531

23 7 0 -1.842653 0.000002 0.386402

24 7 0 0.165311 -2.077521 -0.145590

25 26 0 0.097386 -0.000004 -0.390494

26 1 0 3.270292 -3.217669 -0.976995

27 1 0 3.270331 3.217619 -0.977048

28 1 0 -2.930978 3.216269 0.718411

29 1 0 -2.931093 -3.216249 0.718179

30 1 0 5.122992 -1.360452 -1.500925

31 1 0 5.123008 1.360363 -1.500968

32 1 0 1.489102 5.137057 -0.462197

33 1 0 -1.136969 5.137136 0.250378

34 1 0 -4.777868 1.360790 1.248164

35 1 0 -4.777926 -1.360738 1.248045

36 1 0 -1.137098 -5.137140 0.250161

37 1 0 1.489013 -5.137085 -0.462265

38 7 0 1.953602 0.000104 3.723490

39 6 0 1.954007 0.000206 2.359312

40 7 0 0.712427 -0.000054 1.896165

41 6 0 -0.120068 -0.000334 2.996021

42 6 0 0.635907 -0.000242 4.150096

43 1 0 2.856978 0.000467 1.749903

44 1 0 -1.203040 -0.000582 2.882294

45 1 0 0.367324 -0.000387 5.205305

46 1 0 2.778023 0.000257 4.320279

47 17 0 -0.427401 0.000034 -2.574791

48 8 0 -3.788778 0.609330 -2.202025

49 8 0 -3.788736 -0.608967 -2.202096

---------------------------------------------------------------------

Fe(III)-Cl-O-O_sm6_solv

Standard orientation:

---------------------------------------------------------------------

Center Atomic Atomic Coordinates (Angstrom)

Number Number Type X Y Z

---------------------------------------------------------------------

1 6 0 2.485285 -2.427678 -0.894290

2 6 0 2.485529 2.441616 -0.874379

3 6 0 -2.161293 2.434743 0.580047

4 6 0 -2.176457 -2.434106 0.511110

5 6 0 2.889144 -1.096023 -1.008774

6 6 0 4.214973 -0.677204 -1.405264

7 6 0 4.214272 0.695138 -1.402622

8 6 0 2.888458 1.111006 -1.002950

9 6 0 1.210954 2.857944 -0.484645

10 6 0 0.816331 4.242414 -0.344198

11 6 0 -0.492447 4.240734 0.066274

12 6 0 -0.893647 2.855237 0.174103

13 6 0 -2.564268 1.102655 0.692408

14 6 0 -3.877598 0.683457 1.126724

15 6 0 -3.883116 -0.687900 1.103768

16 6 0 -2.572062 -1.103147 0.657680

17 6 0 -0.906523 -2.851055 0.108465

18 6 0 -0.507685 -4.235563 -0.020056

19 6 0 0.806968 -4.233444 -0.411243

20 6 0 1.206586 -2.847666 -0.522435

21 7 0 2.095010 0.006564 -0.761477

22 7 0 0.156043 2.026447 -0.166896

23 7 0 -1.778139 0.000842 0.416072

24 7 0 0.149663 -2.019323 -0.203499

25 26 0 0.121807 0.004582 -0.286163

26 1 0 3.225203 -3.200805 -1.118421

27 1 0 3.224258 3.216763 -1.095382

28 1 0 -2.894437 3.206867 0.828371

29 1 0 -2.915549 -3.208222 0.734274

30 1 0 5.031618 -1.357775 -1.651985

31 1 0 5.030318 1.377528 -1.646312

32 1 0 1.470218 5.095031 -0.535318

33 1 0 -1.140458 5.091641 0.283241

34 1 0 -4.683883 1.364240 1.404428

35 1 0 -4.694672 -1.371135 1.359085

36 1 0 -1.160802 -5.088467 0.172359

37 1 0 1.461392 -5.084258 -0.608456

38 7 0 2.107183 -0.038555 3.752558

39 6 0 2.070099 0.001289 2.393383

40 7 0 0.812609 -0.023248 1.963166

41 6 0 0.012899 -0.081021 3.087747

42 6 0 0.806931 -0.091492 4.217719

43 1 0 2.958662 0.046504 1.764787

44 1 0 -1.073499 -0.110888 3.009332

45 1 0 0.573932 -0.130689 5.280511

46 1 0 2.950472 -0.030546 4.325893

47 17 0 -0.522928 0.022567 -2.571154

48 8 0 -3.856132 0.641633 -2.173542

49 8 0 -4.003437 -0.565040 -2.132392

---------------------------------------------------------------------

Fe(III)-O-O-Cl_sm2

Standard orientation:

---------------------------------------------------------------------

Center Atomic Atomic Coordinates (Angstrom)

Number Number Type X Y Z

---------------------------------------------------------------------

1 6 0 -1.609319 -2.827403 0.599552

2 6 0 2.878181 -1.900633 -1.025300

3 6 0 1.961843 2.856016 -0.644118

4 6 0 -2.675752 1.906511 0.460712

5 6 0 -0.295358 -2.982039 0.157816

6 6 0 0.349730 -4.262222 -0.029921

7 6 0 1.605641 -4.005142 -0.514885

8 6 0 1.725985 -2.567916 -0.608379

9 6 0 3.017537 -0.513079 -1.060291

10 6 0 4.237478 0.175806 -1.420273

11 6 0 3.985977 1.515130 -1.280488

12 6 0 2.609796 1.638423 -0.850210

13 6 0 0.614171 3.002937 -0.315447

14 6 0 -0.056470 4.280011 -0.212129

15 6 0 -1.372358 4.014637 0.063612

16 6 0 -1.495533 2.577739 0.145047

17 6 0 -2.777895 0.526280 0.613313

18 6 0 -3.989277 -0.161431 0.998882

19 6 0 -3.677382 -1.491338 1.069130

20 6 0 -2.280677 -1.611710 0.708954

21 7 0 0.559002 -1.957280 -0.191346

22 7 0 2.034373 0.392639 -0.713136

23 7 0 -0.275539 1.973479 -0.088451

24 7 0 -1.742056 -0.371590 0.445621

25 26 0 0.128368 0.010325 -0.156596

26 1 0 -2.170522 -3.735873 0.836345

27 1 0 3.735686 -2.507115 -1.330391

28 1 0 2.545223 3.769067 -0.794538

29 1 0 -3.577688 2.504920 0.614927

30 1 0 -0.118409 -5.227065 0.173440

31 1 0 2.389827 -4.712485 -0.790557

32 1 0 5.158182 -0.317588 -1.736791

33 1 0 4.655718 2.357614 -1.462815

34 1 0 0.429176 5.247396 -0.352875

35 1 0 -2.196322 4.716296 0.204000

36 1 0 -4.947530 0.327866 1.179895

37 1 0 -4.326404 -2.332952 1.317303

38 7 0 1.900324 -0.212515 3.617221

39 6 0 1.824127 -0.451831 2.282009

40 7 0 0.715550 0.091782 1.792134

41 6 0 0.054715 0.700725 2.840063

42 6 0 0.784386 0.520411 3.993812

43 1 0 2.563549 -1.007554 1.706127

44 1 0 -0.890398 1.218170 2.684077

45 1 0 0.618173 0.836997 5.021760

46 1 0 2.650520 -0.521345 4.231674

47 8 0 -0.298705 -0.120112 -1.965750

48 8 0 -1.332742 0.543363 -2.397934

49 17 0 -3.039038 -0.548570 -2.493157

---------------------------------------------------------------------

Fe(III)-O-O-Cl_sm2_solv

Standard orientation:

---------------------------------------------------------------------

Center Atomic Atomic Coordinates (Angstrom)

Number Number Type X Y Z

---------------------------------------------------------------------

1 6 0 -1.585277 -2.822216 0.672029

2 6 0 2.859448 -1.904563 -1.083867

3 6 0 1.973960 2.854597 -0.640855

4 6 0 -2.665214 1.909905 0.488230

5 6 0 -0.281911 -2.978785 0.197567

6 6 0 0.363062 -4.258937 0.011739

7 6 0 1.603839 -4.004963 -0.516122

8 6 0 1.718141 -2.569739 -0.632703

9 6 0 3.000302 -0.517822 -1.115644

10 6 0 4.220728 0.168040 -1.480033

11 6 0 3.982897 1.507221 -1.314602

12 6 0 2.611983 1.635607 -0.867930

13 6 0 0.626388 2.999006 -0.310068

14 6 0 -0.040782 4.275589 -0.191171

15 6 0 -1.358146 4.010988 0.084516

16 6 0 -1.487437 2.574785 0.152550

17 6 0 -2.764217 0.530175 0.656694

18 6 0 -3.962515 -0.153116 1.090560

19 6 0 -3.645164 -1.482145 1.176088

20 6 0 -2.259040 -1.606080 0.775061

21 7 0 0.559745 -1.956346 -0.193560

22 7 0 2.025001 0.392460 -0.747859

23 7 0 -0.268419 1.966739 -0.098753

24 7 0 -1.733270 -0.368286 0.472940

25 26 0 0.138431 0.012095 -0.141545

26 1 0 -2.133176 -3.728061 0.946126

27 1 0 3.712257 -2.511704 -1.398931

28 1 0 2.561871 3.766822 -0.774486

29 1 0 -3.559182 2.513126 0.666772

30 1 0 -0.092977 -5.221319 0.250495

31 1 0 2.384724 -4.713072 -0.798366

32 1 0 5.134933 -0.329361 -1.808269

33 1 0 4.658220 2.347498 -1.485164

34 1 0 0.449370 5.242588 -0.317205

35 1 0 -2.177985 4.713855 0.241661

36 1 0 -4.913061 0.339594 1.301393

37 1 0 -4.280554 -2.319887 1.468825

38 7 0 1.944055 -0.199021 3.576876

39 6 0 1.858790 -0.452249 2.251243

40 7 0 0.743197 0.093404 1.762858

41 6 0 0.093824 0.716151 2.814264

42 6 0 0.838653 0.540629 3.958918

43 1 0 2.594882 -1.018032 1.682741

44 1 0 -0.849726 1.239137 2.670384

45 1 0 0.687647 0.866807 4.985907

46 1 0 2.700621 -0.505330 4.188282

47 8 0 -0.346057 -0.199866 -1.951006

48 8 0 -1.310915 0.510177 -2.425353

49 17 0 -3.195726 -0.518514 -2.564436

---------------------------------------------------------------------

Fe(III)-O-O-Cl_sm4

Standard orientation:

---------------------------------------------------------------------

Center Atomic Atomic Coordinates (Angstrom)

Number Number Type X Y Z

---------------------------------------------------------------------

1 6 0 1.220499 3.038611 0.532925

2 6 0 -3.022619 1.606228 -1.379387

3 6 0 -1.702584 -3.035149 -0.711152

4 6 0 2.703372 -1.576227 0.738821

5 6 0 -0.063146 3.040370 -0.011799

6 6 0 -0.809070 4.239576 -0.310169

7 6 0 -1.995807 3.842422 -0.871999

8 6 0 -1.976680 2.399769 -0.906955

9 6 0 -3.023622 0.211009 -1.366384

10 6 0 -4.130490 -0.607299 -1.797584

11 6 0 -3.765438 -1.912669 -1.583963

12 6 0 -2.432294 -1.891489 -1.033296

13 6 0 -0.385331 -3.040382 -0.249362

14 6 0 0.392394 -4.234677 -0.010419

15 6 0 1.644120 -3.820506 0.366722

16 6 0 1.619282 -2.376211 0.374295

17 6 0 2.677832 -0.185181 0.820039

18 6 0 3.797040 0.627396 1.243144

19 6 0 3.367642 1.926338 1.219879

20 6 0 1.996299 1.901185 0.759574

21 7 0 -0.791644 1.920073 -0.377684

22 7 0 -1.988074 -0.584502 -0.902998

23 7 0 0.372352 -1.916283 -0.000909

24 7 0 1.586217 0.607366 0.537509

25 26 0 -0.215712 0.008293 -0.192988

26 1 0 1.671257 4.009655 0.756639

27 1 0 -3.907396 2.112120 -1.775892

28 1 0 -2.186727 -4.002821 -0.870720

29 1 0 3.642198 -2.079127 0.984928

30 1 0 -0.451541 5.253286 -0.120976

31 1 0 -2.817717 4.460981 -1.236858

32 1 0 -5.063933 -0.219842 -2.209681

33 1 0 -4.336279 -2.820446 -1.787494

34 1 0 0.020899 -5.253172 -0.136686

35 1 0 2.516123 -4.426468 0.618211

36 1 0 4.782292 0.237162 1.500852

37 1 0 3.926532 2.833615 1.454713

38 7 0 -2.264570 0.207357 3.663406

39 6 0 -2.108962 0.391250 2.322281

40 7 0 -0.930092 -0.071301 1.932379

41 6 0 -0.299883 -0.570620 3.051588

42 6 0 -1.119671 -0.405898 4.148243

43 1 0 -2.853024 0.854353 1.673407

44 1 0 0.695415 -1.009297 2.986703

45 1 0 -1.000551 -0.659580 5.200332

46 1 0 -3.078780 0.475112 4.212684

47 8 0 0.873535 0.004942 -2.043879

48 8 0 1.897997 -0.696312 -2.255829

49 17 0 3.793581 0.411986 -2.227469

---------------------------------------------------------------------

Fe(III)-O-O-Cl_sm4_solv

Standard orientation:

---------------------------------------------------------------------

Center Atomic Atomic Coordinates (Angstrom)

Number Number Type X Y Z

---------------------------------------------------------------------

1 6 0 0.897411 3.264623 -0.317612

2 6 0 -3.382456 1.008666 -0.792079

3 6 0 -1.036333 -3.250716 -0.765796

4 6 0 2.741428 -0.951632 1.251277

5 6 0 -0.456930 3.023563 -0.563819

6 6 0 -1.442830 4.045931 -0.830892

7 6 0 -2.660846 3.415892 -0.875497

8 6 0 -2.410029 2.004161 -0.681441

9 6 0 -3.114527 -0.362048 -0.833195

10 6 0 -4.087915 -1.374506 -1.178250

11 6 0 -3.410813 -2.566169 -1.256718

12 6 0 -2.035920 -2.285088 -0.913986

13 6 0 0.223041 -2.991416 -0.226613

14 6 0 1.161515 -4.010248 0.183717

15 6 0 2.179838 -3.368737 0.841922

16 6 0 1.889008 -1.953865 0.789838

17 6 0 2.569627 0.403296 0.976782

18 6 0 3.569525 1.415190 1.220957

19 6 0 3.095663 2.584408 0.683937

20 6 0 1.783049 2.298727 0.156204

21 7 0 -1.061408 1.785930 -0.480090

22 7 0 -1.872938 -0.937977 -0.662153

23 7 0 0.695687 -1.737401 0.130252

24 7 0 1.481358 0.955292 0.328004

25 26 0 -0.177665 0.015140 -0.154079

26 1 0 1.259610 4.292165 -0.405562

27 1 0 -4.416388 1.326875 -0.950544

28 1 0 -1.289617 -4.288933 -0.995728

29 1 0 3.656677 -1.255730 1.765396

30 1 0 -1.218829 5.107729 -0.945076

31 1 0 -3.647030 3.850728 -1.046660

32 1 0 -5.146428 -1.179416 -1.358563

33 1 0 -3.798351 -3.555853 -1.503603

34 1 0 1.028552 -5.079096 0.009168

35 1 0 3.067798 -3.800040 1.307180

36 1 0 4.525014 1.230892 1.714590

37 1 0 3.569782 3.566890 0.660616

38 7 0 -1.211558 0.731838 3.737086

39 6 0 -0.718136 1.082604 2.530279

40 7 0 -0.750811 0.022979 1.716712

41 6 0 -1.284605 -1.037697 2.428540

42 6 0 -1.577437 -0.602395 3.700403

43 1 0 -0.352021 2.074803 2.273259

44 1 0 -1.415957 -2.019248 1.977796

45 1 0 -2.003654 -1.109703 4.563279

46 1 0 -1.299198 1.355176 4.539922

47 8 0 0.187143 -0.031787 -2.080608

48 8 0 1.244619 -0.295252 -2.717630

49 17 0 3.807939 -0.164379 -2.104113

---------------------------------------------------------------------

Fe(III)-O-O-Cl_sm6

Standard orientation:

---------------------------------------------------------------------

Center Atomic Atomic Coordinates (Angstrom)

Number Number Type X Y Z

---------------------------------------------------------------------

1 6 0 -1.356248 -3.068652 0.375133

2 6 0 3.050802 -1.645506 -1.190081

3 6 0 1.689826 3.034886 -0.751953

4 6 0 -2.701483 1.606071 0.815260

5 6 0 -0.032096 -3.110897 -0.092639

6 6 0 0.728689 -4.313209 -0.370574

7 6 0 1.969933 -3.914095 -0.807593

8 6 0 1.975994 -2.464289 -0.803401

9 6 0 3.078669 -0.237919 -1.219626

10 6 0 4.209662 0.573242 -1.622046

11 6 0 3.823506 1.887892 -1.501325

12 6 0 2.456167 1.886431 -1.022594

13 6 0 0.367430 3.084882 -0.273665

14 6 0 -0.415734 4.289487 -0.054876

15 6 0 -1.653940 3.884839 0.381806

16 6 0 -1.628519 2.432876 0.433363

17 6 0 -2.730933 0.201997 0.847169

18 6 0 -3.878424 -0.600870 1.219064

19 6 0 -3.503280 -1.916548 1.096590

20 6 0 -2.125418 -1.921447 0.646158

21 7 0 0.752260 -2.006182 -0.358720

22 7 0 2.032165 0.583476 -0.866693

23 7 0 -0.389979 1.986402 0.045718

24 7 0 -1.684756 -0.628609 0.512063

25 26 0 0.175888 -0.028434 -0.179004

26 1 0 -1.848902 -4.033804 0.529691

27 1 0 3.962587 -2.157597 -1.513688

28 1 0 2.174301 3.996744 -0.948588

29 1 0 -3.629375 2.114090 1.095891

30 1 0 0.353153 -5.331715 -0.253731

31 1 0 2.806663 -4.542548 -1.119109

32 1 0 5.172871 0.183428 -1.957162

33 1 0 4.410478 2.783094 -1.715985

34 1 0 -0.063392 5.308019 -0.229519

35 1 0 -2.514698 4.506525 0.635544

36 1 0 -4.849443 -0.202357 1.517956

37 1 0 -4.106996 -2.808090 1.276202

38 7 0 1.793045 -0.583256 3.814534

39 6 0 1.476496 -0.928483 2.536380

40 7 0 0.880887 0.086868 1.923735

41 6 0 0.809386 1.121675 2.833196

42 6 0 1.375832 0.721372 4.024546

43 1 0 1.684385 -1.900269 2.089117

44 1 0 0.356912 2.074655 2.563228

45 1 0 1.517316 1.228944 4.977226

46 1 0 2.255715 -1.182060 4.495435

47 8 0 -0.659117 0.201312 -2.106591

48 8 0 -1.741058 0.776183 -2.433783

49 17 0 -3.450384 -0.400651 -2.291155

---------------------------------------------------------------------

Fe(III)-O-O-Cl_sm6_solv

Standard orientation:

---------------------------------------------------------------------

Center Atomic Atomic Coordinates (Angstrom)

Number Number Type X Y Z

---------------------------------------------------------------------

1 6 0 -1.319991 -3.075902 0.481288

2 6 0 2.968682 -1.632266 -1.376824

3 6 0 1.614591 3.039930 -0.863982

4 6 0 -2.653234 1.591562 1.022912

5 6 0 -0.029922 -3.104558 -0.070602

6 6 0 0.712394 -4.299935 -0.404553

7 6 0 1.920913 -3.894627 -0.924309

8 6 0 1.924864 -2.448436 -0.913405

9 6 0 2.984739 -0.227081 -1.407812

10 6 0 4.075499 0.587639 -1.899589

11 6 0 3.692048 1.901510 -1.758068

12 6 0 2.365982 1.896202 -1.178160

13 6 0 0.329497 3.072808 -0.294135

14 6 0 -0.433294 4.269483 -0.005693

15 6 0 -1.634471 3.859637 0.527546

16 6 0 -1.607806 2.412597 0.565311

17 6 0 -2.675877 0.188356 1.044107

18 6 0 -3.784947 -0.621438 1.505729

19 6 0 -3.411617 -1.935861 1.354453

20 6 0 -2.072761 -1.932024 0.799534

21 7 0 0.731367 -1.990022 -0.382477

22 7 0 1.959763 0.589897 -0.976968

23 7 0 -0.401985 1.963366 0.070291

24 7 0 -1.649290 -0.633622 0.628951

25 26 0 0.213703 -0.022658 -0.057963

26 1 0 -1.791199 -4.044049 0.674565

27 1 0 3.856298 -2.142143 -1.762543

28 1 0 2.075312 4.005750 -1.091476

29 1 0 -3.550117 2.100796 1.387789

30 1 0 0.349090 -5.319046 -0.261096

31 1 0 2.739430 -4.517246 -1.289745

32 1 0 5.012925 0.198361 -2.300699

33 1 0 4.254340 2.799609 -2.019708

34 1 0 -0.091943 5.288817 -0.194447

35 1 0 -2.469791 4.477765 0.861174

36 1 0 -4.727538 -0.227542 1.889739

37 1 0 -3.987395 -2.832701 1.589737

38 7 0 2.022145 -0.575467 3.771765

39 6 0 1.617594 -0.955078 2.537147

40 7 0 1.023193 0.068810 1.920794

41 6 0 1.050795 1.140151 2.796679

42 6 0 1.674790 0.749390 3.961244

43 1 0 1.762363 -1.951498 2.122033

44 1 0 0.623406 2.104397 2.526906

45 1 0 1.897704 1.279838 4.885059

46 1 0 2.502696 -1.171242 4.446179

47 8 0 -0.722299 0.023439 -2.038126

48 8 0 -1.690514 0.782472 -2.290382

49 17 0 -3.785677 -0.315360 -2.561633

---------------------------------------------------------------------

Cbl-chlorite, bond via O (truncated model)

Standard orientation:

---------------------------------------------------------------------

Center Atomic Atomic Coordinates (Angstroms)

Number Number Type X Y Z

---------------------------------------------------------------------

1 27 0 -0.394550 -0.093428 0.239976

2 7 0 -1.602466 -0.177392 -1.209989

3 7 0 0.021228 1.763031 -0.117752

4 7 0 0.775656 -0.225438 1.766248

5 7 0 -1.084400 -1.810226 0.575991

6 6 0 -2.357592 -1.439946 -1.406374

7 6 0 -3.637305 -0.966218 -2.111042

8 6 0 -3.124614 0.247424 -2.923260

9 6 0 -1.986249 0.750964 -2.054678

10 6 0 -1.409480 2.053121 -2.094567

11 6 0 -0.499463 2.507854 -1.164082

12 6 0 0.032440 3.921905 -1.099350

13 6 0 0.567310 4.021001 0.338269

14 6 0 0.641616 2.576971 0.771608

15 6 0 1.284971 2.151087 1.938579

16 6 0 1.390586 0.822416 2.361194

17 6 0 2.203832 0.365589 3.552338

18 6 0 2.243409 -1.163803 3.369087

19 6 0 1.115890 -1.415083 2.390953

20 6 0 0.491785 -2.628884 2.187968

21 6 0 -0.663648 -2.770908 1.360857

22 6 0 -1.612000 -3.953848 1.316889

23 6 0 -2.507474 -3.633252 0.092455

24 6 0 -2.404280 -2.101097 -0.026381

25 7 0 2.099590 -2.315023 -2.226277

26 6 0 3.027735 -1.309092 -2.010314

27 6 0 0.974538 -2.000672 -1.542972

28 7 0 1.085303 -0.844378 -0.890791

29 6 0 2.380233 -0.374843 -1.153453

30 6 0 3.081853 0.765666 -0.720234

31 6 0 4.394151 0.936212 -1.170844

32 6 0 5.017458 0.001905 -2.033263

33 6 0 4.343854 -1.142952 -2.467179

34 1 0 -3.884872 1.024045 -3.104302

35 1 0 -0.157855 4.531113 0.997157

36 1 0 2.122531 -1.735356 4.301966

37 1 0 -2.091558 -4.105930 -0.814933

38 1 0 -3.164639 -1.583922 0.589831

39 1 0 6.047115 0.176573 -2.361219

40 1 0 4.955881 1.816753 -0.842798

41 1 0 -3.544436 -3.980116 0.213671

42 1 0 -1.778078 -2.087619 -2.094483

43 1 0 -2.722118 -0.060991 -3.907703

44 1 0 1.539218 4.531903 0.430113

45 1 0 3.196052 -1.479936 2.904101

46 1 0 2.628769 1.487327 -0.043354

47 1 0 4.814518 -1.878013 -3.125618

48 1 0 0.101972 -2.646928 -1.532755

49 1 0 2.229160 -3.154462 -2.789619

50 1 0 -1.772234 2.755632 -2.848276

51 1 0 0.833478 -3.493303 2.761656

52 1 0 3.195548 0.843683 3.579332

53 1 0 1.673783 0.649763 4.480877

54 1 0 1.786816 2.912643 2.540233

55 1 0 0.845976 4.035199 -1.840434

56 1 0 -0.746374 4.661157 -1.339404

57 1 0 -4.098028 -1.747884 -2.733498

58 1 0 -4.373007 -0.633128 -1.357843

59 1 0 -1.074140 -4.913108 1.239003

60 1 0 -2.192156 -3.979242 2.258953

61 8 0 -1.747281 0.484027 1.471737

62 17 0 -2.824043 1.710346 0.990914

63 8 0 -2.341649 3.195688 1.230667

---------------------------------------------------------------------

Cbl-chlorite, bond via Cl (truncated model)

Standard orientation:

---------------------------------------------------------------------

Center Atomic Atomic Coordinates (Angstroms)

Number Number Type X Y Z

---------------------------------------------------------------------

1 27 0 -0.371521 0.068480 0.143350

2 7 0 -1.373038 0.865411 -1.239234

3 7 0 0.505655 1.748948 0.560608

4 7 0 0.479714 -0.898550 1.573042

5 7 0 -1.549143 -1.351736 -0.165739

6 6 0 -2.409956 0.032537 -1.903480

7 6 0 -3.423934 1.074350 -2.395751

8 6 0 -2.519765 2.295702 -2.681567

9 6 0 -1.404079 2.103254 -1.672746

10 6 0 -0.508626 3.111800 -1.212650

11 6 0 0.357526 2.927257 -0.159430

12 6 0 1.258841 3.995765 0.419246

13 6 0 1.602621 3.439308 1.811083

14 6 0 1.224232 1.980309 1.692044

15 6 0 1.583590 1.004971 2.625904

16 6 0 1.270110 -0.355461 2.529426

17 6 0 1.755072 -1.411656 3.495943

18 6 0 1.419039 -2.721518 2.759730

19 6 0 0.400326 -2.276975 1.733706

20 6 0 -0.497146 -3.101330 1.092871

21 6 0 -1.527568 -2.596361 0.244703

22 6 0 -2.762589 -3.337804 -0.225234

23 6 0 -3.348660 -2.384687 -1.297071

24 6 0 -2.811562 -1.010366 -0.862340

25 7 0 1.776692 -1.794113 -2.874535

26 6 0 2.885455 -1.181089 -2.312419

27 6 0 0.689879 -1.447238 -2.145864

28 7 0 1.000571 -0.633873 -1.137698

29 6 0 2.387522 -0.446472 -1.199644

30 6 0 3.282054 0.275783 -0.388994

31 6 0 4.637278 0.252108 -0.730762

32 6 0 5.113713 -0.472909 -1.850249

33 6 0 4.244335 -1.207454 -2.661357

34 1 0 -3.024039 3.268230 -2.561893

35 1 0 0.984503 3.905003 2.601756

36 1 0 1.035828 -3.522081 3.410396

37 1 0 -2.949234 -2.643411 -2.293924

38 1 0 -3.472865 -0.528515 -0.116145

39 1 0 6.183661 -0.462568 -2.081240

40 1 0 5.349464 0.806938 -0.111688

41 1 0 -4.447179 -2.414112 -1.347931

42 1 0 -1.938956 -0.477184 -2.768149

43 1 0 -2.095518 2.263761 -3.703939

44 1 0 2.657695 3.566070 2.101852

45 1 0 2.308331 -3.114378 2.231729

46 1 0 2.937873 0.822872 0.486935

47 1 0 4.599082 -1.779041 -3.523279

48 1 0 -0.306002 -1.819277 -2.370563

49 1 0 1.765737 -2.411045 -3.686026

50 1 0 -0.564625 4.102414 -1.669618

51 1 0 -0.484984 -4.168470 1.325286

52 1 0 2.821568 -1.287751 3.740628

53 1 0 1.184755 -1.320564 4.439682

54 1 0 2.189564 1.323506 3.477088

55 1 0 2.164459 4.078037 -0.210954

56 1 0 0.773937 4.983740 0.434214

57 1 0 -3.995998 0.736302 -3.272776

58 1 0 -4.121908 1.312892 -1.575336

59 1 0 -2.527712 -4.347807 -0.599168

60 1 0 -3.445973 -3.454873 0.637605

61 17 0 -1.929244 0.824463 1.771503

62 8 0 -2.510057 -0.457995 2.437953

63 8 0 -2.992773 1.697109 1.033768

---------------------------------------------------------------------

Cbl-chlorite, bond via O (big model)

Standard orientation:

---------------------------------------------------------------------

Center Atomic Atomic Coordinates (Angstroms)

Number Number Type X Y Z

---------------------------------------------------------------------

1 27 0 -1.180526 -0.306602 -1.044015

2 7 0 -1.588978 1.474542 -0.556273

3 7 0 -2.945679 -0.887255 -0.473257

4 7 0 -0.569750 -2.071317 -1.595368

5 7 0 0.436408 0.450398 -1.682638

6 6 0 -0.481403 2.457942 -0.631382

7 6 0 0.329586 2.445487 0.673564

8 6 0 -1.266755 3.819160 -0.877417

9 6 0 -0.527249 5.050009 -0.339834

10 6 0 -1.577966 4.005928 -2.392849

11 6 0 -2.698043 5.008470 -2.662557

12 8 0 -2.722149 6.135927 -2.180954

13 7 0 -3.696052 4.551566 -3.463067

14 6 0 -2.644945 3.509977 -0.190958

15 6 0 -2.839962 3.916577 1.290821

16 6 0 -3.352706 5.356600 1.445965

17 6 0 -4.785052 5.467418 0.926131

18 8 0 -5.698622 4.867701 1.474255

19 7 0 -4.960615 6.229318 -0.185501

20 6 0 -2.758284 2.012207 -0.391735

21 6 0 -4.017171 1.301861 -0.344628

22 6 0 -5.264259 2.162262 -0.401734

23 6 0 -4.046016 -0.079005 -0.261413

24 6 0 -5.270417 -0.960024 0.090145

25 6 0 -6.085905 -0.394292 1.266645

26 6 0 -6.144685 -1.125757 -1.179324

27 6 0 -7.373990 -2.027053 -0.995430

28 8 0 -7.490022 -2.828407 -0.081094

29 7 0 -8.325779 -1.883324 -1.955196

30 6 0 -4.577764 -2.338625 0.394777

31 6 0 -4.297691 -2.667298 1.881642

32 6 0 -5.469624 -3.301563 2.632735

33 6 0 -5.370668 -3.086975 4.143129

34 8 0 -4.700482 -2.192098 4.641371

35 7 0 -6.105050 -3.945129 4.898367

36 6 0 -3.260221 -2.180557 -0.309696

37 6 0 -2.504192 -3.266389 -0.744420

38 6 0 -1.287312 -3.194625 -1.417224

39 6 0 -0.630847 -4.379179 -2.113419

40 6 0 -1.222049 -4.456552 -3.545378

41 6 0 -0.835353 -5.719932 -1.402536

42 6 0 0.833578 -3.869685 -2.219854

43 6 0 1.803449 -4.317143 -1.092256

44 6 0 2.596861 -5.595198 -1.423682

45 6 0 3.533747 -5.339392 -2.603866

46 8 0 3.145984 -5.469037 -3.757945

47 7 0 4.771614 -4.891364 -2.277864

48 6 0 0.654893 -2.364597 -2.163954

49 6 0 1.637936 -1.469495 -2.533233

50 6 0 2.864936 -1.996556 -3.249970

51 6 0 1.469438 -0.059600 -2.295719

52 6 0 2.397354 1.064899 -2.796957

53 6 0 2.112689 1.248505 -4.303654

54 6 0 3.908651 0.884222 -2.537102

55 6 0 4.238905 0.303320 -1.157931

56 6 0 5.717223 0.352941 -0.804859

57 8 0 6.402305 -0.657530 -0.678555

58 7 0 6.208685 1.607965 -0.629721

59 6 0 1.868796 2.260411 -1.933989

60 6 0 2.250672 3.668432 -2.407999

61 6 0 3.260058 4.341964 -1.477981

62 8 0 4.102093 3.701198 -0.859723

63 7 0 3.188246 5.692461 -1.402154

64 6 0 0.387970 1.915913 -1.798398

65 6 0 7.558679 1.837311 -0.165396

66 6 0 7.750582 1.454087 1.304275

67 6 0 9.092227 1.860979 1.878325

68 8 0 6.694855 2.142777 2.048991

69 8 0 6.349791 0.966585 4.324916

70 8 0 4.389562 2.264344 3.205432

71 15 0 5.613152 1.448379 2.966538

72 8 0 5.364385 0.037592 2.222135

73 6 0 4.487784 -0.964299 2.715047

74 6 0 3.063819 -0.463806 3.063536

75 8 0 2.617474 0.494612 2.149979

76 6 0 2.243135 -1.778722 2.894548

77 8 0 3.002944 -2.639721 2.081767

78 6 0 4.198646 -1.983593 1.617828

79 6 0 5.259321 -3.036885 1.364606

80 8 0 4.750069 -4.064767 0.534243

81 7 0 0.942776 -1.519282 2.303579

82 6 0 -0.149520 -1.011947 3.006826

83 6 0 0.718408 -1.277643 0.985179

84 7 0 -0.445971 -0.692532 0.776322

85 6 0 -1.013326 -0.471404 2.036039

86 6 0 -2.180109 0.183564 2.449335

87 6 0 -2.504401 0.227972 3.803579

88 6 0 -3.772287 0.914652 4.242635

89 6 0 -1.663156 -0.405631 4.770322

90 6 0 -2.101635 -0.467769 6.209267

91 6 0 -0.463582 -0.997381 4.370182

92 1 0 -0.315204 2.587737 1.548644

93 1 0 1.097156 3.230586 0.672613

94 1 0 0.838750 1.484746 0.797868

95 1 0 0.495230 5.094561 -0.740243

96 1 0 -0.438586 5.040635 0.753305

97 1 0 -1.044915 5.966649 -0.645301

98 1 0 -1.846797 3.036980 -2.838371

99 1 0 -0.686602 4.373039 -2.924809

100 1 0 -3.670154 3.634707 -3.890452

101 1 0 -4.440120 5.190799 -3.721642

102 1 0 -3.453098 4.003242 -0.741744

103 1 0 -1.911270 3.777357 1.859877

104 1 0 -3.581723 3.248839 1.754835

105 1 0 -2.687049 6.074848 0.949392

106 1 0 -3.388995 5.612525 2.515846

107 1 0 -4.183736 6.570326 -0.747527

108 1 0 -5.899125 6.287701 -0.565647

109 1 0 -5.157338 2.916280 -1.197511

110 1 0 -5.474319 2.717934 0.524912

111 1 0 -6.158300 1.584605 -0.649560

112 1 0 -6.551468 0.565593 1.021449

113 1 0 -5.455786 -0.239686 2.151142

114 1 0 -6.891590 -1.086431 1.532508

115 1 0 -5.533275 -1.563384 -1.988944

116 1 0 -6.473747 -0.143259 -1.549110

117 1 0 -8.237782 -1.230510 -2.722377

118 1 0 -9.142070 -2.483661 -1.915371

119 1 0 -5.152681 -3.167601 -0.035133

120 1 0 -3.964015 -1.767610 2.413123

121 1 0 -3.439430 -3.356952 1.925196

122 1 0 -5.539960 -4.378920 2.406940

123 1 0 -6.430176 -2.882183 2.297766

124 1 0 -6.691374 -4.660666 4.489409

125 1 0 -6.135204 -3.802379 5.901466

126 1 0 -2.945455 -4.251980 -0.603286

127 1 0 -0.749366 -5.290372 -4.087336

128 1 0 -1.025003 -3.528938 -4.103890

129 1 0 -2.308460 -4.632272 -3.512338

130 1 0 -0.514018 -5.698302 -0.351338

131 1 0 -0.276590 -6.511720 -1.921585

132 1 0 -1.895786 -6.013017 -1.424200

133 1 0 1.262897 -4.181057 -3.179573

134 1 0 1.252305 -4.439226 -0.146225

135 1 0 2.532610 -3.516024 -0.900834

136 1 0 1.923642 -6.410380 -1.718289

137 1 0 3.161647 -5.928636 -0.539271

138 1 0 5.017908 -4.652230 -1.318464

139 1 0 5.387179 -4.618420 -3.035654

140 1 0 3.716633 -2.190316 -2.577515

141 1 0 3.211340 -1.297794 -4.018019

142 1 0 2.656377 -2.938035 -3.768955

143 1 0 2.704306 2.078659 -4.719378

144 1 0 1.046065 1.429768 -4.501469

145 1 0 2.379157 0.342828 -4.863825

146 1 0 4.370783 1.878235 -2.634283

147 1 0 4.372208 0.259336 -3.312023

148 1 0 3.944876 -0.750810 -1.121829

149 1 0 3.685702 0.825635 -0.361887

150 1 0 5.553761 2.389965 -0.628871

151 1 0 2.313782 2.122943 -0.941416

152 1 0 1.367424 4.307271 -2.544603

153 1 0 2.739438 3.635877 -3.395601

154 1 0 2.495130 6.231148 -1.904753

155 1 0 3.868162 6.183128 -0.831056

156 1 0 -0.138438 2.115893 -2.740052

157 1 0 7.796890 2.902908 -0.302782

158 1 0 8.266707 1.242124 -0.763723

159 1 0 7.591212 0.370640 1.396455

160 1 0 9.162766 1.563498 2.934843

161 1 0 9.230539 2.951287 1.810283

162 1 0 9.906053 1.368232 1.324189

163 1 0 6.207787 1.592592 5.051653

164 1 0 4.942625 -1.457978 3.592312

165 1 0 2.995544 -0.092761 4.100463

166 1 0 3.049382 1.342587 2.400020

167 1 0 2.064374 -2.262296 3.867898

168 1 0 3.982376 -1.446716 0.684472

169 1 0 5.618252 -3.448202 2.328437

170 1 0 6.097353 -2.560943 0.836158

171 1 0 3.948105 -4.406671 0.955453

172 1 0 1.431586 -1.546999 0.212462

173 1 0 -2.840032 0.653401 1.731543

174 1 0 -4.254690 1.439223 3.405170

175 1 0 -3.581726 1.655267 5.035834

176 1 0 -4.483255 0.175240 4.642686

177 1 0 -2.287863 0.534821 6.627557

178 1 0 -1.353696 -0.966530 6.842542

179 1 0 -3.047379 -1.030984 6.276413

180 1 0 0.195605 -1.453952 5.111364

181 8 0 -1.950564 0.225804 -2.725310

182 17 0 -2.030993 -0.887866 -3.958728

183 8 0 -0.654368 -1.157278 -4.659311

---------------------------------------------------------------------

Cbl-chlorite, bond via Cl (big model)

Standard orientation:

---------------------------------------------------------------------

Center Atomic Atomic Coordinates (Angstroms)

Number Number Type X Y Z

---------------------------------------------------------------------

1 27 0 -1.201336 -0.312456 -0.943180

2 7 0 -1.646922 1.465154 -0.472856

3 7 0 -2.935047 -0.918358 -0.331884

4 7 0 -0.604649 -2.046385 -1.547370

5 7 0 0.336276 0.499641 -1.679357

6 6 0 -0.551555 2.471328 -0.515866

7 6 0 0.255482 2.454103 0.789689

8 6 0 -1.363075 3.824267 -0.737791

9 6 0 -0.665212 5.063268 -0.165607

10 6 0 -1.650373 4.032002 -2.250759

11 6 0 -2.785783 5.002526 -2.565577

12 8 0 -2.974768 6.051874 -1.957574

13 7 0 -3.587892 4.602652 -3.584377

14 6 0 -2.738586 3.475333 -0.065669

15 6 0 -2.958052 3.854725 1.421142

16 6 0 -3.548239 5.263134 1.588305

17 6 0 -4.994952 5.279473 1.095002

18 8 0 -5.859927 4.641862 1.678080

19 7 0 -5.232783 5.994265 -0.035411

20 6 0 -2.824500 1.982634 -0.280700

21 6 0 -4.069579 1.245388 -0.249259

22 6 0 -5.342635 2.063002 -0.346025

23 6 0 -4.067390 -0.134609 -0.172974

24 6 0 -5.283268 -1.070194 0.002433

25 6 0 -6.379436 -0.576545 0.955551

26 6 0 -5.828084 -1.314185 -1.433085

27 6 0 -6.899551 -2.403190 -1.552771

28 8 0 -7.304756 -3.063058 -0.608408

29 7 0 -7.371017 -2.575347 -2.816568

30 6 0 -4.567059 -2.371979 0.504029

31 6 0 -4.345392 -2.492257 2.033890

32 6 0 -5.473495 -3.166058 2.810732

33 6 0 -5.202988 -3.153489 4.315633

34 8 0 -4.411076 -2.377752 4.833453

35 7 0 -5.922261 -4.048691 5.041929

36 6 0 -3.226893 -2.218856 -0.154514

37 6 0 -2.449914 -3.291042 -0.572432

38 6 0 -1.281585 -3.186958 -1.322837

39 6 0 -0.660457 -4.348260 -2.083942

40 6 0 -1.382965 -4.416856 -3.455077

41 6 0 -0.775055 -5.704555 -1.382018

42 6 0 0.779135 -3.805881 -2.302061

43 6 0 1.831833 -4.226693 -1.237254

44 6 0 2.644240 -5.473173 -1.634656

45 6 0 3.545847 -5.139898 -2.822485

46 8 0 3.136190 -5.229178 -3.973008

47 7 0 4.773008 -4.662676 -2.499376

48 6 0 0.570882 -2.305931 -2.223990

49 6 0 1.500371 -1.381384 -2.661489

50 6 0 2.682844 -1.866031 -3.477112

51 6 0 1.350497 0.017653 -2.346071

52 6 0 2.287270 1.160560 -2.788355

53 6 0 1.962590 1.475776 -4.265612

54 6 0 3.801607 0.930958 -2.597646

55 6 0 4.179717 0.266384 -1.269434

56 6 0 5.675467 0.267404 -0.987369

57 8 0 6.337241 -0.764161 -0.921464

58 7 0 6.208191 1.505054 -0.809457

59 6 0 1.808060 2.292426 -1.814554

60 6 0 2.208646 3.727566 -2.183144

61 6 0 3.251611 4.301462 -1.226114

62 8 0 4.124415 3.600828 -0.726331

63 7 0 3.173402 5.631489 -0.984019

64 6 0 0.321523 1.975089 -1.696557

65 6 0 7.591878 1.696544 -0.438082

66 6 0 7.870333 1.336334 1.023780

67 6 0 9.254543 1.723758 1.501321

68 8 0 6.882386 2.063660 1.823398

69 8 0 6.546603 0.945992 4.128391

70 8 0 4.593423 2.253346 3.012395

71 15 0 5.799693 1.412444 2.770859

72 8 0 5.514466 -0.008202 2.058862

73 6 0 4.628462 -0.985209 2.580717

74 6 0 3.222043 -0.455274 2.953484

75 8 0 2.767434 0.494535 2.033497

76 6 0 2.382182 -1.761684 2.827667

77 8 0 3.097352 -2.635539 1.987514

78 6 0 4.298579 -2.007668 1.498441

79 6 0 5.334389 -3.080878 1.232497

80 8 0 4.804369 -4.084388 0.386072

81 7 0 1.061645 -1.495994 2.289082

82 6 0 -0.015558 -1.012768 3.033005

83 6 0 0.792106 -1.250345 0.981036

84 7 0 -0.387061 -0.680187 0.819715

85 6 0 -0.920651 -0.481915 2.095457

86 6 0 -2.092597 0.137266 2.544466

87 6 0 -2.382669 0.155068 3.906411

88 6 0 -3.668146 0.781346 4.381402

89 6 0 -1.493442 -0.458295 4.843662

90 6 0 -1.887110 -0.543021 6.294093

91 6 0 -0.289772 -1.014104 4.405180

92 1 0 -0.392223 2.575874 1.665813

93 1 0 1.005045 3.256770 0.794562

94 1 0 0.789903 1.506155 0.904078

95 1 0 0.360091 5.146828 -0.553137

96 1 0 -0.589246 5.038167 0.928267

97 1 0 -1.213142 5.966210 -0.459958

98 1 0 -1.875696 3.070559 -2.732530

99 1 0 -0.754752 4.426453 -2.758009

100 1 0 -3.395807 3.761521 -4.116536

101 1 0 -4.306928 5.237604 -3.914806

102 1 0 -3.550439 3.958602 -0.618366

103 1 0 -2.023843 3.757104 1.990324

104 1 0 -3.666196 3.144870 1.875980

105 1 0 -2.935248 6.017805 1.077136

106 1 0 -3.580550 5.516287 2.658750

107 1 0 -4.483635 6.362305 -0.618851

108 1 0 -6.178059 5.982057 -0.403121

109 1 0 -5.193204 2.913830 -1.026113

110 1 0 -5.679438 2.487488 0.612213

111 1 0 -6.166665 1.480742 -0.768098

112 1 0 -6.962688 0.245939 0.529953

113 1 0 -5.956986 -0.226659 1.907965

114 1 0 -7.079140 -1.398328 1.144547

115 1 0 -4.994782 -1.565561 -2.115139

116 1 0 -6.255880 -0.376781 -1.824886

117 1 0 -6.985132 -2.083630 -3.612146

118 1 0 -8.060093 -3.301300 -2.977422

119 1 0 -5.088700 -3.267983 0.148899

120 1 0 -4.138402 -1.507804 2.469978

121 1 0 -3.423050 -3.070434 2.204193

122 1 0 -5.628667 -4.199935 2.458510

123 1 0 -6.433180 -2.651205 2.650842

124 1 0 -6.600535 -4.669571 4.620550

125 1 0 -5.831790 -4.036402 6.051520

126 1 0 -2.862895 -4.285602 -0.412519

127 1 0 -0.898622 -5.179143 -4.084873

128 1 0 -1.355157 -3.445342 -3.969483

129 1 0 -2.440855 -4.688496 -3.321345

130 1 0 -0.361488 -5.696365 -0.363784

131 1 0 -0.251089 -6.475902 -1.964697

132 1 0 -1.827410 -6.018203 -1.314463

133 1 0 1.153008 -4.111268 -3.287043

134 1 0 1.341669 -4.374090 -0.261929

135 1 0 2.544614 -3.403042 -1.079223

136 1 0 1.981890 -6.290199 -1.947894

137 1 0 3.238456 -5.829235 -0.779570

138 1 0 5.038946 -4.485208 -1.531801

139 1 0 5.371284 -4.342673 -3.252453

140 1 0 3.582969 -2.052667 -2.869115

141 1 0 2.957938 -1.141829 -4.250967

142 1 0 2.463510 -2.805028 -3.995951

143 1 0 2.548135 2.337428 -4.621231

144 1 0 0.891236 1.674959 -4.417727

145 1 0 2.224241 0.625026 -4.909192

146 1 0 4.279068 1.920430 -2.656160

147 1 0 4.220574 0.346162 -3.427280

148 1 0 3.863150 -0.782779 -1.273609

149 1 0 3.677576 0.758087 -0.422238

150 1 0 5.571550 2.298739 -0.732483

151 1 0 2.264126 2.063539 -0.843396

152 1 0 1.334055 4.389053 -2.247438

153 1 0 2.675310 3.765082 -3.181021

154 1 0 2.463651 6.221189 -1.398331

155 1 0 3.877175 6.057810 -0.390354

156 1 0 -0.183926 2.236467 -2.630543

157 1 0 7.856039 2.750594 -0.613354

158 1 0 8.236455 1.065698 -1.070189

159 1 0 7.694001 0.258934 1.149003

160 1 0 9.387882 1.445562 2.557003

161 1 0 9.410749 2.809161 1.401759

162 1 0 10.019544 1.202602 0.905272

163 1 0 6.420873 1.585738 4.846094

164 1 0 5.090828 -1.479232 3.454025

165 1 0 3.182086 -0.065700 3.985052

166 1 0 3.223883 1.338695 2.253335

167 1 0 2.239266 -2.235646 3.811806

168 1 0 4.079182 -1.476179 0.563185

169 1 0 5.683546 -3.513433 2.190583

170 1 0 6.182971 -2.616919 0.710567

171 1 0 3.999437 -4.422062 0.804611

172 1 0 1.482183 -1.503962 0.182560

173 1 0 -2.785418 0.591912 1.847161

174 1 0 -4.212909 1.256743 3.552015

175 1 0 -3.490089 1.550477 5.150346

176 1 0 -4.318622 0.012177 4.825850

177 1 0 -2.117169 0.447985 6.717635

178 1 0 -1.095442 -0.999292 6.905793

179 1 0 -2.797465 -1.158569 6.385727

180 1 0 0.401666 -1.458488 5.123981

181 17 0 -2.409366 0.064003 -2.968235

182 8 0 -2.717322 -1.354766 -3.512225

183 8 0 -1.578296 0.983748 -3.918263

---------------------------------------------------------------------
